# Machine learning approaches in microbiome research: challenges and best practices

**DOI:** 10.3389/fmicb.2023.1261889

**Published:** 2023-09-22

**Authors:** Georgios Papoutsoglou, Sonia Tarazona, Marta B. Lopes, Thomas Klammsteiner, Eliana Ibrahimi, Julia Eckenberger, Pierfrancesco Novielli, Alberto Tonda, Andrea Simeon, Rajesh Shigdel, Stéphane Béreux, Giacomo Vitali, Sabina Tangaro, Leo Lahti, Andriy Temko, Marcus J. Claesson, Magali Berland

**Affiliations:** ^1^Department of Computer Science, University of Crete, Heraklion, Greece; ^2^JADBio Gnosis DA S.A., Science and Technology Park of Crete, Heraklion, Greece; ^3^Department of Applied Statistics and Operations Research and Quality, Polytechnic University of Valencia, Valencia, Spain; ^4^Center for Mathematics and Applications (NOVA Math), NOVA School of Science and Technology, Caparica, Portugal; ^5^Research and Development Unit for Mechanical and Industrial Engineering (UNIDEMI), Department of Mechanical and Industrial Engineering, NOVA School of Science and Technology, Caparica, Portugal; ^6^Department of Ecology, Universität Innsbruck, Innsbruck, Austria; ^7^Department of Microbiology, Universität Innsbruck, Innsbruck, Austria; ^8^Department of Biology, University of Tirana, Tirana, Albania; ^9^School of Microbiology, University College Cork, Cork, Ireland; ^10^APC Microbiome Ireland, Cork, Ireland; ^11^Department of Soil, Plant, and Food Sciences, University of Bari Aldo Moro, Bari, Italy; ^12^National Institute for Nuclear Physics, Bari Division, Bari, Italy; ^13^UMR 518 MIA-PS, INRAE, Paris-Saclay University, Palaiseau, France; ^14^Complex Systems Institute of Paris Ile-de-France (ISC-PIF) - UAR 3611 CNRS, Paris, France; ^15^BioSense Institute, University of Novi Sad, Novi Sad, Serbia; ^16^Department of Clinical Science, University of Bergen, Bergen, Norway; ^17^MetaGenoPolis, INRAE, Paris-Saclay University, Jouy-en-Josas, France; ^18^MaIAGE, INRAE, Paris-Saclay University, Jouy-en-Josas, France; ^19^Department of Computing, University of Turku, Turku, Finland; ^20^Department of Electrical and Electronic Engineering, University College Cork, Cork, Ireland

**Keywords:** microbiome data analysis, machine learning methods, preprocessing, feature selection, predictive modeling, model selection, AutoML, colorectal cancer

## Abstract

Microbiome data predictive analysis within a machine learning (ML) workflow presents numerous domain-specific challenges involving preprocessing, feature selection, predictive modeling, performance estimation, model interpretation, and the extraction of biological information from the results. To assist decision-making, we offer a set of recommendations on algorithm selection, pipeline creation and evaluation, stemming from the COST Action ML4Microbiome. We compared the suggested approaches on a multi-cohort shotgun metagenomics dataset of colorectal cancer patients, focusing on their performance in disease diagnosis and biomarker discovery. It is demonstrated that the use of compositional transformations and filtering methods as part of data preprocessing does not always improve the predictive performance of a model. In contrast, the multivariate feature selection, such as the Statistically Equivalent Signatures algorithm, was effective in reducing the classification error. When validated on a separate test dataset, this algorithm in combination with random forest modeling, provided the most accurate performance estimates. Lastly, we showed how linear modeling by logistic regression coupled with visualization techniques such as Individual Conditional Expectation (ICE) plots can yield interpretable results and offer biological insights. These findings are significant for clinicians and non-experts alike in translational applications.

## 1. Introduction

The microbiome is a highly diverse system that plays a significant role in human health. Its composition and function can vary widely among individuals, and can be influenced by several factors such as host age, lifestyle habits, environmental or nutritional factors. Dysbiosis, or an imbalance in the microbiome, has been linked to a variety of health conditions (Claesson et al., 2017). For example, the gut microbiome is involved in many important physiological processes, including digestion, immune function, and metabolism. Changes in gut microbiota have been linked to several diseases such as inflammatory bowel disease (Glassner et al., 2020), type 2 diabetes (Navab-Moghadam et al., 2017), and colorectal cancer (CRC) (Zeller et al., 2014), as well as to mental diseases such as schizophrenia through the gut-brain axis (Thirion et al., 2023). Microbiome science is now having important implications for drug development and personalized medicine (Behrouzi et al., 2019).

The microbiome research community has traditionally relied on bioinformatic methods in order to solve important challenges such as taxonomic classifications, metagenome assembly and phylogenetic binning (Claesson et al., 2017). The use of ML can further support clinical applications. The most common ML tasks in microbiome research involve disease diagnosis, prognosis or the response to treatment (Brouillette, 2023) based on the taxonomic or functional composition of samples (Ghannam and Techtmann, 2021). Another important task is to predict the response of the microbiome to drug treatments, different dietary interventions or environmental exposures based on its composition (Thirion et al., 2022). Moreover, ML can be used to discover diagnostic or prognostic biomarkers in the microbiome, that is, the informative features (i.e., genes, taxa or functions) that are most strongly associated with a disease, phenotype, environmental variable or treatment response. Biomarkers can, in turn, be used for early detection of a disease, patient stratification, and personalized medicine (Flemer et al., 2017; Cammarota et al., 2020; Ryan et al., 2020; Berland et al., 2023).

A comprehensive overview of the challenges and solutions associated with the application of statistical and ML techniques in human microbiome studies has recently, been provided by the ML4Microbiome COST action^1^ (Moreno-Indias et al., 2021). A subsequent review of the applications of ML in human microbiome studies (Marcos-Zambrano et al., 2021) addressed the challenges of microbiome data analysis, and the importance of feature selection in the development of robust and interpretable models.

In this work, we continue in this direction by highlighting the specific issues pertaining to optimization and standardizing of state-of-the-art ML techniques for microbiome data predictive analysis. We define a set of initial Standard Operating Procedures (SOPs) in the form of practical advices, outline areas suitable for automation, and describe processes on how to integrate everything into pipelines. This will facilitate the translational usage of the developed models by clinicians and non-experts. We consider numerous aspects, ranging from tasks, algorithms or combinations of algorithms, hyper-parameters, to performance estimation protocols for disease prediction. We operationalize these pipelines using shotgun metagenomic datasets of gut microbiome and demonstrate the power of automated machine learning techniques (AutoML) in finding the optimal pipeline.

## 2. ML tasks and associated analysis steps

### 2.1. Biological, methodological, and technical constraints for data analysts

While predictive modeling using ML has the potential to provide valuable insights to the biology of the microbiome, several challenges and limitations need to be addressed (Table 1). Data preparation, for example, is an essential first step to enable predictive modeling. It consists of the bioinformatic analysis conversion of sequencing reads to tables that quantify genes, operational taxonomic units (OTU) or more recently Amplicon Sequence Variants (ASVs), metagenomic species (MSP), or functional modules. Two main sequencing methods are used to obtain microbiome data, 16S rRNA sequencing and shotgun metagenomics. Both of them have advantages and drawbacks. Profiling microbial communities using amplified 16S rRNA genes involves sequencing this specific gene, which is present in all bacteria, in order to identify and quantify the types of bacteria in a sample. It is a straightforward and cost-effective method to profile the taxonomic composition of a microbial community. The weaknesses of this methodology are (Větrovský and Baldrian, 2013; Poretsky et al., 2014; Tremblay et al., 2015; Khachatryan et al., 2020): (i) its relatively low taxonomic resolution due to the conservation of the target gene, (ii) imprecise taxa quantification due to the bias induced by the PCR amplification step and the variable gene copy number between and within microbial species, (iii) lack of functional information and intra-species and/or intra-population gene heterogeneity. Shotgun sequencing involves sequencing all extracted DNA in a microbiome sample, which allows a higher taxonomic resolution of the microbes species/strains, along with functional information (Brumfield et al., 2020; Durazzi et al., 2021). Analysis using metagenomic species reconstructed from non-redundant reference gene catalogs allows specific identification and quantification of the microbial species (Plaza Oñate et al., 2019). On the other hand, shotgun metagenomics sequencing is a much more expensive technique that generates large and complex datasets, which can be difficult to process, analyze, or interpret (Liu et al., 2021). Shotgun sequencing is also less suitable for samples with relatively low bacterial biomass (e.g., intestinal biopsies), where 16S rRNA sequencing is able to amplify these genes.

**Table 1:** List of challenges/constraints associated with applying machine learning (ML) approaches to microbiome data.

Challenge/Constraint	Description
Data acquisition and preparation	The process of acquiring and preparing microbiome data for predictive modeling involves bioinformatic analysis to convert raw sequencing reads into quantification (feature) tables. There are challenges associated with the sequencing methods used (16S rRNA sequencing or Shotgun metagenomics). Sequence data and accompanying metadata are often shared only with a bare minimum of detail, which is not always adequate for replication and further exploration.
Variability and sparsity of microbiome data	Microbiome data exhibits high variability in read depths per sample, sparsity (excess of zeros), non-Gaussian distributions and compositionality. The dependency structures among microbial species further complicates analysis.
Preprocessing tasks	Preprocessing tasks such as cleaning, normalization and batch effect correction are crucial for reducing technical biases and rendering data suitable for ML models. Challenges include choosing appropriate threshold filters for read quality and sparsity reduction, selecting normalization methods based on the model’s assumptions, and accounting for experimental conditions.
Data dimensionality	Microbiome data is often high-dimensional, with more features (microbial genes or taxa) than samples. This can lead to overfitting and poor generalization, especially with small sample sizes. Feature filtering and selection methods are employed to reduce dimensionality, but different methods can yield different results, and correlated features can hinder selection.
Non-linearity	Several ML models assume a linear relation between response and predictors. Since non-linear relationships may exist both among and between features and the target, the selection of appropriate model is fundamental for analysis.
Interpretability of ML models	While ML models can identify predictive patterns, interpreting these patterns in a biological context can be challenging. Using inherently interpretable models (e.g., decision trees, linear regression) and integrating metadata, environmental data, or functional assays can enhance interpretability. Visualization techniques and explainable AI methods can also aid in understanding the relationships between features and outcomes. Nevertheless, there is usually an interpretability/performance trade-off, by which the most highly performing models are often harder to interpret.
Limited availability of methods and recommendations	There is a limited number of established methods and standardized approaches for tasks such as batch effect correction and feature selection. Further research and consensus are needed to address these limitations and provide more robust solutions.

The specificity of the generated microbiome data has several implications which depend on the sequencing techniques used: (1) The total reads per sample (or depths of coverage) can vary by orders of magnitude within a single sequencing run. Comparison across samples with different depths of coverage requires specific adjustments that depend on the sequencing technique and the purpose of the analysis. (2) Microbiome data are sparse (excess of zeros in the feature tables) because (i) many species may be present in one individual and not in others (ii) species are present but sub-dominant and not found at the depth of coverage for a given sample. This feature is present in both 16S and shotgun data, but tends to be more severe in shotgun data. (3) This excess of zero renders the statistical distribution of the quantifications far from gaussian and thus hampers the use of modeling approaches which assume Gaussianity. (4) In high throughput sequencing, the total read count represents a fixed-size, random sample of the DNA/RNA molecules within the underlying habitat. It is crucial to note that this count is independent of the absolute number of molecules in the sample and is therefore subject to total sum constraints. Consequently, alterations in the abundance of one sequence necessitate compensatory changes in the abundance of other sequences. The mathematical framework for these data types is compositional analysis, however its application to microbiome data and the consequences on ML models is still an active research area. (5) Microbiome data has a complex inter-dependency structure, where the species may interact with each other in many ways, including mutualism, parasitism, commensalism, and competition. For shotgun data, sequenced genes might belong to the same species and as such strongly correlated. Some variables may be correlated which requires special attention for some ML algorithms.

Following microbiome quantification in the form of raw quantification (feature) tables, the first major challenge for predictive modeling is the preprocessing of the tables in order to reduce technical biases and render the data suitable for ML modeling. This is because differences in preprocessing can have a significant impact on the performance of the models and may introduce biases into the analysis. Typical preprocessing tasks involve normalization, cleaning, and batch effect correction. Normalization is needed for reducing technical biases, such as sequencing depth, and for making samples and features comparable. To the latter, the normalization strategy should consider the compositional nature of microbiome data and appropriate transformations should be applied to avoid misleading results (Li, 2015; Odintsova et al., 2017; Calle, 2019). Accordingly, incorrect or absence of scaling can lead to poor performance or even model failure. For example, when a distance metric is used like in Support Vector Machines (SVMs), scaling must be performed. Similarly, Linear Discriminant Analysis or Gaussian Naive Bayes are statistically effective if only the model errors are Gaussian. Modeling approaches based on decision trees, like CART, random forest, boosted decision trees, do not make such assumptions and work comfortably on raw unscaled data as well. Data cleaning, on the other hand, involves removing outlier samples or features with the aim to improve the quality of the data and reduce the impact of the noise in the modeling process. The identified outliers require careful examination before taking the decision to eliminate them. In addition, feature cleaning by low-abundance filtering often improves the performance of ML models and renders more interpretable signatures. However, there is no universal consensus of the threshold filter value to apply. Finally, batch effect correction, or including batch information as a covariate, can help in avoiding spurious associations between microbial features or phenotypes and unmasking true biological variation. This is particularly important in the case of extensive studies that involve samples analyzed at different time points or sequenced in separate runs, as well as meta-analyses comprising multiple independent studies (Goh et al., 2017). To this date, only a limited number of methods exist for this purpose, and there is a general lack of established recommendations for standardized approaches (Dai et al., 2019; Ling et al., 2022; Wang and Lê Cao, 2023).

Another major challenge is data dimensionality. Microbiome data is high-dimensional, meaning that there are often many more features than samples, which can lead to overfitting and poor generalization of performance. Feature selection and prevalence/abundance filtering methods can help to reduce the dimensionality of the data and select the most informative features for ML models. However, filtering methods do not remove redundant features. Similarly, different feature selection methods can optimize different objective functions, which may be distinct from the objective functions used in the ML models, and can result in different sets of selected features. Highly correlated features also hamper the selection of the relevant features. All these factors may negatively impact the performance of the model. Furthermore, not all feature selection methods are able to scale up to the thousands of microbial genes or taxa present in different individuals. Along the same lines, not many ML algorithms can scale down to low sample sizes. Low sample size can limit the statistical power and generalizability of ML models. Special care must therefore be given to the performance estimation protocol used during training for the best predictive model. The bootstrap bias correction method, for example, is such an approach equipped to provide better results than traditional cross-validation methods particularly at low sample sizes, also reducing the variance in the estimates of model performance (Tsamardinos et al., 2018).

A final challenge is that although ML models can identify predictive patterns in the data, it is often difficult to interpret these patterns in a biological context. This can limit the utility of ML for generating hypotheses and guiding experimental research. One way to ensure interpretability is to choose predictive modeling algorithms that are inherently interpretable, such as decision trees, logistic regression or linear SVMs. These models have an intuitive connection between the input and the output making it easier to understand the relationship between the microbiome features and the outcome. However, there is usually a performance/interpretability trade-off in ML, by which more complex models (ensembles of trees, neural networks) show better predictive power, but their outputs are also harder to interpret. Another way to improve interpretability is by the combined use of feature selection and the integration between metadata, environmental data, or functional assays, to encourage the model to use a smaller number of features, making it more interpretable and at the same time provide a comprehensive understanding of the microbial community. Dimensionality reduction methods such as sparse Partial Least Squares regression (PLS) are highly interpretable and also provide a visual representation of the data and the model’s predictions. Explainable AI techniques such as feature importance, partial dependence plots, and SHAP values can also help to explain the model’s predictions and how they are influenced by the input features (Lê Cao et al., 2009).

### 2.2. ML steps, and appropriate algorithms to use

**Figure 1:**
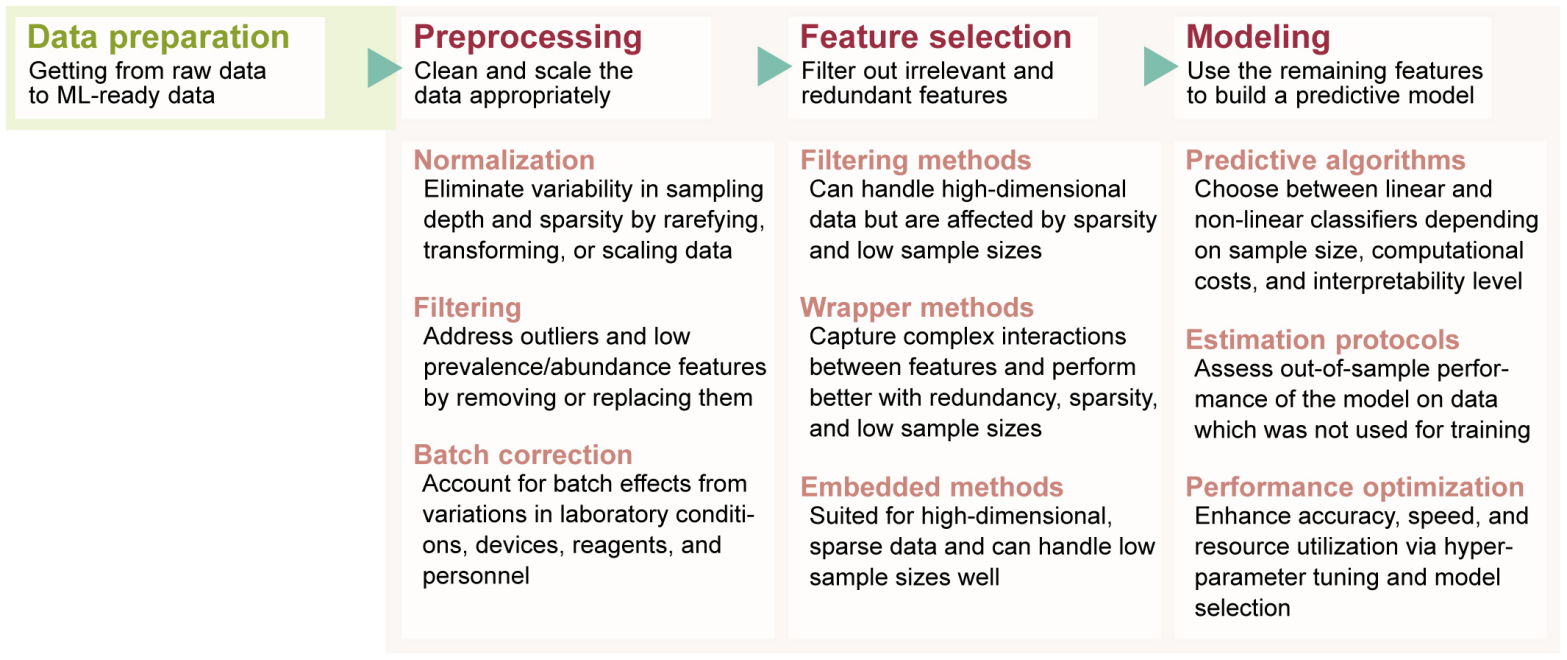
The typical process from data preparation to predictive model building, highlighting the methods to consider during each stage.

Once data has been collected and prepared for analysis, the typical process of building an ML model able to predict an outcome of interest consists of three consecutive steps: data preprocessing, feature selection and predictive modeling (Figure 1). For each of these steps there are several methods to consider so the optimal choice depends on the biological, methodological, and technical constraints of microbiome data (Table 2).

**Table 2:** Summary of machine learning (ML) algorithms for each workflow step.

Workflow step	Task	Algorithms
Data preprocessing	Handling outliers	Identify outliers by graphical methods (distribution or dimensionality reduction plots) or by statistical methods (Z-score).
		Investigate the cause of the outliers. if they are due to measurement errors or sample contamination, they should be removed.
	Filter out non-informative features	Threshold filtering, variance filtering or correlation-based filtering.
	Normalization	Rarefying.
		Scaling (different approaches: total sum, trimmed mean, geometric mean, upper quartile or data-driven threshold).
		Transformation (additive, centered or isometric log-ratio transformation).
	Batch correction	ComBat, limma, RUV, and PLSDA-batch.
Feature selection	Identify the most informative genes, taxa or functions	Filter methods: supervised (e.g., based on correlation, mutual information or ANOVA), unsupervised (e.g., based on dispersion and similarity measures).
		Wrapper methods: e.g., Recursive feature elimination (RFE), Statistically equivalent signatures (SES) or genetic algorithms.
		Embedded methods: feature selection during the model training process incorporating techniques such as Least absolute shrinkage and selection operator (LASSO) or Elastic net regularization.
Predictive modeling	Classification	Linear classifiers: logistic regression, linear discriminant analysis, partial least squares discriminant analysis (PLS-DA).
		Non-linear classifiers: SVMs, decision trees, random forests, artificial neural networks, gradient boosting, kernel PLS-DA.
	Performance estimation protocols: evaluate the quality of a predictive model	Holdout method: typically 70/30 split.
		K-fold Cross Validation protocol.
		Monte Carlo cross validation.
	Handling class imbalance	Stratified K-fold Cross Validation.
		Oversampling the minority class: random oversampling, synthetic oversampling.
		Undersampling the majority class: random undersampling, heuristic or learning models that try to find redundant examples for deletion.
		Class weighting.
	Optimization metrics	Threshold-independent measures: area under the receiver operating characteristic curve (AUROC), and area under the precision-recall curve (AUPRC).
		Threshold-dependent measures: accuracy, balanced accuracy, f1 score, Matthew’s correlation coefficient (MCC).
Model selection	Hyper-Parameter Optimization (HPO) or Combined Algorithm Selection and HPO (CASH)	Optimization techniques: random search, grid search, Bayesian optimization, and evolutionary algorithms. Early stopping, model checkpoints
Model interpretability	Explainable artificial intelligence (XAI)	Global explainer: feature importance (e.g. permutation feature importance).
		Local explainer: Local Interpretable Model-agnostic Explanations (LIME) and SHapley Additive exPlanations (SHAP).
		Individual Conditional Expectation (ICE) plots.

#### 2.2.1. Data preprocessing

Regarding data preprocessing, one needs primarily to consider how to normalize the data to enable biologically meaningful comparisons between samples or features. Normalization methods try to eliminate the variability in sampling depth and the sparsity of the data. Rarefying has been a widely used normalization method, especially for 16S rRNA data, in cases where there are significant differences in the library sizes (e.g., more than 10-fold) (Pereira et al., 2018). However, rarefying may not always be an ideal choice since it can reduce statistical power depending on the amount of samples being removed and it does not address all challenges of compositional data (McMurdie and Holmes, 2014).

Alternatives to rarefying are scaling and transformation. However, these are not recommended to be used at the same time, as this practice can invalidate the data, e.g., rescaling may preserve the original distributions but transformation may not (Lovell et al., 2015). Scaling involves finding a sample-specific factor, i.e., a fixed value or proportion, to multiply the matrix counts. Transformation methods, on the other hand, will replace values with the normalized ones. Several scaling approaches have been proposed based on the total sum, trimmed mean (Robinson and Oshlack, 2010), geometric mean (Love et al., 2014), upper quartile or a data-driven threshold (Paulson et al., 2013). But choosing the most effective one is difficult (McMurdie and Holmes, 2014; Weiss et al., 2017; Pereira et al., 2018; Lin and Peddada, 2020) because of the possible over- or under-estimation of fraction of zero counts and distortion of feature correlations across samples due to the data sparsity and differences in sequencing depths. Similarly, there are several transformation methods for microbiome data. Since microbiome datasets are essentially compositional, these methods follow Aitchison’s methodology for compositional data to convert the feature counts to log-ratios within each sample by applying an additive, centered or isometric log-ratio transformation (Aitchison, 1982; Egozcue et al., 2003). Again, a reference feature (gene, taxon, or function) is sought to be used for eliminating the effect of the sampling fraction. However, one should be cautious on how to replace the zeros during calculations for which there is no clear consensus. For example, zeroes can be eliminated by either incorporating a pseudo count or imputing values using probability or zero-inflated models. However, both approaches pose certain challenges. The addition of a pseudo count may artificially inflate low-abundance features while imputation introduces artificial values, which can distort the true distribution of the data and potentially obscure genuine biological variation (Kaul et al., 2017; Lubbe et al., 2021).

*Practical advice*: Normalization is problem- and algorithm-dependent. It is advisable to experiment with different types of normalization as part of the ML pipeline, and select the one who works the best. It is also important that some ML algorithms whose representation learning typically has a distance measure, might need specific types of normalization to work (e.g., SVMs require standardized data).

Apart from normalization, cleaning the data from outliers and unnecessary features is an essential preprocessing step to consider. Handling outliers requires to identify them typically by graphical methods (e.g., distribution or dimensionality reduction plots) or statistical ones (e.g., Z-score). For example, sanity checks for outlier detection include Principal Component Analysis of microbiome data that have been normalized. Subsequent ML tasks might also encompass the use of robust methods downweighting outlying observations during the estimation procedure (Kurnaz et al., 2018; Monti and Filzmoser, 2022; Kurnaz and Filzmoser, 2023). The cause of their outlierness must then be investigated. Outliers may be due to measurement errors, sample contamination, or biological variation. Understanding the cause can help determine the appropriate approach to handling outliers. If outlier samples are due to measurement errors or sample contamination, it may be appropriate to remove them from the dataset.

In terms of non-informative features or taxa that are biologically irrelevant or known contaminants, filtering can be based on thresholds on their abundance/prevalence, variance or correlation. Low-abundance or prevalence filtering involves eliminating features present in less than, e.g., 10% of the samples (Cao et al., 2021). Variance filtering involves removing features of zero or low variance across the samples as they are less likely to contribute to the overall variation in the data and may be less informative. The threshold for variance filtering can be determined based on the distribution of the variance in the data. Finally, based on the correlation coefficient or the mutual information between features, one can detect and filter out those that are highly associated with each other as they are redundant and may not provide additional information.

*Practical advice*: Perform exploratory data analysis that includes visualization of observations in a reduced dimension subspace for the inspection of outliers, correlation maps for the identification of highly correlated features, and descriptive statistics for the inspection of missing values and non-informative taxa.

After normalization and cleaning, one can perform batch correction to account for batch effects that may arise due to technical factors such as sequencing platform, library preparation, or batch processing. Several batch effect correction methods have been proposed in the literature, mainly for RNAseq and microarray data, such as ComBat, limma, and RUV that can be used to correct for batch effects in the microbiome domain (Wang and LêCao, 2020). Very recently, a new and effective approach for correcting batch effects called PLSDA-batch has been presented that can effectively correct for batch effects and improve the accuracy of downstream analyses (Wang and Lê Cao, 2023). Regardless of the chosen batch adjustment method, however, it is important to consider the statistical assumptions of the method, such as Gaussianity. It is possible that the data transformation applied prior to the batch adjustment may not satisfy these assumptions.

*Practical advice*: Before generating sequence data, make sure that samples are randomized so that whole study groups do not end up in separate batches. Visually inspect the post-sequencing impact of all possible batch effects on samples' distribution in a space of reduced dimension and in subsequent ML model performance, and if needed correct for this using recent appropriate tools.

#### 2.2.2. Feature selection

After preprocessing, feature selection is the next important step for microbiome data analysis in order to identify the most informative genes, taxa or functions. In principle, feature selection is the process of selecting a subset of features from a larger set of available features that are most important in predicting the outcome variable. The goal is to reduce the number of input features required to achieve good model performance, thereby improving the efficiency and interpretability of the model. This also helps to avoid overfitting, a common problem when analyzing high dimensional datasets where the model becomes too complex and starts to memorize the training data instead of learning general patterns.

Feature selection techniques can be broadly categorized into three types: filter methods, wrapper methods, and embedded methods. Filter methods can be either unsupervised (e.g., using dispersion and similarity measures) or supervised (e.g., based on correlation, mutual information or ANOVA), the latter evaluating the relevance of features to the outcome variable (Ferreira and Figueiredo, 2012), which can then be used to select the top-ranked features (Segata et al., 2011). Wrapper methods, such as recursive feature elimination, statistically equivalent signatures or genetic algorithms, employ statistical metrics too. However, they do so in combination with a predictive algorithm so as to select features based on their impact on the model’s performance (Lagani et al., 2017; Sanz et al., 2018). Embedded methods perform feature selection during the model training process by incorporating regularization techniques, such as L1 or L2 regularization, that automatically penalize the less important features. Nevertheless, there is still no consensus on which feature selection method should be used (Marcos-Zambrano et al., 2021).

The choice of the appropriate feature selection method remains an open problem because microbiome data poses numerous analysis challenges, such as noise, high dimensionality and small sample sizes, sparsity, and intercorrelated or redundant features. Filter methods can handle high-dimensional data relatively well, but they may not perform well in the presence of sparsity or low sample size. For example, correlation-based methods may suffer from false positives when the correlation is driven by sparse features or may select one feature from a correlated pair, resulting in suboptimal feature selection. Similarly, ANOVA may have low statistical power with few samples and may select redundant features that do not contribute additional information beyond what is already provided by other features. Wrapper methods, on the other hand, can better capture the complex interactions between features and may perform better than filter methods in the presence of redundancy, sparsity or low sample size. Their main drawback is that they can be computationally intensive and may not scale well to high-dimensional data. Embedded methods, such as least absolute shrinkage and selection operator (LASSO) or Elastic Net regularization, are well-suited for high-dimensional, sparse data and can handle low sample size relatively well (Tibshirani, 1996). These methods can perform both feature selection and regularization during model training, and can often identify a small number of highly relevant features that capture the underlying patterns in the data.

Regarding linearity, most filtering methods rely on linear statistical models to rank and select features based on their association with the response variable. Similarly, embedded methods typically use linear models to embed the features into a lower-dimensional space or to fit a regression model that selects the most informative features. To capture more complex relationships, mutual information is such a non-linear measure of the association between two random variables that can be used as a filter method. For example, the Minimum Redundancy Maximum Relevance (mRMR) selects features that have the highest mutual information with the target variable and the lowest mutual information with the previously selected features (Chen et al., 2016). Accordingly, any wrapper method that embeds a non-linear statistical metric can be used for capturing complex associations among features.

Lastly, certain feature selection techniques are inherently stochastic, implying that they may return different results in successive runs. Consequently, it is recommended to run each algorithm containing random elements multiple times, to obtain a more accurate understanding of its predictions. Alternatively, fixing the random seed in each run is guaranteed to provide consistent and deterministic results.

*Practical advice*: Consider testing a conservative filter method as a pre-screening stage in the feature selection task, or a more expensive multivariate method (e.g., embedded) to remove irrelevant and non-informative features in high-dimensional datasets. Another good general practice is to consider the objective function which is used in the feature selection and try to match it with the objective of the subsequently chosen modeling approach. For instance, feature selection based on Fisher score is suitable for linear discriminants, PCA is a good dimensionality reduction routine for Gaussian mixture models, recursive feature elimination is applicable for non-linear SVMs, etc.

#### 2.2.3. Predictive modeling

Lastly, the task of modeling involves selecting a predictive algorithm, a protocol for performance assessment, a protocol for model selection, and a metric for optimizing that performance. The choice of algorithm mainly depends on the problem type and the data characteristics. In the microbiome domain, classification problems are the most prevalent although efforts to address survival ones also exist. Regarding data characteristics there are several types of modeling algorithms, each having its strengths and weaknesses. Below, we explore methods that can handle challenges related to microbiome data such as scalability to high-dimensional data and small sample sizes, as well as interpretability. This will include both linear and nonlinear classifiers commonly employed and methods to estimate their performance.

Logistic regression and linear discriminant analysis, for example, are linear classifiers that can handle high-dimensional data, but they may be sensitive to overfitting when sample sizes are small (e.g., less than 100 per class). They may thus be good choices when the data is not too complex and the sample sizes are not too small. Partial Least Squares Discriminant Analysis (PLS-DA) is a good option for high-dimensional data with low sample sizes and benefits from multicollinearity, although care must be taken to avoid overfitting. On the other hand, SVMs are mainly nonlinear classifiers that can handle high-dimensional data but can be computationally expensive when the number of features is very large. Hence, they may be a good choice when the data is more complex, but the computational cost may be an issue. In contrast, decision trees and random forests are nonlinear classifiers that can handle high-dimensional data and are relatively robust to small sample sizes. However, they suffer from overfitting and instability when the trees are too deep or the data is noisy. Artificial neural networks and gradient boosting are also nonlinear classifiers that can handle high-dimensional data and are relatively robust to small sample sizes, but can be computationally expensive. Careful hyperparameter tuning is therefore important to avoid overfitting.

Unfortunately, due to the curse of dimensionality and the unknown patterns in the data, one cannot provide specific guidance on choosing a predictive modeling algorithm based on the number of features. Moreover, the so-called No Free Lunch Theorem in machine learning states that there is no single “best” method that can universally excel in solving all types of problems. The selection of an appropriate algorithm needs to consider the specific characteristics and constraints of the task at hand. Nevertheless, a combination of feature selection and a suitable performance estimation protocol can enhance a classifier’s performance in a high-dimensional setting (Wolpert, 2002). If interpretability is an important consideration, logistic regression, PLS-DA or decision trees are highly interpretable, while SVMs and artificial neural networks may be less so. Essentially, if feature selection is performed and techniques such as feature importance measures and visualization are used, insights into the behavior of even the most complex models can be gained.

*Practical advice*: For high-dimensional scenarios, as is the case with microbiome data, the choice of the model must consider the sample size and the desired computational cost and interpretability level. Coupling with a feature selection algorithm may improve prediction accuracy.

### 2.3. Building and evaluating ML workflows

#### 2.3.1. Performance estimation protocols

Performance estimation protocols are methods used to evaluate the quality of a predictive model. Their main purpose is to estimate the performance of the model on new, unseen data called out-of-sample performance or generalization error–the error that the model will obtain if hypothetically tested on the unseen data of infinite size. Estimation of the performance should not be confused with improving the performance which is the purpose of the model selection routine. The simplest protocol for performance assessment is the holdout method which involves splitting the available data into two parts, a training set and a test set; typically, a 70/30 split is used. The model is trained on the training set, and its performance is evaluated on the testing set. Holdout is suitable when the available data is sufficiently large, and the number of features is not too high relative to the number of samples. If the sample size is low, the performance on the test set will have a large variance. One way to reduce the variance is by repeating this protocol, each time by randomly assigning samples to training and test sets, and estimating the average model’s performance.

The well-known K-fold Cross-Validation protocol can be used for that. It involves dividing the data into K mutually exclusive, equally sized sets, or folds. Each time the model is trained on all folds but one that is held out for estimating the performance. If the sample size is very low, this protocol may be repeated several times with a different partitioning to folds, to further reduce the estimation variance. A typical value for the number of folds is 5 or 10, but this can be adjusted depending on the size of the data. In a similar manner, Monte Carlo cross-validation can be suitable when the available data is sufficiently large, and the number of features is high relative to the number of samples. This method can be useful when the data is noisy or there is a high degree of variability in the data, as it allows for multiple random splits to be generated. Taking K-fold Cross-Validation to the extreme, one can perform leave-one-out (LOO) cross validation, where all but a single sample is used for training and the performance is assessed on the remaining sample and averaged across all samples. LOO is known to be an almost unbiased performance assessment routine (Vapnik, 2006). The main advantage is its repeatability that comes from the deterministic nature of the routine. However, it can be a time-consuming process when the number of samples is larger.

A common characteristic of all protocols is that they use a portion of the data to train a model and the rest to evaluate its out-of-sample performance. In cases where the samples are plenty, losing some part of the data to estimation is acceptable. If not, as in the microbiome case, finding the right balance between training and test data is essential. Obviously, the best predictive model is—on average, not always—the one trained on all available data. However, since there is no more data left, how does one estimate its performance? The answer is to use one of the aforementioned protocols, i.e., evaluate the model performance on some partitioning protocol but train the final model on all available data. This process is called the “Train-Test-Retrain” procedure and presents a big change in perspective because it uses the performance of a suboptimal model as a proxy for the performance of the full model (Tsamardinos et al., 2022). As a result, the estimate is conservative, which is better than being overly optimistic. Essentially, during performance estimation we are not evaluating a specific model instance, but the entire ML pipeline that produces the final model.

Lastly, a typical methodological problem in predictive modeling is that of data leakage which can lead to optimistic or entirely invalid models. Data leakage occurs when performing data preprocessing or feature selection on the whole dataset before applying cross-validation. For example, when standardizing the data using the mean and standard deviation of the entire dataset, the rescaling process gains knowledge of the full data distribution, introducing bias on the rescaled values that can affect the performance of the algorithms on the cross-validation test sets. To avoid data leakage, therefore, the preprocessing, feature selection and predictive modeling must be performed together within each fold of the cross-validation and only apply them to the test fold on each cycle, ensuring the integrity of the evaluation process.

*Practical advice*: Evaluate the entire ML pipeline with cross-validation. For small sample sizes (e.g., 100 per class) use a Stratified, Repeated K-fold Cross Validation, of 4–5 repeats, with retraining on all data to produce the final model with a maximum K the number of samples in the rarest class so that at least one sample from each class gets into each fold.

#### 2.3.2. Class imbalance

A data characteristic that often appears in the microbiome domain is class imbalance where the number of samples in one class is much smaller than the number of samples in the other classes. Class imbalance can be problematic and lead to biased models that underperform on the minority class. One technique to alleviate this is the stratification of samples to cross-validation folds, namely, stratified K-fold Cross-Validation. This entails partitioning the data with the extra constraint that the distribution of the outcome in each fold is close to the distribution of the outcome in all samples. Other ways to compensate for the class imbalance include oversampling the minority class, undersampling the majority class and class weighting. Oversampling methods include random oversampling, where instances from the minority class are randomly duplicated, and synthetic oversampling, where new instances are in-silico synthesized from existing ones of the minority class, referred to as data augmentation, e.g., SMOTE (Chawla et al., 2002). General concern with oversampling is the increase of likelihood of overfitting due to exact or synthetic copies of the existing data (Fernández et al., 2018). Undersampling methods include random undersampling, where instances from the majority class are randomly removed, and methods that involve heuristics or learning models trying to find redundant examples for deletion or useful examples for non-deletion. However, removing too many samples from the majority class can be a problem, especially if the dataset is small. Oversampling and undersampling techniques can potentially enhance model performance when applied either as preprocessing steps or as integral components of the model itself (Mihajlović et al., 2021).

On the other hand, class weighting regards the assignment of weights to the classes to balance their contributions to the loss function during training. By assigning higher weights to the minority class, the algorithm can redistribute its capacity to focus more on correctly predicting the minority class, thus improving the overall performance on the imbalanced dataset. However, finding the right weights can be quite challenging. This strategy, also known as cost-sensitive learning strategy encourages the model to focus on correctly predicting the minority class, as misclassifying instances of this class incurs a higher cost (Ling and Sheng, 2010).

Regardless of how class imbalance is approached when evaluating the performance of a model on an imbalanced dataset, it is important to use appropriate performance metrics that consider the imbalance (e.g., balanced accuracy; averaged versions of precision, recall, F1-score etc.).

*Practical advice*: Data stratification during performance estimation and appropriate choice of performance metric should be practiced. Test several over/under-sampling options is suggested but always validate the similarity between synthetic samples and actual data. Alternatively consider class weighing or cost-sensitive methods.

#### 2.3.3. Performance metrics

Performance measures play a crucial role in evaluating and quantifying the predictive capabilities of classifiers. For example, threshold-independent measures like Area Under the Receiver Operating Characteristic Curve (AUROC or AUC) and Area Under the Precision-Recall Curve (AUPRC) are advantageous in assessing overall classifier performance. The AUC quantifies the ability of a classifier to discriminate between positive and negative instances across all possible decision thresholds. In other words, it measures the classifier discriminative capacity. Intuitively, AUC denotes the probability that a randomly chosen positive instance is ranked by a classifier higher than a randomly chosen negative instance. AUC is robust to moderate class imbalance and useful when the relative costs of false positives and false negatives are equal (Bewick et al., 2004). In contrast, AUPR focuses on the precision-recall trade-off and is particularly useful in imbalanced datasets, when the positive class is of greater interest. It denotes the probability of correct detection of positive instances (Saito and Rehmsmeier, 2015). Threshold-dependent measures, on the other hand, assess classifier performance at a specific decision threshold between 0 and 1. Threshold tuning does not change the classifier quality but can improve the performance metric, while also being one of the simplest approaches to handle a severe class imbalance (Fernández et al., 2018). Accuracy, for example, calculates the proportion of correctly classified instances over the total number of instances. However, it can be misleading under class imbalance, as it may achieve a high accuracy score by simply predicting that all observations belong to the majority class (Akosa, 2017). In contrast, balanced accuracy measures the average accuracy obtained from both the minority and majority classes. However, it treats all misclassifications equally and does not provide information about the performance of the classifier on individual classes. The F1 score is defined as the harmonic mean of precision and recall, which considers both false positives and false negatives. Nevertheless, F1 score does not capture true negatives, which can be crucial in certain applications. In contrast, Matthew’s Correlation Coefficient considers all four outcomes of a binary classification, true positive, true negative, false positive, and false negative rates. This is especially useful when the class distribution is imbalanced or when the costs associated with different types of errors vary (Chicco and Jurman, 2020).

*Practical advice*: Selecting an appropriate performance metric depends on the specific requirements of the task, the prevalence of class imbalance, and the trade-offs between different types of classification errors. Although AUC is widely used, different metrics highlight different performance aspects. Using multiple ones may help in analysis and better understanding of the classifier performance.

#### 2.3.4. Hyperparameter tuning

Several different candidate algorithms should typically be tried for each of the analysis steps based on the aforementioned factors to find the optimal ML pipeline. Nonetheless, each algorithm comes with several settings, referred to as hyper-parameters, that need to be set before training. Examples of hyperparameters include the learning rate of a neural network, its early stopping or model checkpoint parameters, the regularization strength of a linear model, or the depth of a decision tree. Optimizing for these choices is called Tuning, or else, Hyper-Parameter Optimization (HPO) or Combined Algorithm Selection and HPO (CASH) (Thornton et al., 2013; Feurer et al., 2015). In a nutshell, HPO selects the best hyperparameter values to achieve optimal performance while CASH involves selecting the best machine learning algorithm and its hyperparameters. CASH aims to automate the process by searching over a large space of possible algorithm and hyperparameter combinations. This is particularly useful when there is no clear choice of algorithm, or when the performance of different algorithms is highly dependent on the choice of hyperparameters.

Both HPO and CASH require training and evaluating many different ML pipelines with different hyperparameters or algorithms. To this end, various optimization techniques have been proposed, such as random search, grid search, Bayesian optimization, and evolutionary algorithms, among others. These techniques aim to efficiently search the hyperparameter or algorithm space to find the best combination that optimizes the desired performance metric. Random and grid search are simple to implement and parallelize but can be inefficient for high-dimensional search spaces. Bayesian optimization and evolutionary algorithms are more efficient, can use past evaluations to guide the search and also handle non-continuous and non-convex search spaces. Evolutionary algorithms can also search for multiple optima but can be computationally expensive. The downside of Bayesian optimization is that it requires a well-defined prior over the search space and can be sensitive to the choice of function to determine the next set of hyperparameters to evaluate.

#### 2.3.5. Model selection process while tuning

When trying multiple ML pipelines, it is tempting to select as best the one with the highest estimated performance. Practitioners sometimes confuse or mix up the error estimation process with the error reduction process. The performance assessment aims to estimate the error while model selection aims to reduce the error. When these procedures are mixed up a selection bias occurs leading to the respective performance estimate becoming compromised (usually over-optimistic). This problem is called the “winner’s curse” and is conceptually equivalent to the multiple hypothesis testing problem in statistics (Jensen and Cohen, 2000). Essentially, this phenomenon occurs since each performance protocol simulates an ideal scenario by pretending that the test sets come from the future, but in reality, these test sets are used to select the winning model and thus the process that aimed to estimate the performance is now used to improve it. This problem becomes more pronounced in low sample sizes, where the optimism could be as much as 20 AUC points (Ding et al., 2014; Tsamardinos et al., 2014). Therefore, appropriate performance estimation protocols should be used to correct for the winner’s curse.

The simplest solution to this problem is to hold out a second set of samples to be used for model selection. That is, extend the Train-Test protocol into the Train-Validate-Test protocol. The samples in the Validation may be used several times, but only for selecting the best model while those in the test set are used once, for performance estimation. Obviously, as before, this procedure is preferable when the sample size is large. In cases of low sample sizes, several alternatives have been proposed such as the nested cross validation (Salzberg, 1997), the Tibshirani-Tibshirani procedure (Tibshirani and Tibshirani, 2009) and the Bootstrap bias corrected cross validation (BBC-CV) (Tsamardinos et al., 2018) among others (Ding et al., 2014).

The nested cross validation involves a double loop procedure, where an inner cross-validation loop is run over the training data and is used for hyperparameter tuning, and an outer one for estimating the performance. Although nested cross-validation is very useful when the dataset is small and the number of hyperparameters is large it is computationally very expensive. The Tibshirani and Tibshirani method does not employ a separate hold out set. Rather it employs traditional K-fold cross-validation estimates to calculate the bias and subtract it from the performance estimates. A similar, but computationally more efficient method that has smaller variance and bias, is the BBC-CV method that was recently presented (Tsamardinos et al., 2018). Here, in order to calculate the bias, bootstrap resampling is employed on the pooled out-of-sample estimates collected during cross-validation of multiple pipelines.

*Practical advice*: Combined Algorithm Selection and HPO allows finding the optimal ML pipeline when this combines different algorithms and hyperparameters. Start exploring the space by grid or random search, and always correct for the “winner’s curse.” If the sample size is sufficient, use nested-CV due to its simplicity of implementation, otherwise use BBC-CV.

#### 2.3.6. AutoML: challenges and best practices

The above information suggests that implementing a complete machine learning workflow typically requires a substantial amount of skilled manual effort. In addition to being time-consuming, it also requires an expert to make informed decisions about which methods to incorporate into the pipelines. However, the lack of such experts and the associated high costs have paved the way for the emergence of automated machine learning (AutoML) (Hutter et al., 2019). AutoML aims to automate various stages of the machine learning process, including data preprocessing, feature selection, model training, hyperparameter tuning, and model evaluation. By doing so, AutoML enables objective and data-driven analysis decisions, resulting in high-quality models that can be utilized even by inexperienced users (Xanthopoulos et al., 2020).

AutoML is frequently used synonymously with the aforementioned CASH and HPO approaches that focus on solving a particular optimization problem. However, these solely aim to deliver predictive models and do not encompass the entire machine learning workflow necessary for microbiome data analysis. While various AutoML systems such as the well-known auto-sklearn (Feurer et al., 2022) or GAMA (Gijsbers and Vanschoren, 2019) exist, only TPOT (Olson and Moore, 2019) and JADBio (Tsamardinos et al., 2022) have the capability to extend their functionality to include the feature selection step. Notably, JADBio goes even further by encompassing all the necessary steps, including the estimation of out-of-sample predictive performance, which most AutoML systems do not automate, thereby providing a comprehensive solution for the ML analysis of microbiome data.

While AutoML offers significant advantages by automating various steps of the machine learning workflow, it may also have certain challenges. Firstly, AutoML may lack transparency, making it challenging to understand and explain the underlying decisions made by the automated processes. This opacity can limit the ability to detect and address biases or errors. AutoML tools may also have limited customization options, as they are designed to cater to a wide range of users and tasks, restricting flexibility and domain-specific adaptations. Furthermore, it can increase computational cost due to extensive model exploration and lastly, relying solely on AutoML can diminish the essential role of human expertise and domain knowledge, which are crucial in understanding the context, interpreting results, and making informed decisions. It is therefore essential to strike a balance between the advantages of automation and the need for human involvement (Gijsbers et al., 2019; Romero et al., 2022).

*Practical advice*: AutoML is becoming increasingly popular, but most approaches primarily focus on solving the CASH problem to provide an optimal predictive model. As a result, researchers still need to decide on performance estimation methods and protect against the “winner’s curse”.

### 2.4. Model interpretability and explainability of results

Model explainability involves understanding how algorithms learn the relationship between inputs and outputs. In classification models, there are three main goals: to create an accurate model, to accurately estimate how good the model is and interpretability. However, there is often a tradeoff between these objectives whereby linear models are interpretable but may underperform compared to nonlinear models. Complex nonlinear models achieve better performance but are less interpretable. This lack of interpretability limits their use in biomedical research where understanding the classification process is crucial.

For this reason, explainable artificial intelligence is a growing field that focuses on explaining the output or decisions of ML models (Carrieri et al., 2021; Lombardi et al., 2021, 2022; Bellantuono et al., 2022). One prominent technique in this respect is the measurement of feature importance. Feature importance methods aim to quantify the contribution of each feature to the model’s predictions. Particularly, global methods provide an overall ranking of features while local methods try to explain the contribution of each feature to a specific prediction. For example, permutation importance is a global method that evaluates importance by disrupting the relationship between the feature and the true outcome. The underlying concept is simple: if permuting a feature’s values results in higher prediction error, it indicates its importance. Conversely, if permuting the feature does not affect the error, it is classified as unimportant. Regarding local methods, Local Interpretable Model-agnostic Explanations (LIME) is a technique that approximates model behavior with an interpretable (linear) model at the neighborhood around each individual prediction (Ribeiro et al., 2016). Similarly, SHapley Additive exPlanations (SHAP) is a local explainer algorithm that uses a concept from game theory called Shapley values (Lundberg and Lee, 2017). Shapley values measure how much each feature contributes to the prediction by considering all possible combinations of features in a fair share manner. SHAP can work with any kind of model and can show the impact of each feature visually. Finally, some methods combine a stepwise forward strategy to identify a minimal subset of interpretable variables from a permutation-based score of importance (Genuer et al., 2015).

Individual Conditional Expectation (ICE) plots also provide a way to explore and understand the relationship between a specific input feature and the output of a model, while considering the influence of other features (Tsamardinos et al., 2022). In an ICE plot, the x-axis represents the range of values for the chosen input feature. Each line in the plot corresponds to how the model prediction changes while varying all the remaining input features. In this way ICE plots help identify non-linear patterns, interactions, and heterogeneity in the model’s behavior across instances, aiding in model interpretation at the individual level.

*Practical advice*: Start with a simple, interpretable model; more complex models can be used to achieve better performance, for which model explanation techniques can be used, such as calculation of feature importances, LIME, SHAP values, and ICE plots.

## 3. Comparative evaluation of ML approaches

To showcase the effectiveness of various ML approaches in enhancing predictive performance, we collected a set of CRC benchmark data on a two-class (healthy/cancer) classification problem. To this direction, we first evaluated the effect of typical preprocessing steps such as normalization and filtering. Then we used AutoML, namely JADBio, to find the best performing and best interpretable pipelines in terms of feature selection and predictive modeling.

### 3.1. Description of the data

This dataset (Barbet et al., 2023) gathers 2090 human stool samples characterized by shotgun metagenomic sequencing from 13 public cohorts spanning nine countries (Table 3). This data provides the gut microbiota composition in healthy controls and patients with adenoma or CRC.

**Table 3:** Compilation of datasets from nine distinct countries, including 2,090 human stool samples characterized via shotgun metagenomic sequencing.

BioProject	Country	Nb all	Nb CRC
PRJDB4176	Japan	645	286
PRJEB10878	China	128	74
PRJEB12449	USA	104	52
PRJEB27928	Germany	82	22
PRJEB6070	France	156	53
PRJEB6070	Germany	43	38
PRJEB7774	Austria	156	46
PRJNA389927	USA	56	26
PRJNA389927	Canada	28	2
PRJNA397112	India	110	0
PRJNA447983	Italy	140	61
PRJNA531273	India	30	30
PRJNA608088	China	18	6
PRJNA429097	China	194	98
PRJNA763023	China	200	100
All cohorts	9 countries	**2090**	**894**

Data were prepared as follows. Sequencing data was downloaded from the European Nucleotide Archive. Reads were quality trimmed and filtered from sequencing adapters using fastp. Remaining contamination by the host genome was filtered out by mapping reads against the human reference genome (T2T-CHM13v2.0) with bowtie2. Microbial species identification and quantification was estimated according to both human gut reference gene catalog (IGC2, 10.4 M genes, Wen et al., 2017) and human oral gene catalog (8.4 M genes, Le Chatelier et al., 2021) with the METEOR software (Pons et al., 2010), and clustered into Metagenomic Species Pangenomes taxonomically and functionally annotated (Plaza Oñate et al., 2019).

### 3.2. Evaluation of the preprocessing steps

We evaluated the effect of two typical preprocessing steps on the performance of various standard ML algorithms implemented in a caret workflow (Kuhn, 2015): RF—Random Forest, PLS—Partial least square, Earth—spline regression (can be applied to classification also), Pam—Partition around medoids (normally a clustering algorithm), Glmboost—Gradient Boosting with Component-wise Linear Models, Glmnet—Generalized linear model with elastic net penalty, GBM—Gradient boosting machine. The data were split in a training set (75%) used to tune the hyperparameters of the models and a test set (25%) used to evaluate the model performance. The split of the data has been repeated 100 times (Fromentin et al., 2021).

**Figure 2:**
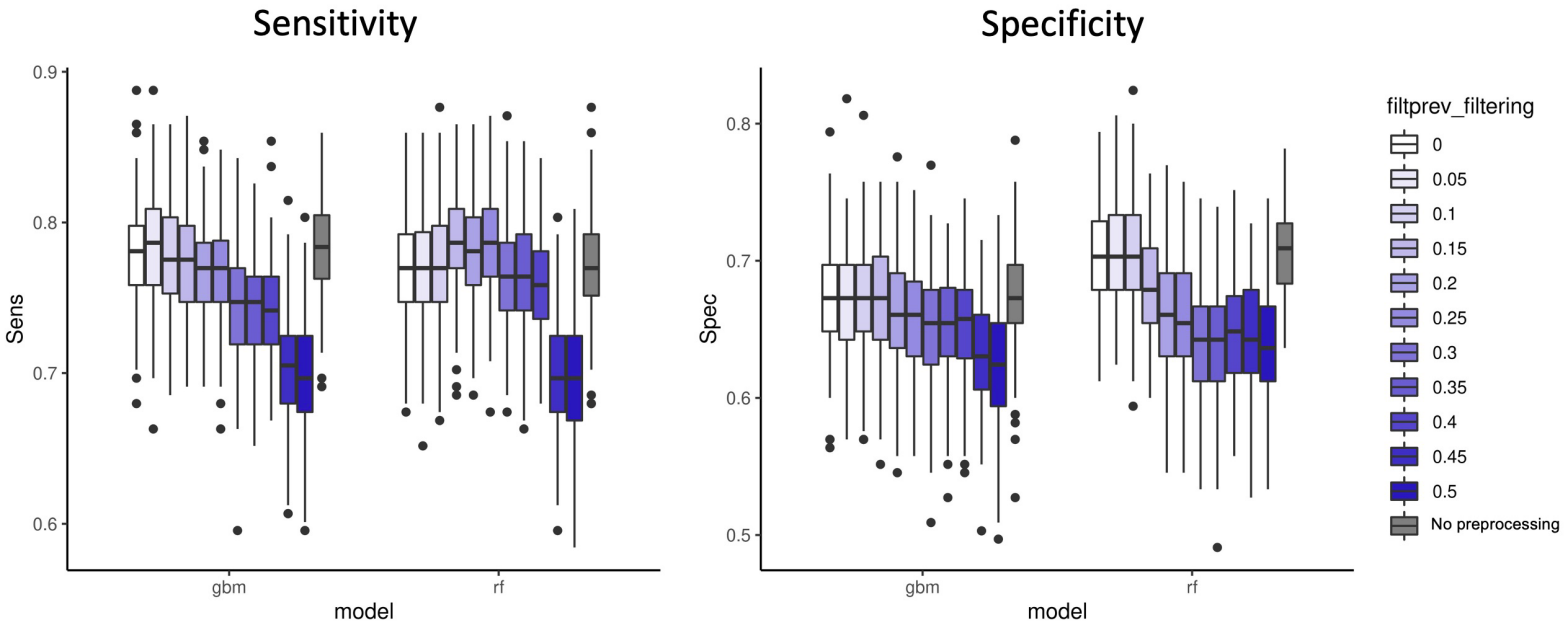
Sensitivity and specificity of the two best performing ML models (GBM and RF) on 100 data split repetitions applied on the CRC dataset with a range of filter on prevalence (shades of blue) or no filter on prevalence (gray).

We first applied a fixed threshold on abundance values (retained features with a total abundance across samples >5e-06). A variable threshold of prevalence across samples in [0–0.5], with 0.05 steps was applied to remove features with low prevalence. Figure 2 shows the sensitivity and specificity results for the two best performing models: GBM and RF. We observed that a small filtering slightly improved the performances both on accuracy and computing resources criteria. However, it is noteworthy that no filtering on prevalence at all is also a valid option in terms of performances. As expected, strong filtering on prevalence (>0.15–0.2) decreases the sensitivity for GBM and the specificity for RF. Additional analyses of other microbiome datasets (Supplementary material 1 and Supplementary Figure S1), showed that performance was not affected by 0.2 prevalence filter with regard to 0 prevalence filter in RF models. However, other models such as PLS-DA got better classification error rates when 0.2 prevalence filter was applied. The results from these additional datasets indicate that the effect on performance of the low-abundance filter depends both on the ML model applied and on the characteristics of each dataset, being the level of sparsity of the database a key factor to consider. All in all, this fact highlights the importance of including the low-abundance filter as another hyperparameter to tune while training the model by cross-validation strategies.

**Figure 3:**
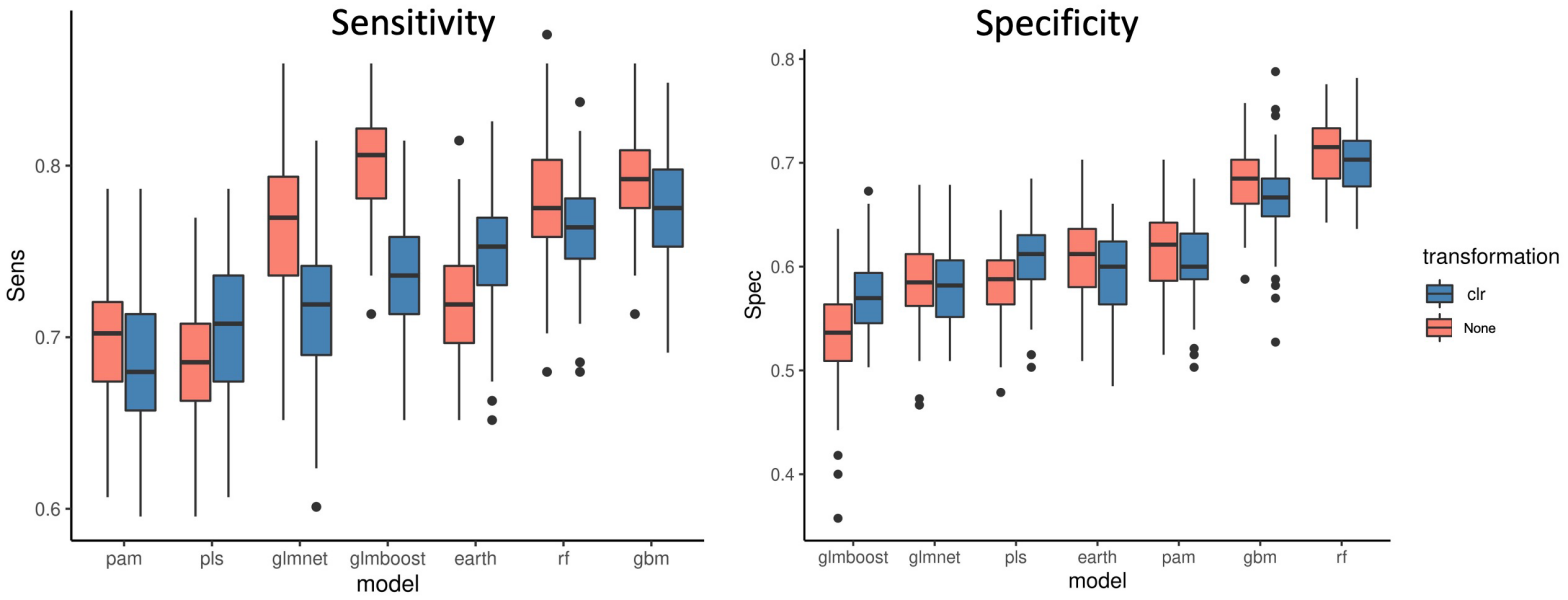
Sensitivity and specificity of 7 ML models across 100 data split repetitions applied on the CRC dataset with a CLR logratio transformation before (blue) or no transformation (red).

Figure 3 shows the sensitivity and specificity results for all the models with or without the CLR logratio transformation before the modeling process. We observed that for the majority of the models, the CLR transformation decreased the sensitivity of the models, and it was particularly striking for the Glmnet and Glmboost models. It only improved the sensitivity for the PLS and Earth models. It improved the specificity of the PLS and Glmboost models, nevertheless, RF and GBM remained the top performing models. In contrast, results obtained from other datasets (Supplementary material 1 and Supplementary Figure S1) pointed out that, in general, CLR transformation rendered a better performance when compared to TSS normalization followed by logarithmic transformation. Again, RF was less affected by the type of normalization applied than PLS-DA. Taken together, as with threshold filtering, it is important to cross-validate transformation options in order to enhance the predictive performance and extract the best modeling pipeline.

### 3.3. Evaluation of feature selection and predictive modeling

To evaluate the performance of different ML pipelines we used the JADBio automl approach. JADBio is specifically designed for biomedical data and is able to fully automate the production of customizable ML pipelines that simultaneously integrate preprocessing, feature selection and predictive modeling algorithms (Table 4). Specifically, for preprocessing, we performed standardization. For feature selection we evaluated LASSO regularized regression (Tibshirani, 1996) and the Statistical Equivalent Signatures (SES) algorithm (Lagani et al., 2017). Both methods can handle the high-dimensionality of microbiome data. Regarding sample size and expected signature size, LASSO tends to perform better when sample sizes are larger but returns a greater number of features. SES, on the other hand, drawing inspiration from causal modeling theory, demonstrates better performance at low sample sizes and leads to smaller feature subsets at the expense of predictive performance. SES also produces multiple signatures that exhibit statistically indistinguishable predictive performances. For modeling, we employed well known linear/interpretable algorithms such as ridge regression (Hoerl and Kennard, 1970) and decision trees (Breiman, 2017) as well as non-linear ones such as random forests (Breiman, 2001) and SVMs (Chang and Lin, 2011). To find the best model, we employed grid search and the Generalized Cross-validation approach, while the BBC-CV algorithm was used to correct for testing multiple ML pipelines. More details about the architecture can be found in Tsamardinos et al. (2022).

**Table 4:** Algorithms used for comparative evaluation of ML pipelines.

Algorithm	Used for
Standardization	Preprocessing
LASSO	(Single) feature selection
SES	(Multiple) feature selection
Decision trees	Predictive modeling
Ridge regression	Predictive modeling
Random forests	Predictive modeling
Support vector machines	Predictive modeling
Generalized cross-validation	Performance estimation
Grid search with heuristics	Configuration space search
BBC-CV	Performance correction

**Figure 4:**
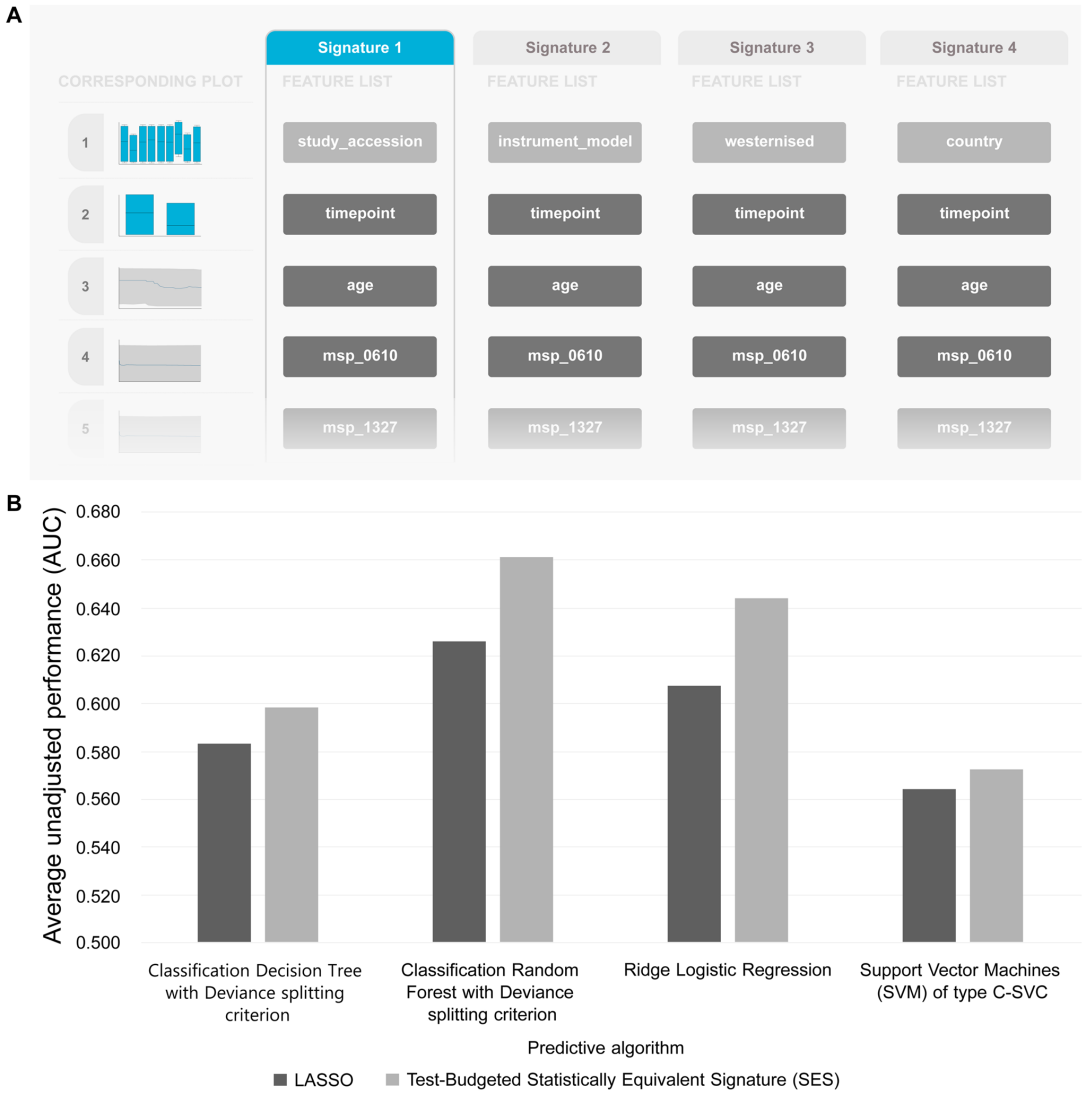
**(A)** Features detected by feature selection that generate classification bias. **(B)** Comparative evaluation of tested pipelines.

Table 5 summarizes the results from analyzing the collected shotgun data. Initially, our aim was to uncover any classification bias from technical or demographic covariates, through feature selection. Indeed, when we employed JADBio on the complete, all-cohorts, dataset we observed that the cohort ID/country exhibited predictive power, indicating inherent variations in gut species between different countries (Figure 4A). Furthermore, we found that the timepoint of measurement, the instrument model, and the westernization status of samples also possessed predictive value, pointing toward the existence of batch effects (the full description of the metadata features used in the models are provided in Supplementary Table S1).

**Table 5:** Summarized results from the analysis of the collected CRC shotgun datasets with 2014 features using JADBio.

Cohort	Samples	Training AUC	Validation AUC	Feature selection	Predictive algorithm	Report link	Features
Austrian	109	0.90		SES	RF	Report 1	msp_0041, msp_0610, msp_1600, msp_1721, msp_0304,msp_0376, msp_0831, msp_0417, msp_0869, msp_1017, msp_0350, msp_1101^a^, msp_0717, msp_0215, msp_1176^b^, msp_1587^a^, msp_1195^b^
French	114	0.79		SES	RF	Report 2	msp_0024, msp_0554, msp_0006, msp_1158, msp_1327, msp_0610, msp_0800, msp_0168, msp_1402, msp_0350, msp_0835, msp_0317^a^, msp_1,193, msp_0541^b^, msp_1037, msp_1060c^a^, msp_1213^b^
Chinese	128	0.74		SES	RF	Report 3	age, msp_0033^a^, msp_0990, msp_1028c^b^, msp_0468, msp_0044, msp_0457, msp_0713, msp_0235, msp_0178, msp_1206, msp_0236, msp_0318, msp_0126, msp_0542, msp_0639, msp_0864, msp_1603c, msp_0154, msp_1901^a^, msp_1193^b^
Italian	113	0.63		SES	RF	Report 4	msp_1234, msp_0258, msp_0100, msp_0275, msp_1489c, msp_0562, msp_0199^a^, msp_0338, msp_0340, msp_0125^b^, msp_0369^aa^, msp_0215^b^, msp_0906^b^
Indian	140	1.00		LASSO	RF	Report 5	study_accession, age, msp_0027, msp_0128, msp_0258, msp_0585, msp_0841, msp_1459
German	125	0.98		SES	RF	Report 6	HQ_clean_read_count^a^, msp_1234, msp_0722, instrument_model, msp_0610, msp_1018, msp_1428, mapped_read_count^a^
USA	104	0.64		SES	SVM	Report 7	msp_0147, msp_1293, msp_1522, msp_0679^a^, msp_0035, msp_1,193, msp_0766, msp_0747, msp_1,038, msp_0083, msp_1850, msp_0566, msp_0180, msp_1069^b^, msp_1621, msp_1241, msp_0845, msp_0854^a^, msp_1110^b^
Japanese	577	0.69		SES	RF	Report 8	timepoint, msp_1327, msp_0003, msp_0749, msp_1315, msp_0132, msp_0935, msp_0436, msp_0574c, msp_0468, msp_0152, msp_0126, msp_1276, msp_1049, msp_1004, msp_1156, msp_0887, msp_0323, msp_0525, msp_0118, msp_1590c, msp_1028c, msp_0635, msp_0062, msp_0610
All cohorts	1,410	0.85		SES	RF	Report 9	study_accession^a^, timepoint, age, msp_0610, msp_1327, msp_1112, msp_0454, msp_0668, msp_0910, msp_0129, msp_0128, msp_1,193, msp_0305, msp_0054, msp_0757, msp_0100, msp_1028c, msp_1682c, msp_0357, msp_1172, msp_0032, msp_0297, msp_0105, msp_1158, msp_0389, msp_0935, msp_1173c, msp_1946, msp_0546, msp_1234, msp_0574c, msp_0468, msp_0110, msp_0833, msp_0484, msp_1790, msp_1188, msp_0172, msp_0864, msp_1600, msp_0853c, msp_0831, msp_0258, msp_0077, msp_0126, msp_0062, msp_1156, msp_0204, msp_0034, msp_0542, instrument_model^a^, westernised^a^, country^a^
Revised data (best perf.)	1,117	0.77	0.758	SES	RF	Report 10	msp_1081, gender, msp_0578, msp_0622, msp_0833, msp_0610, msp_0100, msp_1579c, msp_0676, msp_0236, msp_1010, msp_0317, msp_0757, msp_0910, msp_0496, msp_0574c, msp_1327, msp_1028c, msp_0938, msp_0126, msp_0129, msp_1188, msp_0172, msp_1069, msp_0257, msp_0835, msp_1324, msp_1682c, msp_0864, msp_1102, msp_1467, msp_1245, msp_0668, msp_1158, msp_0305, msp_0937, msp_1671c, msp_1790, msp_0110, msp_1754, msp_0062, msp_0814, msp_0853c, msp_1322, msp_1217, msp_1156, msp_1036, msp_0805, msp_1712, msp_1231, msp_0454, msp_0935, msp_1657, msp_1234, msp_0076, msp_1487, msp_1570, msp_1042, msp_0118, msp_1112, msp_0457, msp_1048, msp_0232, msp_0542, msp_0468, msp_0258, msp_1789, msp_1173c, msp_0347, msp_0089
Revised data (best inter.)	1,117	0.75	0.73	SES	LR	Report 11	msp_0100, msp_0118, msp_0126, msp_0129, msp_0172, msp_0257, msp_0258, msp_0317, msp_0468, msp_0542, msp_0574c, msp_0610, msp_0676, msp_0805, msp_0833, msp_0835, msp_0910, msp_0935, msp_0937, msp_1028c, msp_1112, msp_1156, msp_1158, msp_1188, msp_1231, msp_1245, msp_1327, msp_1570, msp_1754, msp_1789

Full description of the metadata and species features can be found in Supplementary Tables S1, S2. The variable health_status was set as the outcome to be predicted with classification (binary) as the analysis type. Samples from the same country were merged together. Detailed signatures, including the lists of selected features, can be accessed via the links to the respective JADBio analysis reports. Superscript letters denote statistical equivalence of features, i.e., replacing one feature with another feature labeled with the same superscript letter will, on average, yield the same predictive performance. RF, Random Forest; SVM, Support Vector Machine; LR, Logistic Regression.

To address these variabilities, we conducted a series of subsequent analyses by splitting the data into the different cohorts. Feature selection identified that the timepoint feature was predictive in the Japanese cohort, the instrument model in the German cohort and the westernization status in the Indian cohort (Table 5). Therefore, we repeated the analysis on the entire sample set after excluding the problematic samples coming from these cohorts. This time, the revised data we divided into two parts: one for training and the other for testing. The revised findings indicated the absence of latent variabilities, suggesting that our modifications successfully controlled for the previously observed effects.

**Figure 5:**
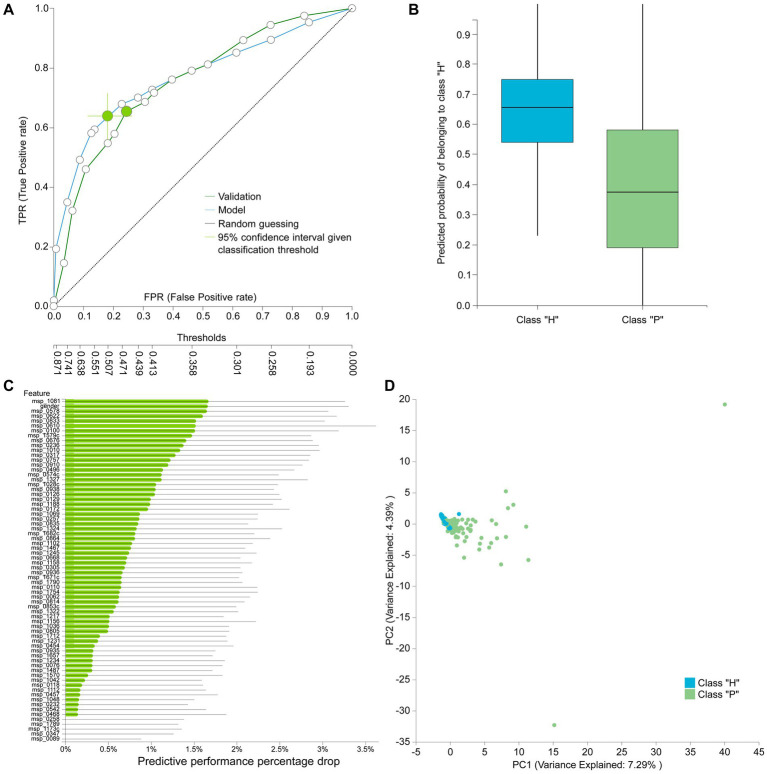
**(A)** Train (blue) and test (green) AUCROC after analyzing the revised dataset. ROC Curve considering CRC patients (P) as the positive class. **(B)** Out-of-sample (training) predictions for Healthy (H) and CRC patients class (P). **(C)** Feature importance defined as the percentage drop in predictive performance when the feature is removed from the model. Gray lines indicate 95% confidence intervals. **(D)** Supervised PCA on the selected features depicts the model performance in separating the two classes and also outlier samples.

The best performing pipeline on the revised data was a combination of SES and random forests, consistent with the majority of separate cohort analyses. A total of 596 different pipelines were evaluated by a repeated 10-fold CV approach (see Supplementary report for details). As shown in Figure 4B, pipelines incorporating SES for feature selection demonstrated higher average performance during training than those with LASSO. Among the predictive modeling algorithms tested, Random Forests exhibited the highest predictive performance, followed by Ridge Logistic Regression. Figure 5A illustrates the ROC curves of the best performing model. The achieved performance in terms of AUC on the test data was 0.758; on par with the training performance of 0.777 (C.I. [0.708, 0.822]). Figure 5B also presents the out-of-sample predictions during training.

In terms of feature selection, the best performing pipeline resulted in a signature comprising 70 features, primarily consisting of microbiome species, with the addition of gender. Figure 5C illustrates the importance of these features in predicting the outcome (see Supplementary Table S2 for the corresponding species names). While SES and RFs demonstrated superior performance in most of the analyses, Table 5 reveals the significant variation in predictive performances and generated signatures that was found. The detailed taxonomy of the species involved is provided in Supplementary Table S3. Variability in performance was also highlighted by Wirbel et al. (2019) where only the predictive performance on several cohorts was examined. This suggests the need for further investigation into the specificity of these microbiome signatures. Interestingly, however, 20 species present in the revised dataset’s signature were also found in the signatures generated when analyzing each cohort independently, indicating their potential importance across diverse geographic communities.

Among the selected species in the revised dataset’s signature, their relevance is in agreement with previous reports in the literature regarding their predictive role in CRC. In particular, considering the top five most important species identified for the revised dataset, excluding gender (Figure 5C), *Fusobacterium gonidiaformans* (msp_1081) was detected in colorectal carcinoma relative to normal colon (Castellarin et al., 2012; Kostic et al., 2012), and found to be enriched in adenomas (Gevers et al., 2014). Several Clostridium species (msp_0578) have been associated with CRC (i.e., *Clostridium symbiosum*, *Clostridium hylemonae*, and *Clostridium scindens*) (Zeller et al., 2014). In addition, an increased risk of CRC was found in patients with bacteremia from *Clostridium septicum*, *Clostridium perfringens* or other species, such as *Fusobacterium nucleatum* and Peptostreptococcus species (Kwong et al., 2018). Christensenellales (msp_0622) has shown to be associated with both host genetic status CRC and risk (Waters and Ley, 2019), while *Streptococcus thermophilus* (msp_0833) has been identified to be depleted in patients with colorectal cancer (Qing et al., 2021). Regarding *Fusobacterium nucleatum* subspecies animalis (msp_0610), also selected when independently analyzing the Austrian, French, German and Japanese cohorts, *Fusobacterium nucleatum* was associated with stages of colorectal neoplasia development, colorectal cancer and disease outcome (Flanagan et al., 2014).

Figure 5D visualizes how well the selected features separate the two classes on a low dimensional space representation. Furthermore, it indicates a few samples that could be considered as outliers and would need further investigation.

**Figure 6:**
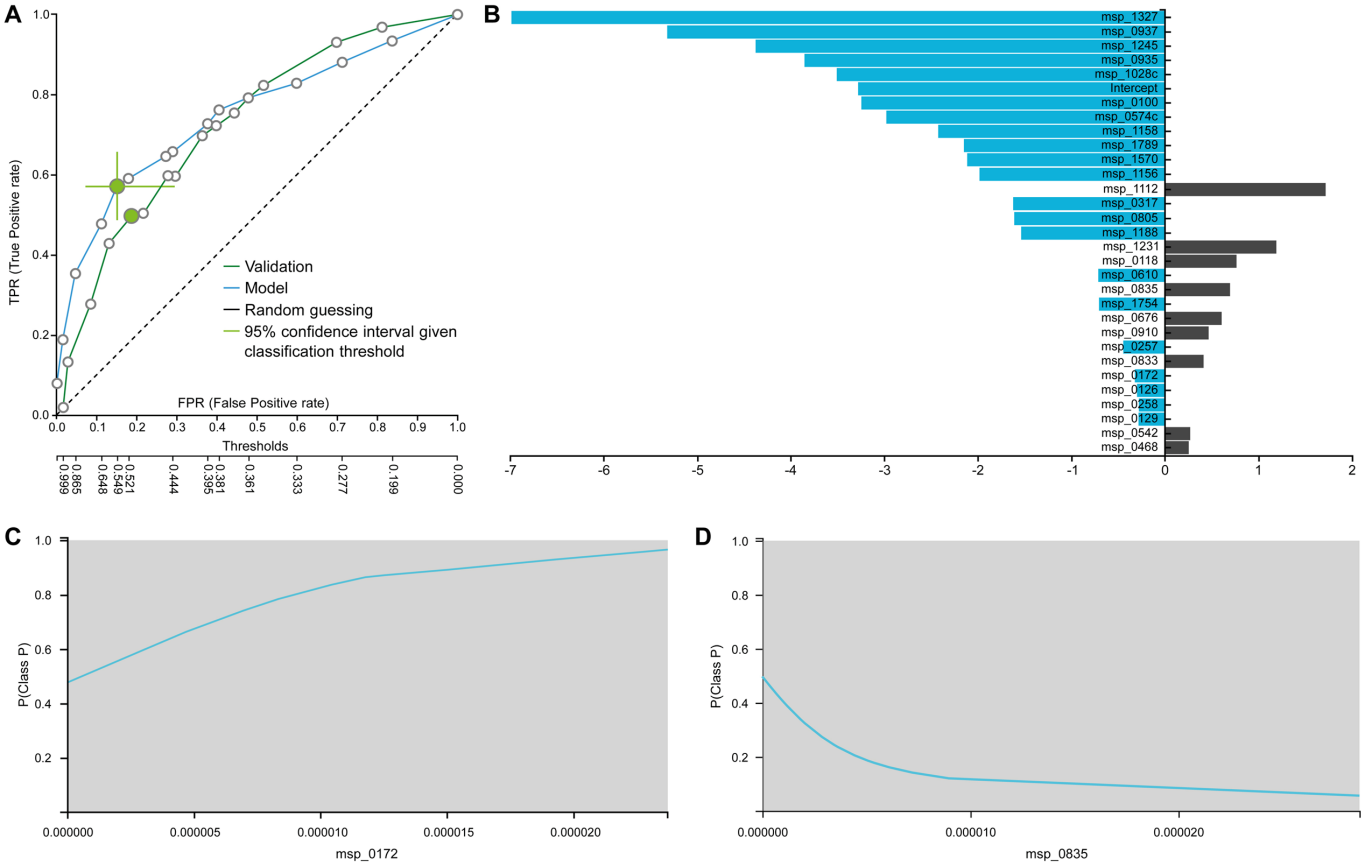
**(A)** ROC of the best interpretable model. **(B)** Contribution of each species to the prediction from logistic regression as the best interpretable model. Feature Interpretation using ICE plots with an example of a **(C)** risk factor (the higher the abundance, the higher the probability to be in the P (Patients) class) and a **(D)** protective factor (the higher the abundance, the lower the probability to be in the P (Patients) class).

Figure 6A displays the ROC curves of the best interpretable model on both training and test sets. This model, based on Ridge Regression, demonstrates performance that is comparable to the best-performing model (training AUC 0.754, C.I. [0.693, 0.811], test AUC 0.731). The linear nature of the predictive algorithm enables direct interpretation of the generated model. In Figure 6B, the species selected by the interpretable model are showcased alongside their corresponding linear coefficient values in the log-odd formula.

For instance, the identified association of *Peptostreptococcus stomatis* (msp_1327) corroborates findings from the French cohort’s original data publication (Zeller et al., 2014). Furthermore, while msp_0937 corresponds to an unclassified Duodenibacillus species, it is noteworthy that Duodenibacillus massiliensis is linked with treatment response for patients with rectal cancer (Jang et al., 2020). Similarly, concerning the unknown msp_1245 (Parvimonas species), *Parvimonas micra* together with *Fusobacterium nucleatum* (msp_0574c), *Peptostreptococcus stomatis* (msp_1327), and *Akkermansia muciniphila* were found to be over-represented in CRC patients compared to non-CRC controls (Osman et al., 2021). In another confirmatory study *Peptostreptococcus anaerobius* (msp_0935) has been implicated in modulating colorectal carcinogenesis and tumor immunity. Additionally, *Prevotella intermedia* (msp_1028) and *Fusobacterium nucleatum* (msp_0574c) were found to act synergistically, enhancing the migration and invasion of CRC cells (Long et al., 2019; Lo et al., 2022).

The sign of the coefficient indicates whether the species is considered a risk factor or not by the model. For instance, Ruthenibacterium lactatiformans (msp_0172) has been previously identified as putative candidate non-invasive biomarkers in CRC patients (Trivieri et al., 2020). Figure 6C illustrates how its abundance influences the prediction. Specifically, the greater the abundance, the more the risk for a sample to be classified as a patient case (P). In contrast, species Clostridiales bacterium (msp_0835) is found to have a protective effect against CRC, as evidenced by its ICE plot (Figure 6D). The higher its abundance, the lower the probability to be in the patient class. Indeed, a recent study demonstrated the effectiveness of this species in both prophylactic and therapeutic contexts speculating its applicability to primary prevention in patient populations with a strong genetic predisposition or family history of CRC (Montalban-Arques et al., 2021). Taken together, combining feature selection results with interpretable modeling and visualization techniques, meaningful conclusions can be drawn about the predictive significance of different species.

## 4. Discussion

Our objectives in this work have been to: (1) review the challenges for an analyst when performing predictive modeling of microbiome data, (2) create a comprehensive set of practical advices, and (3) explore opportunities for automating various aspects of ML analysis to construct pipelines suitable for clinicians and non-experts in translational applications. To achieve these goals, we considered a typical ML workflow that starts after microbiome-related profiles are organized in a two-dimensional table format, such as OTUs, ASV, or MSP (metagenomic species) tables. This process involves multiple steps, including data preprocessing (e.g., normalization, filtering), feature selection, predictive modeling, and performance estimation. Our objective was to address the challenges associated with each of these steps considering diverse algorithms, their combinations, as well as our capacity to interpret and explain their results. Through the utilization of benchmark dataset(s) and automated machine learning techniques (AutoML), we were able to derive several noteworthy conclusions regarding the optimal utilization of ML methods toward disease diagnosis, prognosis, and biomarker discovery.

In the context of data preprocessing, a major challenge lies in selecting the appropriate normalization and filtering approaches due to variations in sampling depth, data sparsity (represented by an excess of zeros in the tables), and data compositionality. To mitigate sampling variability, rarefaction is used to remove samples. However, this may decrease statistical power and does not address compositionality (McMurdie and Holmes, 2014). Alternatively, researchers incorporate the sampled variation as covariates in data analysis. On the other hand, sparsity hampers models that rely on Gaussian assumptions. Certain algorithms, like decision trees and random forests, can handle sparsity, while others may fail. Filtering rare features and removing near-zero variance ones is a successful strategy, outperforming imputation methods in the context of logarithmic transformations that can introduce aberrant observations and depend on imputation algorithm quality. Finally, regarding normalization, contemporary sequencing cannot capture the total number of bacterial species, only their proportions. Compositional analysis is the appropriate mathematical framework, but its application and impact on ML models are still actively researched (Greenacre et al., 2021; Hron et al., 2021). From our observations, the CLR transformation seems to be useful for the PLS regression, although it was not in the top performing models. For the other models, the CLR transformation globally decreased the performances. However, these observations are based on the specific data set used in our experiments, and further evaluation will be necessary to assess their generalizability to other data sets before providing general recommendations regarding the choice of transformations.

For feature selection and predictive modeling, the primary challenges revolve around the high dimensionality of the data and the complex interactions inherent to microbial species, including co-occurrence and partial correlation. Building models that incorporate the thousands of microbiome features in a multivariate manner while maintaining predictive performance with limited sample sizes is undeniably demanding. It requires the utilization of scalable methods that account for the intricate dependency structure of microbiome data, as well as appropriate performance estimation protocols to generate an optimal final model. Neglecting these considerations can result in overestimated conclusions and misleading insights. Using the JADBio autoML approach our observations indicate that multivariate feature selection methods such as the Statistically Equivalent Signatures algorithm combined with Random Forests can yield optimal balance between performance and results interpretability and explainability. These suggest a good starting point for an analyst.

However, it must be acknowledged that no single ML pipeline can universally accommodate all predictive modeling scenarios. As demonstrated here, there are several algorithms that account for the biological, methodological, and technical challenges in microbiome data. Additionally, different ML methods with different strengths and limitations exist for addressing the dimensionality and complexity of the problem and the underlying patterns in the data. Therefore, a highly advisable approach is to explore a diverse range of methods at each stage of the ML pipeline, and communicate the results according to the open science principles to facilitate transparency, verification, and reuse. Then, only through rigorous performance evaluation can the optimal predictive model and biomarkers be effectively identified, specifically tailored to address the particular microbiome problem at hand.

## Author contributions

GP: Conceptualization, Formal analysis, Project administration, Supervision, Visualization, Writing – original draft, Writing – review & editing. SoT: Conceptualization, Formal analysis, Project administration, Supervision, Visualization, Writing – original draft, Writing – review & editing. ML: Writing – review & editing, Methodology. TK: Writing – review & editing, Visualization. EI: Writing – review & editing, Methodology. JE: Writing – review & editing, Methodology. PN: Writing – original draft. ATo: Writing – review & editing. AS: Writing – review & editing. RS: Writing – review & editing. SB: Data curation, Writing – original draft. GV: Data curation, Writing – original draft. SaT: Writing – original draft. LL: Writing – review & editing. ATe: Writing – review & editing. MC: Funding acquisition, Writing – review & editing. MB: Conceptualization, Formal analysis, Project administration, Supervision, Visualization, Writing – original draft, Writing – review & editing.

## Funding

The author(s) declare financial support was received for the research, authorship, and/or publication of this article. This study was based upon work from COST Action ML4Microbiome “Statistical and machine learning techniques in human microbiome studies” (CA18131), supported by COST (European Cooperation in Science and Technology, www.cost.eu). MB acknowledged support through the Metagenopolis grant ANR-11-DPBS-0001. ML acknowledged support by FCT - Fundação para a Ciência e a Tecnologia, I.P., with references UIDB/00297/2020 and UIDP/00297/2020 (NOVA Math), UIDB/00667/2020 and UIDP/00667/2020 (UNIDEMI), and CEECINST/00042/2021.

## Conflict of interest

GP was directly affiliated with JADBio—Gnosis DA, S.A., which offers the JADBio service commercially.

The remaining authors declare that the research was conducted in the absence of any commercial or financial relationships that could be construed as a potential conflict of interest.

## Publisher’s note

All claims expressed in this article are solely those of the authors and do not necessarily represent those of their affiliated organizations, or those of the publisher, the editors and the reviewers. Any product that may be evaluated in this article, or claim that may be made by its manufacturer, is not guaranteed or endorsed by the publisher.

## Footnotes

^1^ https://www.ml4microbiome.eu/
