# Supplementary material for: Machine learning approaches in microbiome research: challenges and best practices
Source: Front Microbiol. 2023 Sep 22;14:1261889. doi: 10.3389/fmicb.2023.1261889 (PMC10556866; doi:10.3389/fmicb.2023.1261889)
Supplement: Supplementary file 1 [file Data_Sheet_1.PDF]

JADBio Description of Performed Analysis

Visit analysis

Setup

JADBio version **1.4.105** ran on dataset **CRC_no_t2_IND_GER_TRAIN** with **782** samples and **2008** features to create a predictive model for outcome named **health_status**. The outcome was discrete leading to a **classification** modeling.

The preferences of the analysis were set to **true** for feature selection and **false** for full feature models tried.
The **AUC** metric was used to optimize for the best model.
The maximum number of features to select was set to **100**.
The effort to spend on tuning the algorithms were set to **Normal**.
The number of CPU cores to use for the analysis was set to **2**.
The execution time was **00:12:31**.

Configuration Space

JADBio's AI decide to try the following algorithms and tuning hyper-parameter values:

Algorithm Type	Algorithm	Hyper-parameter	Set of Values
Preprocessing	Mean Imputation		
	Mode Imputation		
	Constant Removal		
	Variable Normalization		
Feature Selection	LASSO	penalty	1.0, 1.5, 0.5
	Test-Budgeted Statistically Equivalent Signature (SES)	alpha	0.05, 0.01
		maxK	3.0, 2.0
Modeling	Classification Random Forest with Deviance splitting criterion	minLeafSize	4.0, 2.0, 3.0
		nTrees	500, 100
	Ridge Logistic Regression	lambda	0.1, 10.0, 1.0
	Support Vector Machines (SVM) of type C-SVC with Polynomial Kernel	cost	1.0, 0.1, 0.01, 10.0
		degree	2, 3
		gamma	0.1, 1.0, 0.01, 10.0
	Classification Decision Tree with Deviance splitting criterion	minLeafSize	3, 2, 4
		alpha	0.05, 0.01
	Support Vector Machines (SVM) of type C-SVC with Gaussian Kernel	cost	10.0, 1.0, 0.1, 0.01
		gamma	10.0, 0.1, 1.0, 0.01
	Support Vector Machines (SVM) of type C-SVC with Linear Kernel	cost	1.0, 0.1, 10.0, 0.01

Leading to **596** combinations and corresponding configurations (machine learning pipelines) to try. For the full configurations tested see the Appendix.

Configuration Estimation Protocol

JADBio's AI system decided to estimate the out-of-sample performance of the models produced by each configuration using **Incomplete 10-fold CV without dropping.** Overall, 4172 models were set out to train.

A detailed report of the above is available at Visit analysis

JADBio Results Summary

Overview

A result summary is presented for analysis optimized for Interpretability. The model is produced by applying the algorithms in sequence (configuration) on the training data:

Preprocessing	Feature Selection	Predictive algorithm
Mean Imputation, Mode Imputation, Constant Removal, Standardization	Test-Budgeted Statistically Equivalent Signature (SES) algorithm with hyper-parameters: maxK = 2, alpha = 0.01 and budget = 3 * nvars	Ridge Logistic Regression with penalty hyper-parameter lambda = 0.1

The **Area Under The Curve** is **0.754** with 95% confidence interval being [**0.693,0.811**].
The **Mean Average Precision (a.k.a. Average Area Under the Precision-Recall curve)** is **0.762** with 95% confidence interval being [**0.708,0.816**].

The Area Under the ROC Curve is shown in the figure below:

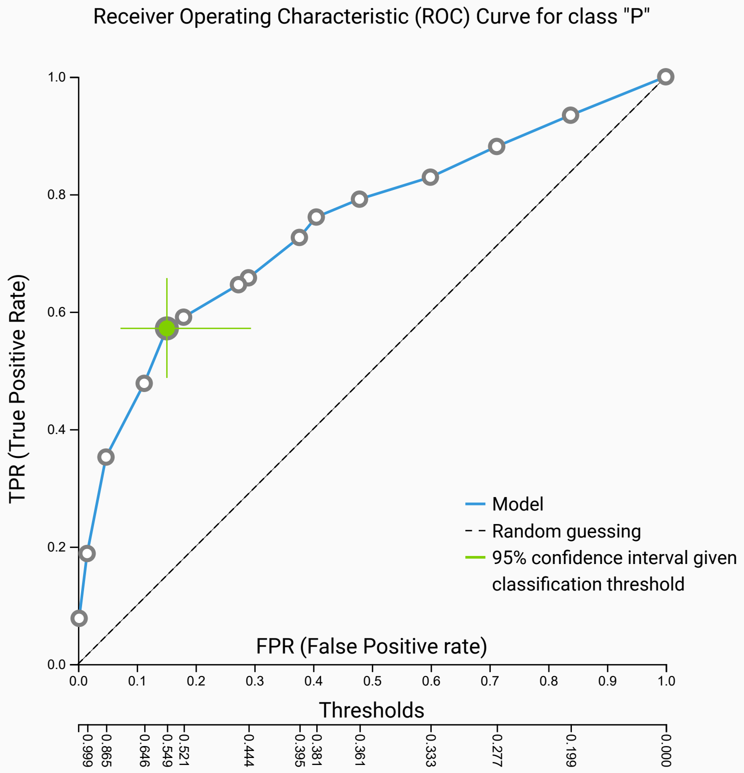

Selecting to classify as class: P any sample with predicted probability to be in this class above **0.5490**, the model achieves:

Metric	Mean estimate	CI
Accuracy	0.712	[0.644, 0.767]
Balanced Accuracy	0.710	[0.643, 0.763]
F1 Score	0.655	[0.582, 0.719]
Matthews correlation criterion (phi coefficient)	0.441	[0.295, 0.551]
Precision	0.794	[0.655, 0.895]
True Positive Rate (a.k.a. Sensitivity, Recall. Hit Rate)	0.572	[0.487, 0.657]
Specificity	0.849	[0.709, 0.928]
True Positive Ratio	0.282	[0.231, 0.338]
True Negative Ratio	0.430	[0.351, 0.500]
False Positive Ratio	0.077	[0.036, 0.151]
False Negative Ratio	0.211	[0.165, 0.259]

Feature Selection

There were **30** features selected out of the **2008** available.

The selected features consist of the following subset called a signature. **There was a single signature identified.** The first signature identified by the system is the set: **msp_1327, msp_1158, msp_0317, msp_1156, msp_0935, msp_0129, msp_0172, msp_0468, msp_1028c, msp_0258, msp_0126, msp_0542, msp_1570, msp_0937, msp_0574c, msp_0835, msp_1789, msp_0610, msp_0805, msp_0100, msp_0833, msp_1112, msp_0676, msp_1231, msp_1754, msp_1188, msp_0910, msp_1245, msp_0257, msp_0118** in order of importance. The following features cannot be substituted with others and still obtain an equal predictive performance: **msp_1327, msp_1158, msp_0317, msp_1156, msp_0935, msp_0129, msp_0172, msp_0468, msp_1028c, msp_0258, msp_0126, msp_0542, msp_1570, msp_0937, msp_0574c, msp_0835, msp_1789, msp_0610, msp_0805, msp_0100, msp_0833, msp_1112, msp_0676, msp_1231, msp_1754, msp_1188, msp_0910, msp_1245, msp_0257, msp_0118**.

The performance achieved by adding each feature in sequence to the model relative to the performance of the final model with all selected features is shown below. The features are added in order of importance:

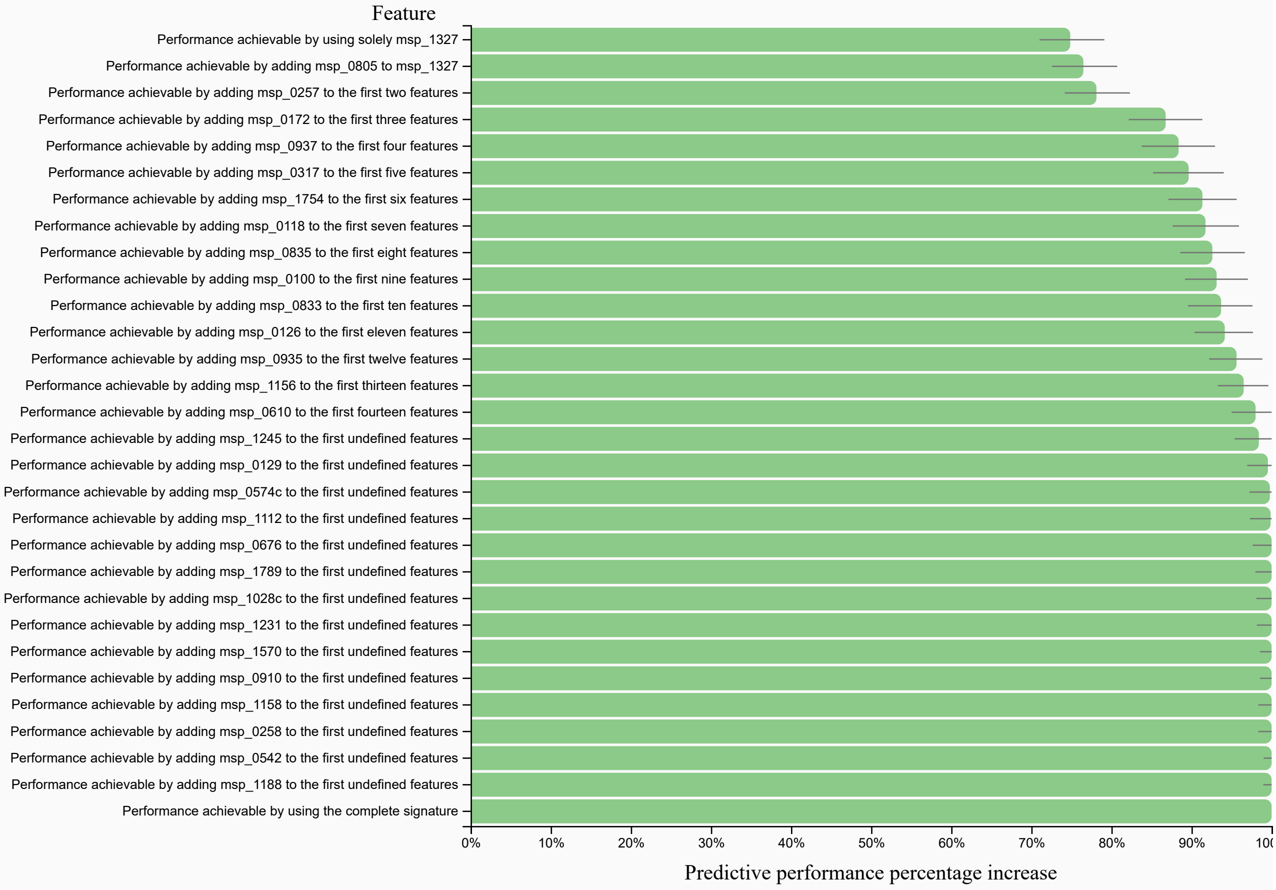

Some features may not seem to add predictive performance to the model; however, the feature selection algorithms include them as an effort to make the final model more robust to noise. The performances achieved by a model that contains all features except one, relative to the performance achieved when the feature is removed is shown below:

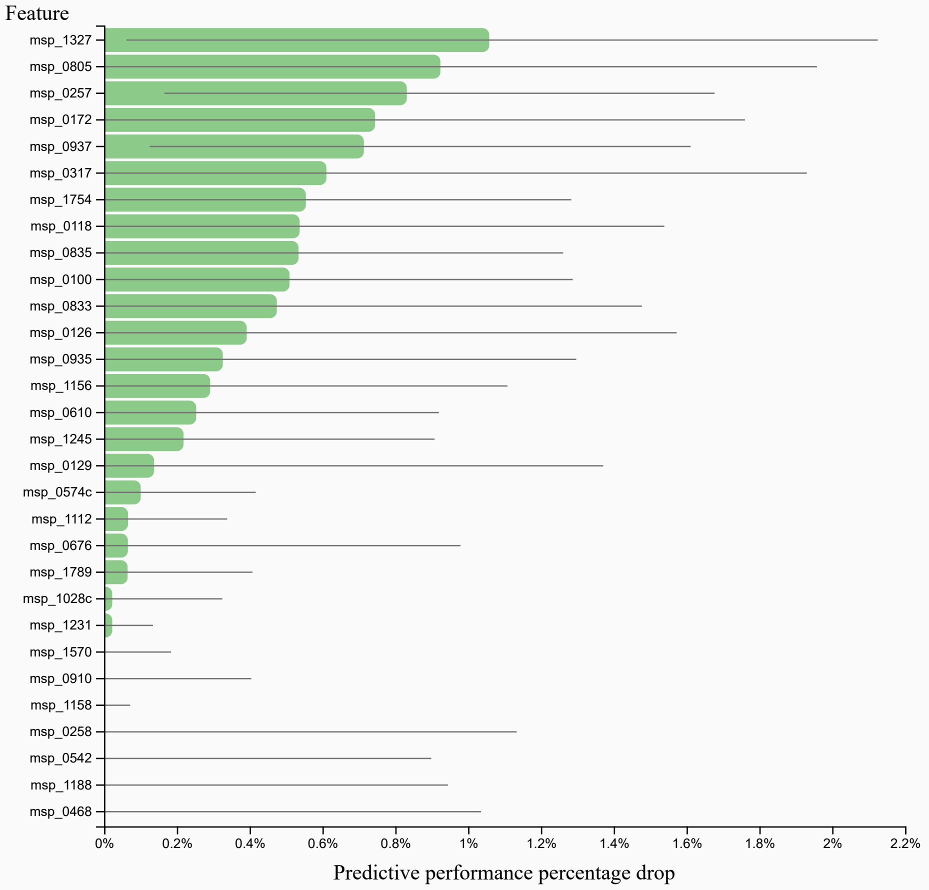

For some features there is no noticeable drop in performance when they are removed because they carry predictive information that is shared by other features selected.

The separation of the predictions of the classes achieved by the model is shown in the box-plots below. These are the out-of-sample predictions made by model produced by the same configuration as the final model when the sample was used for testing (e.g.., during cross-validation) and was not used to train the model.

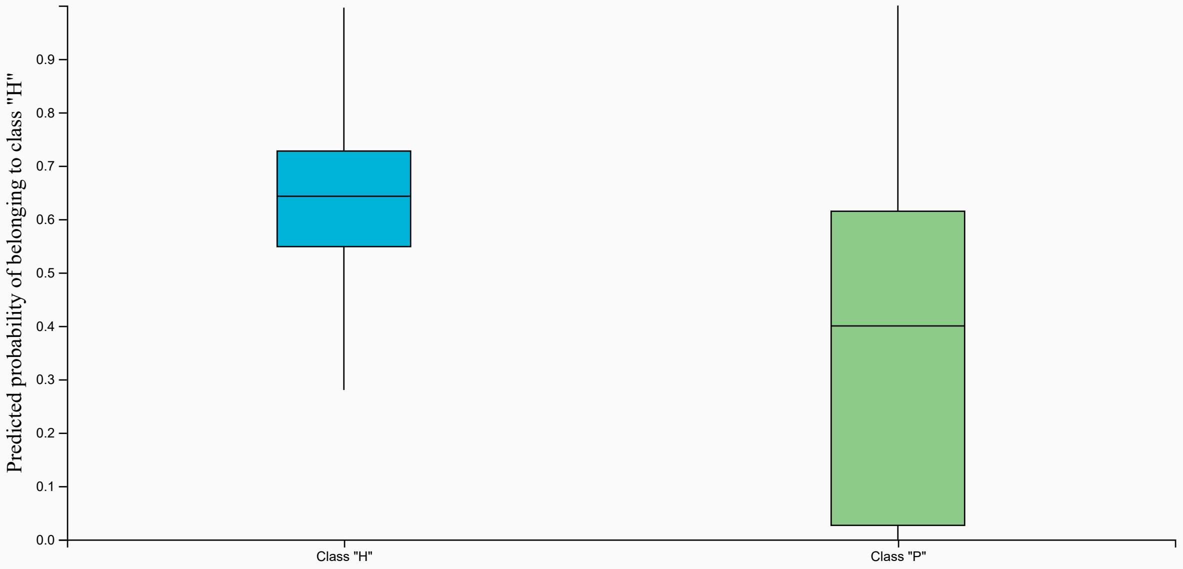

Appendix

Configuration	Preprocessing	Name	Hyperparams	Name	Hyperparams	Performance (unadjusted)	Time (miliseconds)	Dropped
1	Mean Imputation, Mode Imputation, Constant Removal, Standardization	LASSO	penalty = 1.0	Classification Random Forest with Deviance splitting criterion	ntrees = 500, minimum leaf size = 4	0.6320572140410197	00:00:04.4764	false
2	Mean Imputation, Mode Imputation, Constant Removal, Standardization	LASSO	penalty = 1.5	Classification Random Forest with Deviance splitting criterion	ntrees = 100, minimum leaf size = 4	0.5	00:00:00.000	false
3	Mean Imputation, Mode Imputation, Constant Removal, Standardization	Test-Budgeted Statistically Equivalent Signature (SES)	maxK = 3, alpha = 0.05, budget = 3 * nvars	Classification Random Forest with Deviance splitting criterion	ntrees = 500, minimum leaf size = 4	0.5445959573489938	00:00:38.38791	false
4	Mean Imputation, Mode Imputation, Constant Removal, Standardization	Test-Budgeted Statistically Equivalent Signature (SES)	maxK = 3, alpha = 0.05, budget = 3 * nvars	Ridge Logistic Regression	lambda = 0.1	0.5507683471499262	00:00:38.38723	false
5	Mean Imputation, Mode Imputation, Constant Removal, Standardization	Test-Budgeted Statistically Equivalent Signature (SES)	maxK = 3, alpha = 0.05, budget = 3 * nvars	Support Vector Machines (SVM) of type C-SVC	kernel = 'Polynomial Kernel', cost = 1.0, gamma = 0.1, degree = 2	0.4894239111091337	00:00:38.38726	false
6	Mean Imputation, Mode Imputation, Constant Removal, Standardization	LASSO	penalty = 1.0	Classification Decision Tree with Deviance splitting criterion	minimum leaf size = 3, alpha = 0.05	0.5763152024538665	00:00:04.4615	false
7	Mean Imputation, Mode Imputation, Constant Removal, Standardization	Test-Budgeted Statistically Equivalent Signature (SES)	maxK = 2, alpha = 0.05, budget = 3 * nvars	Support Vector Machines (SVM) of type C-SVC	kernel = 'Polynomial Kernel', cost = 0.1, gamma = 1.0, degree = 3	0.588100305003139	00:00:18.18096	false
8	Mean Imputation, Mode Imputation, Constant Removal, Standardization	Test-Budgeted Statistically Equivalent Signature (SES)	maxK = 3, alpha = 0.01, budget = 3 * nvars	Support Vector Machines (SVM) of type C-SVC	kernel = 'Polynomial Kernel', cost = 0.1, gamma = 1.0, degree = 3	0.5	00:00:03.3038	false
9	Mean Imputation, Mode Imputation, Constant Removal, Standardization	LASSO	penalty = 1.0	Support Vector Machines (SVM) of type C-SVC	kernel = 'Polynomial Kernel', cost = 0.1, gamma = 0.01, degree = 2	0.5677759178518287	00:00:04.4613	false
10	Mean Imputation,	LASSO	penalty = 0.5	Support Vector	kernel = 'Polynomial	0.5517178671530898	00:00:00.005	false

Configuration	Preprocessing	Name	Hyperparams	Name	Hyperparams	Performance (unadjusted)	Time (miliseconds)	Dropped
	Mode Imputation, Constant Removal, Standardization			Machines (SVM) of type C-SVC	Kernel', cost = 0.01, gamma = 10.0, degree = 2			
11	Mean Imputation, Mode Imputation, Constant Removal, Standardization	Test-Budgeted Statistically Equivalent Signature (SES)	maxK = 3, alpha = 0.01, budget = 3 * nvars	Support Vector Machines (SVM) of type C-SVC	kernel = 'Polynomial Kernel', cost = 0.01, gamma = 1.0, degree = 2	0.5334744961516218	00:00:03.3028	false
12	Mean Imputation, Mode Imputation, Constant Removal, Standardization	LASSO	penalty = 1.5	Support Vector Machines (SVM) of type C-SVC	kernel = 'Gaussian Kernel', cost = 10.0, gamma = 10.0	0.5	00:00:00.000	false
13	Mean Imputation, Mode Imputation, Constant Removal, Standardization	LASSO	penalty = 0.5	Classification Random Forest with Deviance splitting criterion	ntrees = 100, minimum leaf size = 2	0.7433589521140128	00:00:00.056	false
14	Mean Imputation, Mode Imputation, Constant Removal, Standardization	Test-Budgeted Statistically Equivalent Signature (SES)	maxK = 2, alpha = 0.01, budget = 3 * nvars	Support Vector Machines (SVM) of type C-SVC	kernel = 'Polynomial Kernel', cost = 10.0, gamma = 10.0, degree = 2	0.573161639305364	00:00:00.780	false
15	Mean Imputation, Mode Imputation, Constant Removal, Standardization	LASSO	penalty = 1.5	Support Vector Machines (SVM) of type C-SVC	kernel = 'Gaussian Kernel', cost = 1.0, gamma = 0.1	0.5	00:00:00.000	false
16	Mean Imputation, Mode Imputation, Constant Removal, Standardization	Test-Budgeted Statistically Equivalent Signature (SES)	maxK = 2, alpha = 0.05, budget = 3 * nvars	Support Vector Machines (SVM) of type C-SVC	kernel = 'Polynomial Kernel', cost = 1.0, gamma = 0.01, degree = 3	0.6534200392499987	00:00:18.18096	false
17	Mean Imputation, Mode Imputation, Constant Removal, Standardization	LASSO	penalty = 1.0	Support Vector Machines (SVM) of type C-SVC	kernel = 'Gaussian Kernel', cost = 10.0, gamma = 0.1	0.5818818804901801	00:00:04.4620	false
18	Mean Imputation, Mode Imputation, Constant Removal, Standardization	Test-Budgeted Statistically Equivalent Signature (SES)	maxK = 3, alpha = 0.01, budget = 3 * nvars	Support Vector Machines (SVM) of type C-SVC	kernel = 'Polynomial Kernel', cost = 1.0, gamma = 0.01, degree = 3	0.5264297022141151	00:00:03.3032	false
19	Mean Imputation, Mode Imputation,	Test-Budgeted Statistically Equivalent	maxK = 2, alpha = 0.05, budget = 3 * nvars	Support Vector Machines	kernel = 'Polynomial Kernel', cost = 0.01, gamma	0.5939933660581433	00:00:18.18095	false

Configuration	Preprocessing	Name	Hyperparams	Name	Hyperparams	Performance (unadjusted)	Time (miliseconds)	Dropped
	Constant Removal, Standardization	Signature (SES)		(SVM) of type C-SVC	= 1.0, degree = 2			
20	Mean Imputation, Mode Imputation, Constant Removal, Standardization	LASSO	penalty = 0.5	Support Vector Machines (SVM) of type C-SVC	kernel = 'Polynomial Kernel', cost = 1.0, gamma = 0.1, degree = 3	0.6669577358089503	00:00:00.011	false
21	Mean Imputation, Mode Imputation, Constant Removal, Standardization	LASSO	penalty = 1.0	Support Vector Machines (SVM) of type C-SVC	kernel = 'Polynomial Kernel', cost = 1.0, gamma = 1.0, degree = 2	0.5722968046348614	00:00:04.4613	false
22	Mean Imputation, Mode Imputation, Constant Removal, Standardization	LASSO	penalty = 1.0	Support Vector Machines (SVM) of type C-SVC	kernel = 'Gaussian Kernel', cost = 0.1, gamma = 10.0	0.5665726508084807	00:00:04.4620	false
23	Mean Imputation, Mode Imputation, Constant Removal, Standardization	LASSO	penalty = 1.5	Classification Random Forest with Deviance splitting criterion	ntrees = 100, minimum leaf size = 2	0.5	00:00:00.000	false
24	Mean Imputation, Mode Imputation, Constant Removal, Standardization	LASSO	penalty = 0.5	Classification Decision Tree with Deviance splitting criterion	minimum leaf size = 2, alpha = 0.05	0.6211494898488825	00:00:00.019	false
25	Mean Imputation, Mode Imputation, Constant Removal, Standardization	Test-Budgeted Statistically Equivalent Signature (SES)	maxK = 3, alpha = 0.05, budget = 3 * nvars	Support Vector Machines (SVM) of type C-SVC	kernel = 'Polynomial Kernel', cost = 0.1, gamma = 0.01, degree = 3	0.5019984935217728	00:00:38.38726	false
26	Mean Imputation, Mode Imputation, Constant Removal, Standardization	Test-Budgeted Statistically Equivalent Signature (SES)	maxK = 3, alpha = 0.05, budget = 3 * nvars	Classification Random Forest with Deviance splitting criterion	ntrees = 500, minimum leaf size = 4	0.5524058234837586	00:00:38.38754	false
27	Mean Imputation, Mode Imputation, Constant Removal, Standardization	Test-Budgeted Statistically Equivalent Signature (SES)	maxK = 2, alpha = 0.05, budget = 3 * nvars	Support Vector Machines (SVM) of type C-SVC	kernel = 'Polynomial Kernel', cost = 10.0, gamma = 0.01, degree = 3	0.6534200392499987	00:00:18.18096	false
28	Mean Imputation, Mode Imputation, Constant	LASSO	penalty = 0.5	Support Vector Machines (SVM) of type C-SVC	kernel = 'Polynomial Kernel', cost = 0.01, gamma = 0.01, degree = 2	0.5398777392198445	00:00:00.005	false

Configuration	Preprocessing	Name	Hyperparams	Name	Hyperparams	Performance (unadjusted)	Time (miliseconds)	Dropped
	Removal, Standardization							
29	Mean Imputation, Mode Imputation, Constant Removal, Standardization	LASSO	penalty = 1.5	Classification Random Forest with Deviance splitting criterion	ntrees = 500, minimum leaf size = 2	0.5	00:00:00.000	false
30	Mean Imputation, Mode Imputation, Constant Removal, Standardization	LASSO	penalty = 0.5	Support Vector Machines (SVM) of type C-SVC	kernel = 'Gaussian Kernel', cost = 10.0, gamma = 1.0	0.6516653443272876	00:00:00.020	false
31	Mean Imputation, Mode Imputation, Constant Removal, Standardization	LASSO	penalty = 0.5	Classification Random Forest with Deviance splitting criterion	ntrees = 100, minimum leaf size = 3	0.7447451839658317	00:00:00.054	false
32	Mean Imputation, Mode Imputation, Constant Removal, Standardization	Test-Budgeted Statistically Equivalent Signature (SES)	maxK = 2, alpha = 0.05, budget = 3 * nvars	Support Vector Machines (SVM) of type C-SVC	kernel = 'Polynomial Kernel', cost = 1.0, gamma = 10.0, degree = 2	0.5743604573564088	00:00:18.18096	false
33	Mean Imputation, Mode Imputation, Constant Removal, Standardization	Test-Budgeted Statistically Equivalent Signature (SES)	maxK = 2, alpha = 0.01, budget = 3 * nvars	Support Vector Machines (SVM) of type C-SVC	kernel = 'Linear Kernel', cost = 1.0	0.7184075820715496	00:00:00.823	false
34	Mean Imputation, Mode Imputation, Constant Removal, Standardization	Test-Budgeted Statistically Equivalent Signature (SES)	maxK = 2, alpha = 0.01, budget = 3 * nvars	Support Vector Machines (SVM) of type C-SVC	kernel = 'Polynomial Kernel', cost = 1.0, gamma = 1.0, degree = 3	0.5773926309857484	00:00:00.784	false
35	Mean Imputation, Mode Imputation, Constant Removal, Standardization	Test-Budgeted Statistically Equivalent Signature (SES)	maxK = 3, alpha = 0.05, budget = 3 * nvars	Support Vector Machines (SVM) of type C-SVC	kernel = 'Linear Kernel', cost = 0.1	0.5498299187317406	00:00:38.38727	false
36	Mean Imputation, Mode Imputation, Constant Removal, Standardization	Test-Budgeted Statistically Equivalent Signature (SES)	maxK = 2, alpha = 0.01, budget = 3 * nvars	Support Vector Machines (SVM) of type C-SVC	kernel = 'Gaussian Kernel', cost = 0.01, gamma = 0.01	0.722106795588577	00:00:00.788	false
37	Mean Imputation, Mode Imputation, Constant Removal, Standardization	Test-Budgeted Statistically Equivalent Signature (SES)	maxK = 2, alpha = 0.05, budget = 3 * nvars	Support Vector Machines (SVM) of type C-SVC	kernel = 'Gaussian Kernel', cost = 0.01, gamma = 10.0	0.5	00:00:18.18106	false

Configuration	Preprocessing	Name	Hyperparams	Name	Hyperparams	Performance (unadjusted)	Time (miliseconds)	Dropped
38	Mean Imputation, Mode Imputation, Constant Removal, Standardization	Test-Budgeted Statistically Equivalent Signature (SES)	maxK = 2, alpha = 0.01, budget = 3 * nvars	Classification Random Forest with Deviance splitting criterion	ntrees = 100, minimum leaf size = 2	0.7706789162254749	00:00:00.839	false
39	Mean Imputation, Mode Imputation, Constant Removal, Standardization	Test-Budgeted Statistically Equivalent Signature (SES)	maxK = 2, alpha = 0.05, budget = 3 * nvars	Classification Random Forest with Deviance splitting criterion	ntrees = 500, minimum leaf size = 2	0.77809316189883	00:00:18.18519	false
40	Mean Imputation, Mode Imputation, Constant Removal, Standardization	Test-Budgeted Statistically Equivalent Signature (SES)	maxK = 3, alpha = 0.01, budget = 3 * nvars	Classification Random Forest with Deviance splitting criterion	ntrees = 100, minimum leaf size = 4	0.5515673429134967	00:00:03.3030	false
41	Mean Imputation, Mode Imputation, Constant Removal, Standardization	Test-Budgeted Statistically Equivalent Signature (SES)	maxK = 2, alpha = 0.01, budget = 3 * nvars	Support Vector Machines (SVM) of type C-SVC	kernel = 'Polynomial Kernel', cost = 10.0, gamma = 0.01, degree = 2	0.512263152951412	00:00:00.784	false
42	Mean Imputation, Mode Imputation, Constant Removal, Standardization	Test-Budgeted Statistically Equivalent Signature (SES)	maxK = 2, alpha = 0.05, budget = 3 * nvars	Support Vector Machines (SVM) of type C-SVC	kernel = 'Polynomial Kernel', cost = 10.0, gamma = 1.0, degree = 3	0.588100305003139	00:00:18.18097	false
43	Mean Imputation, Mode Imputation, Constant Removal, Standardization	Test-Budgeted Statistically Equivalent Signature (SES)	maxK = 3, alpha = 0.01, budget = 3 * nvars	Support Vector Machines (SVM) of type C-SVC	kernel = 'Polynomial Kernel', cost = 10.0, gamma = 1.0, degree = 3	0.5	00:00:03.3034	false
44	Mean Imputation, Mode Imputation, Constant Removal, Standardization	LASSO	penalty = 1.0	Support Vector Machines (SVM) of type C-SVC	kernel = 'Linear Kernel', cost = 10.0	0.6149250097630665	00:00:04.4732	false
45	Mean Imputation, Mode Imputation, Constant Removal, Standardization	Test-Budgeted Statistically Equivalent Signature (SES)	maxK = 3, alpha = 0.01, budget = 3 * nvars	Support Vector Machines (SVM) of type C-SVC	kernel = 'Polynomial Kernel', cost = 10.0, gamma = 0.01, degree = 3	0.5264297022141151	00:00:03.3033	false
46	Mean Imputation, Mode Imputation, Constant Removal, Standardization	Test-Budgeted Statistically Equivalent Signature (SES)	maxK = 3, alpha = 0.01, budget = 3 * nvars	Support Vector Machines (SVM) of type C-SVC	kernel = 'Linear Kernel', cost = 0.1	0.5471102868117037	00:00:03.3030	false
47	Mean Imputation,	Test-Budgeted	maxK = 2, alpha = 0.01,	Support Vector	kernel = 'Linear Kernel',	0.7196746797961373	00:00:01.1308	false

Configuration	Preprocessing	Name	Hyperparams	Name	Hyperparams	Performance (unadjusted)	Time (miliseconds)	Dropped
	Mode Imputation, Constant Removal, Standardization	Statistically Equivalent Signature (SES)	budget = 3 * nvars	Machines (SVM) of type C-SVC	cost = 10.0			
48	Mean Imputation, Mode Imputation, Constant Removal, Standardization	LASSO	penalty = 1.5	Classification Random Forest with Deviance splitting criterion	ntrees = 500, minimum leaf size = 3	0.5	00:00:00.000	false
49	Mean Imputation, Mode Imputation, Constant Removal, Standardization	Test-Budgeted Statistically Equivalent Signature (SES)	maxK = 3, alpha = 0.01, budget = 3 * nvars	Support Vector Machines (SVM) of type C-SVC	kernel = 'Linear Kernel', cost = 1.0	0.5521361342211545	00:00:03.3032	false
50	Mean Imputation, Mode Imputation, Constant Removal, Standardization	Test-Budgeted Statistically Equivalent Signature (SES)	maxK = 3, alpha = 0.05, budget = 3 * nvars	Support Vector Machines (SVM) of type C-SVC	kernel = 'Gaussian Kernel', cost = 0.01, gamma = 0.1	0.5604277582516449	00:00:38.38734	false
51	Mean Imputation, Mode Imputation, Constant Removal, Standardization	LASSO	penalty = 0.5	Support Vector Machines (SVM) of type C-SVC	kernel = 'Polynomial Kernel', cost = 0.01, gamma = 1.0, degree = 3	0.6020204604212701	00:00:00.005	false
52	Mean Imputation, Mode Imputation, Constant Removal, Standardization	LASSO	penalty = 0.5	Classification Decision Tree with Deviance splitting criterion	minimum leaf size = 4, alpha = 0.01	0.687233153149145	00:00:00.015	false
53	Mean Imputation, Mode Imputation, Constant Removal, Standardization	LASSO	penalty = 1.0	Support Vector Machines (SVM) of type C-SVC	kernel = 'Polynomial Kernel', cost = 10.0, gamma = 0.01, degree = 2	0.5	00:00:04.4613	false
54	Mean Imputation, Mode Imputation, Constant Removal, Standardization	Test-Budgeted Statistically Equivalent Signature (SES)	maxK = 2, alpha = 0.05, budget = 3 * nvars	Support Vector Machines (SVM) of type C-SVC	kernel = 'Gaussian Kernel', cost = 0.01, gamma = 0.01	0.6624855407750145	00:00:18.18104	false
55	Mean Imputation, Mode Imputation, Constant Removal, Standardization	Test-Budgeted Statistically Equivalent Signature (SES)	maxK = 3, alpha = 0.05, budget = 3 * nvars	Classification Random Forest with Deviance splitting criterion	ntrees = 500, minimum leaf size = 2	0.5375148299743442	00:00:38.38809	false
56	Mean Imputation, Mode Imputation,	LASSO	penalty = 0.5	Support Vector Machines	kernel = 'Gaussian Kernel', cost =	0.65225947017445	00:00:00.018	false

Configuration	Preprocessing	Name	Hyperparams	Name	Hyperparams	Performance (unadjusted)	Time (miliseconds)	Dropped
	Constant Removal, Standardization			(SVM) of type C-SVC	1.0, gamma = 1.0			
57	Mean Imputation, Mode Imputation, Constant Removal, Standardization	Test-Budgeted Statistically Equivalent Signature (SES)	maxK = 3, alpha = 0.05, budget = 3 * nvars	Support Vector Machines (SVM) of type C-SVC	kernel = 'Gaussian Kernel', cost = 1.0, gamma = 1.0	0.5221352197060699	00:00:38.38735	false
58	Mean Imputation, Mode Imputation, Constant Removal, Standardization	Test-Budgeted Statistically Equivalent Signature (SES)	maxK = 3, alpha = 0.01, budget = 3 * nvars	Support Vector Machines (SVM) of type C-SVC	kernel = 'Polynomial Kernel', cost = 1.0, gamma = 0.1, degree = 2	0.527929352473887	00:00:03.3028	false
59	Mean Imputation, Mode Imputation, Constant Removal, Standardization	LASSO	penalty = 0.5	Support Vector Machines (SVM) of type C-SVC	kernel = 'Gaussian Kernel', cost = 0.1, gamma = 1.0	0.6173012968812565	00:00:00.017	false
60	Mean Imputation, Mode Imputation, Constant Removal, Standardization	Test-Budgeted Statistically Equivalent Signature (SES)	maxK = 2, alpha = 0.01, budget = 3 * nvars	Support Vector Machines (SVM) of type C-SVC	kernel = 'Polynomial Kernel', cost = 1.0, gamma = 10.0, degree = 2	0.573161639305364	00:00:00.784	false
61	Mean Imputation, Mode Imputation, Constant Removal, Standardization	Test-Budgeted Statistically Equivalent Signature (SES)	maxK = 3, alpha = 0.05, budget = 3 * nvars	Support Vector Machines (SVM) of type C-SVC	kernel = 'Polynomial Kernel', cost = 10.0, gamma = 0.1, degree = 2	0.4894239111091337	00:00:38.38725	false
62	Mean Imputation, Mode Imputation, Constant Removal, Standardization	LASSO	penalty = 0.5	Classification Random Forest with Deviance splitting criterion	ntrees = 500, minimum leaf size = 4	0.743349868754727	00:00:00.342	false
63	Mean Imputation, Mode Imputation, Constant Removal, Standardization	Test-Budgeted Statistically Equivalent Signature (SES)	maxK = 3, alpha = 0.05, budget = 3 * nvars	Support Vector Machines (SVM) of type C-SVC	kernel = 'Polynomial Kernel', cost = 10.0, gamma = 10.0, degree = 3	0.5	00:00:38.38726	false
64	Mean Imputation, Mode Imputation, Constant Removal, Standardization	LASSO	penalty = 1.5	Support Vector Machines (SVM) of type C-SVC	kernel = 'Gaussian Kernel', cost = 10.0, gamma = 0.01	0.5	00:00:00.000	false
65	Mean Imputation, Mode Imputation, Constant	Test-Budgeted Statistically Equivalent Signature (SES)	maxK = 3, alpha = 0.01, budget = 3 * nvars	Classification Random Forest with Deviance splitting criterion	ntrees = 500, minimum leaf size = 4	0.5497310831318928	00:00:03.3052	false

Configuration	Preprocessing	Name	Hyperparams	Name	Hyperparams	Performance (unadjusted)	Time (miliseconds)	Dropped
	Removal, Standardization							
66	Mean Imputation, Mode Imputation, Constant Removal, Standardization	LASSO	penalty = 0.5	Classification Decision Tree with Deviance splitting criterion	minimum leaf size = 4, alpha = 0.05	0.6571009933611147	00:00:00.015	false
67	Mean Imputation, Mode Imputation, Constant Removal, Standardization	LASSO	penalty = 1.5	Support Vector Machines (SVM) of type C-SVC	kernel = 'Polynomial Kernel', cost = 0.01, gamma = 0.01, degree = 3	0.5	00:00:00.000	false
68	Mean Imputation, Mode Imputation, Constant Removal, Standardization	Test-Budgeted Statistically Equivalent Signature (SES)	maxK = 3, alpha = 0.05, budget = 3 * nvars	Support Vector Machines (SVM) of type C-SVC	kernel = 'Polynomial Kernel', cost = 0.1, gamma = 10.0, degree = 2	0.5	00:00:38.38729	false
69	Mean Imputation, Mode Imputation, Constant Removal, Standardization	Test-Budgeted Statistically Equivalent Signature (SES)	maxK = 3, alpha = 0.01, budget = 3 * nvars	Support Vector Machines (SVM) of type C-SVC	kernel = 'Gaussian Kernel', cost = 0.01, gamma = 10.0	0.5146388839455642	00:00:03.3046	false
70	Mean Imputation, Mode Imputation, Constant Removal, Standardization	Test-Budgeted Statistically Equivalent Signature (SES)	maxK = 2, alpha = 0.05, budget = 3 * nvars	Classification Random Forest with Deviance splitting criterion	ntrees = 100, minimum leaf size = 4	0.7723272060822669	00:00:18.18147	false
71	Mean Imputation, Mode Imputation, Constant Removal, Standardization	Test-Budgeted Statistically Equivalent Signature (SES)	maxK = 3, alpha = 0.05, budget = 3 * nvars	Classification Random Forest with Deviance splitting criterion	ntrees = 500, minimum leaf size = 2	0.5471207913768642	00:00:38.38759	false
72	Mean Imputation, Mode Imputation, Constant Removal, Standardization	Test-Budgeted Statistically Equivalent Signature (SES)	maxK = 2, alpha = 0.05, budget = 3 * nvars	Support Vector Machines (SVM) of type C-SVC	kernel = 'Polynomial Kernel', cost = 10.0, gamma = 10.0, degree = 2	0.5743604573564088	00:00:18.18095	false
73	Mean Imputation, Mode Imputation, Constant Removal, Standardization	Test-Budgeted Statistically Equivalent Signature (SES)	maxK = 2, alpha = 0.05, budget = 3 * nvars	Support Vector Machines (SVM) of type C-SVC	kernel = 'Polynomial Kernel', cost = 0.1, gamma = 0.01, degree = 3	0.6526435974057432	00:00:18.18100	false
74	Mean Imputation, Mode Imputation, Constant Removal, Standardization	Test-Budgeted Statistically Equivalent Signature (SES)	maxK = 3, alpha = 0.05, budget = 3 * nvars	Support Vector Machines (SVM) of type C-SVC	kernel = 'Polynomial Kernel', cost = 0.01, gamma = 1.0, degree = 2	0.5013681578205871	00:00:38.38729	false

Configuration	Preprocessing	Name	Hyperparams	Name	Hyperparams	Performance (unadjusted)	Time (miliseconds)	Dropped
75	Mean Imputation, Mode Imputation, Constant Removal, Standardization	LASSO	penalty = 0.5	Classification Random Forest with Deviance splitting criterion	ntrees = 100, minimum leaf size = 4	0.7414411274735162	00:00:00.053	false
76	Mean Imputation, Mode Imputation, Constant Removal, Standardization	Test-Budgeted Statistically Equivalent Signature (SES)	maxK = 3, alpha = 0.05, budget = 3 * nvars	Support Vector Machines (SVM) of type C-SVC	kernel = 'Polynomial Kernel', cost = 1.0, gamma = 0.1, degree = 3	0.5	00:00:38.38725	false
77	Mean Imputation, Mode Imputation, Constant Removal, Standardization	LASSO	penalty = 1.5	Classification Random Forest with Deviance splitting criterion	ntrees = 500, minimum leaf size = 3	0.5	00:00:00.000	false
78	Mean Imputation, Mode Imputation, Constant Removal, Standardization	LASSO	penalty = 1.0	Support Vector Machines (SVM) of type C-SVC	kernel = 'Polynomial Kernel', cost = 1.0, gamma = 0.01, degree = 2	0.5	00:00:04.4613	false
79	Mean Imputation, Mode Imputation, Constant Removal, Standardization	Test-Budgeted Statistically Equivalent Signature (SES)	maxK = 2, alpha = 0.05, budget = 3 * nvars	Support Vector Machines (SVM) of type C-SVC	kernel = 'Polynomial Kernel', cost = 10.0, gamma = 0.1, degree = 2	0.6330795553973693	00:00:18.18100	false
80	Mean Imputation, Mode Imputation, Constant Removal, Standardization	LASSO	penalty = 1.0	Support Vector Machines (SVM) of type C-SVC	kernel = 'Gaussian Kernel', cost = 0.01, gamma = 1.0	0.5934230299614914	00:00:04.4616	false
81	Mean Imputation, Mode Imputation, Constant Removal, Standardization	Test-Budgeted Statistically Equivalent Signature (SES)	maxK = 2, alpha = 0.01, budget = 3 * nvars	Classification Random Forest with Deviance splitting criterion	ntrees = 500, minimum leaf size = 4	0.7776799617386662	00:00:01.1027	false
82	Mean Imputation, Mode Imputation, Constant Removal, Standardization	LASSO	penalty = 1.0	Support Vector Machines (SVM) of type C-SVC	kernel = 'Gaussian Kernel', cost = 1.0, gamma = 10.0	0.5777902596728507	00:00:04.4622	false
83	Mean Imputation, Mode Imputation, Constant Removal, Standardization	Test-Budgeted Statistically Equivalent Signature (SES)	maxK = 2, alpha = 0.01, budget = 3 * nvars	Classification Decision Tree with Deviance splitting criterion	minimum leaf size = 3, alpha = 0.05	0.6387868092321534	00:00:00.790	false
84	Mean Imputation,	Test-Budgeted	maxK = 3, alpha = 0.05,	Classification Random	ntrees = 100, minimum leaf	0.5364381429411793	00:00:38.38733	false

Configuration	Preprocessing	Name	Hyperparams	Name	Hyperparams	Performance (unadjusted)	Time (miliseconds)	Dropped
	Mode Imputation, Constant Removal, Standardization	Statistically Equivalent Signature (SES)	budget = 3 * nvars	Forest with Deviance splitting criterion	size = 4			
85	Mean Imputation, Mode Imputation, Constant Removal, Standardization	LASSO	penalty = 1.0	Support Vector Machines (SVM) of type C-SVC	kernel = 'Polynomial Kernel', cost = 1.0, gamma = 1.0, degree = 3	0.5751495355746368	00:00:04.4615	false
86	Mean Imputation, Mode Imputation, Constant Removal, Standardization	Test-Budgeted Statistically Equivalent Signature (SES)	maxK = 3, alpha = 0.01, budget = 3 * nvars	Support Vector Machines (SVM) of type C-SVC	kernel = 'Polynomial Kernel', cost = 1.0, gamma = 1.0, degree = 3	0.5	00:00:03.3034	false
87	Mean Imputation, Mode Imputation, Constant Removal, Standardization	Test-Budgeted Statistically Equivalent Signature (SES)	maxK = 3, alpha = 0.01, budget = 3 * nvars	Classification Random Forest with Deviance splitting criterion	ntrees = 500, minimum leaf size = 2	0.5507383473476591	00:00:03.3055	false
88	Mean Imputation, Mode Imputation, Constant Removal, Standardization	Test-Budgeted Statistically Equivalent Signature (SES)	maxK = 3, alpha = 0.01, budget = 3 * nvars	Support Vector Machines (SVM) of type C-SVC	kernel = 'Polynomial Kernel', cost = 10.0, gamma = 0.01, degree = 2	0.5370217951189611	00:00:03.3028	false
89	Mean Imputation, Mode Imputation, Constant Removal, Standardization	Test-Budgeted Statistically Equivalent Signature (SES)	maxK = 3, alpha = 0.05, budget = 3 * nvars	Support Vector Machines (SVM) of type C-SVC	kernel = 'Polynomial Kernel', cost = 0.01, gamma = 0.01, degree = 2	0.5140527910011715	00:00:38.38726	false
90	Mean Imputation, Mode Imputation, Constant Removal, Standardization	LASSO	penalty = 1.5	Support Vector Machines (SVM) of type C-SVC	kernel = 'Polynomial Kernel', cost = 0.1, gamma = 10.0, degree = 3	0.5	00:00:00.000	false
91	Mean Imputation, Mode Imputation, Constant Removal, Standardization	Test-Budgeted Statistically Equivalent Signature (SES)	maxK = 2, alpha = 0.05, budget = 3 * nvars	Classification Random Forest with Deviance splitting criterion	ntrees = 500, minimum leaf size = 4	0.7730451622152027	00:00:18.18453	false
92	Mean Imputation, Mode Imputation, Constant Removal, Standardization	LASSO	penalty = 1.0	Support Vector Machines (SVM) of type C-SVC	kernel = 'Polynomial Kernel', cost = 10.0, gamma = 10.0, degree = 2	0.5432774490466799	00:00:04.4608	false
93	Mean Imputation, Mode Imputation,	LASSO	penalty = 0.5	Ridge Logistic Regression	lambda = 10.0	0.7021442289154841	00:00:00.002	false

Configuration	Preprocessing	Name	Hyperparams	Name	Hyperparams	Performance (unadjusted)	Time (miliseconds)	Dropped
	Constant Removal, Standardization							
94	Mean Imputation, Mode Imputation, Constant Removal, Standardization	Test-Budgeted Statistically Equivalent Signature (SES)	maxK = 2, alpha = 0.05, budget = 3 * nvars	Support Vector Machines (SVM) of type C-SVC	kernel = 'Polynomial Kernel', cost = 1.0, gamma = 0.1, degree = 2	0.6330795553973693	00:00:18.18096	false
95	Mean Imputation, Mode Imputation, Constant Removal, Standardization	Test-Budgeted Statistically Equivalent Signature (SES)	maxK = 3, alpha = 0.05, budget = 3 * nvars	Support Vector Machines (SVM) of type C-SVC	kernel = 'Linear Kernel', cost = 0.01	0.5498299187317406	00:00:38.38726	false
96	Mean Imputation, Mode Imputation, Constant Removal, Standardization	Test-Budgeted Statistically Equivalent Signature (SES)	maxK = 2, alpha = 0.01, budget = 3 * nvars	Support Vector Machines (SVM) of type C-SVC	kernel = 'Gaussian Kernel', cost = 0.1, gamma = 10.0	0.5524809929162156	00:00:00.797	false
97	Mean Imputation, Mode Imputation, Constant Removal, Standardization	LASSO	penalty = 1.0	Support Vector Machines (SVM) of type C-SVC	kernel = 'Gaussian Kernel', cost = 0.1, gamma = 0.1	0.6134989223551977	00:00:04.4612	false
98	Mean Imputation, Mode Imputation, Constant Removal, Standardization	Test-Budgeted Statistically Equivalent Signature (SES)	maxK = 3, alpha = 0.05, budget = 3 * nvars	Support Vector Machines (SVM) of type C-SVC	kernel = 'Linear Kernel', cost = 1.0	0.5498299187317406	00:00:38.38733	false
99	Mean Imputation, Mode Imputation, Constant Removal, Standardization	Test-Budgeted Statistically Equivalent Signature (SES)	maxK = 2, alpha = 0.01, budget = 3 * nvars	Support Vector Machines (SVM) of type C-SVC	kernel = 'Polynomial Kernel', cost = 0.1, gamma = 1.0, degree = 3	0.5793551927155166	00:00:00.784	false
100	Mean Imputation, Mode Imputation, Constant Removal, Standardization	Test-Budgeted Statistically Equivalent Signature (SES)	maxK = 3, alpha = 0.01, budget = 3 * nvars	Support Vector Machines (SVM) of type C-SVC	kernel = 'Linear Kernel', cost = 10.0	0.5468154174884945	00:00:03.3036	false
101	Mean Imputation, Mode Imputation, Constant Removal, Standardization	LASSO	penalty = 1.0	Support Vector Machines (SVM) of type C-SVC	kernel = 'Polynomial Kernel', cost = 0.01, gamma = 0.1, degree = 3	0.5821152363156412	00:00:04.4607	false
102	Mean Imputation, Mode Imputation, Constant	Test-Budgeted Statistically Equivalent Signature (SES)	maxK = 2, alpha = 0.01, budget = 3 * nvars	Support Vector Machines (SVM) of type C-SVC	kernel = 'Polynomial Kernel', cost = 0.01, gamma = 1.0, degree = 2	0.646839423509464	00:00:00.788	false

Configuration	Preprocessing	Name	Hyperparams	Name	Hyperparams	Performance (unadjusted)	Time (miliseconds)	Dropped
	Removal, Standardization							
103	Mean Imputation, Mode Imputation, Constant Removal, Standardization	Test-Budgeted Statistically Equivalent Signature (SES)	maxK = 3, alpha = 0.05, budget = 3 * nvars	Support Vector Machines (SVM) of type C-SVC	kernel = 'Polynomial Kernel', cost = 0.1, gamma = 1.0, degree = 3	0.5	00:00:38.38725	false
104	Mean Imputation, Mode Imputation, Constant Removal, Standardization	LASSO	penalty = 1.0	Support Vector Machines (SVM) of type C-SVC	kernel = 'Gaussian Kernel', cost = 0.01, gamma = 10.0	0.5540435778796102	00:00:04.4619	false
105	Mean Imputation, Mode Imputation, Constant Removal, Standardization	Test-Budgeted Statistically Equivalent Signature (SES)	maxK = 3, alpha = 0.01, budget = 3 * nvars	Ridge Logistic Regression	lambda = 0.1	0.5521842389504332	00:00:03.3026	false
106	Mean Imputation, Mode Imputation, Constant Removal, Standardization	LASSO	penalty = 1.5	Classification Random Forest with Deviance splitting criterion	ntrees = 500, minimum leaf size = 3	0.5	00:00:00.000	false
107	Mean Imputation, Mode Imputation, Constant Removal, Standardization	Test-Budgeted Statistically Equivalent Signature (SES)	maxK = 2, alpha = 0.05, budget = 3 * nvars	Classification Decision Tree with Deviance splitting criterion	minimum leaf size = 3, alpha = 0.01	0.6738025413632701	00:00:18.18094	false
108	Mean Imputation, Mode Imputation, Constant Removal, Standardization	LASSO	penalty = 0.5	Support Vector Machines (SVM) of type C-SVC	kernel = 'Polynomial Kernel', cost = 10.0, gamma = 0.1, degree = 2	0.6634368836291914	00:00:00.006	false
109	Mean Imputation, Mode Imputation, Constant Removal, Standardization	Test-Budgeted Statistically Equivalent Signature (SES)	maxK = 2, alpha = 0.05, budget = 3 * nvars	Support Vector Machines (SVM) of type C-SVC	kernel = 'Polynomial Kernel', cost = 0.1, gamma = 1.0, degree = 2	0.6069873277869229	00:00:18.18098	false
110	Mean Imputation, Mode Imputation, Constant Removal, Standardization	Test-Budgeted Statistically Equivalent Signature (SES)	maxK = 3, alpha = 0.05, budget = 3 * nvars	Support Vector Machines (SVM) of type C-SVC	kernel = 'Gaussian Kernel', cost = 0.01, gamma = 0.01	0.5376321103547824	00:00:38.38731	false
111	Mean Imputation, Mode Imputation, Constant Removal, Standardization	LASSO	penalty = 0.5	Support Vector Machines (SVM) of type C-SVC	kernel = 'Polynomial Kernel', cost = 0.1, gamma = 0.1, degree = 3	0.6735033466308771	00:00:00.006	false

Configuration	Preprocessing	Name	Hyperparams	Name	Hyperparams	Performance (unadjusted)	Time (miliseconds)	Dropped
112	Mean Imputation, Mode Imputation, Constant Removal, Standardization	LASSO	penalty = 0.5	Classification Decision Tree with Deviance splitting criterion	minimum leaf size = 2, alpha = 0.01	0.6783210182754716	00:00:00.018	false
113	Mean Imputation, Mode Imputation, Constant Removal, Standardization	Test-Budgeted Statistically Equivalent Signature (SES)	maxK = 3, alpha = 0.01, budget = 3 * nvars	Support Vector Machines (SVM) of type C-SVC	kernel = 'Gaussian Kernel', cost = 0.01, gamma = 0.1	0.5430545359948193	00:00:03.3048	false
114	Mean Imputation, Mode Imputation, Constant Removal, Standardization	LASSO	penalty = 1.0	Classification Random Forest with Deviance splitting criterion	ntrees = 100, minimum leaf size = 2	0.6330447049576604	00:00:04.4646	false
115	Mean Imputation, Mode Imputation, Constant Removal, Standardization	Test-Budgeted Statistically Equivalent Signature (SES)	maxK = 3, alpha = 0.01, budget = 3 * nvars	Support Vector Machines (SVM) of type C-SVC	kernel = 'Gaussian Kernel', cost = 1.0, gamma = 1.0	0.5185871792400133	00:00:03.3040	false
116	Mean Imputation, Mode Imputation, Constant Removal, Standardization	LASSO	penalty = 0.5	Support Vector Machines (SVM) of type C-SVC	kernel = 'Polynomial Kernel', cost = 0.1, gamma = 0.1, degree = 2	0.6771741669261911	00:00:00.010	false
117	Mean Imputation, Mode Imputation, Constant Removal, Standardization	Test-Budgeted Statistically Equivalent Signature (SES)	maxK = 2, alpha = 0.05, budget = 3 * nvars	Support Vector Machines (SVM) of type C-SVC	kernel = 'Linear Kernel', cost = 1.0	0.6975208917263572	00:00:18.18344	false
118	Mean Imputation, Mode Imputation, Constant Removal, Standardization	Test-Budgeted Statistically Equivalent Signature (SES)	maxK = 2, alpha = 0.01, budget = 3 * nvars	Support Vector Machines (SVM) of type C-SVC	kernel = 'Polynomial Kernel', cost = 1.0, gamma = 0.1, degree = 2	0.6223885959968954	00:00:00.780	false
119	Mean Imputation, Mode Imputation, Constant Removal, Standardization	LASSO	penalty = 0.5	Classification Random Forest with Deviance splitting criterion	ntrees = 100, minimum leaf size = 3	0.7476340629680711	00:00:00.041	false
120	Mean Imputation, Mode Imputation, Constant Removal, Standardization	Test-Budgeted Statistically Equivalent Signature (SES)	maxK = 2, alpha = 0.05, budget = 3 * nvars	Support Vector Machines (SVM) of type C-SVC	kernel = 'Polynomial Kernel', cost = 10.0, gamma = 10.0, degree = 3	0.6138419581992456	00:00:18.18102	false
121	Mean Imputation,	Test-Budgeted	maxK = 2, alpha = 0.01,	Support Vector	kernel = 'Polynomial	0.6088740094812969	00:00:00.780	false

Configuration	Preprocessing	Name	Hyperparams	Name	Hyperparams	Performance (unadjusted)	Time (miliseconds)	Dropped
	Mode Imputation, Constant Removal, Standardization	Statistically Equivalent Signature (SES)	budget = 3 * nvars	Machines (SVM) of type C-SVC	Kernel', cost = 0.1, gamma = 1.0, degree = 2			
122	Mean Imputation, Mode Imputation, Constant Removal, Standardization	LASSO	penalty = 0.5	Classification Random Forest with Deviance splitting criterion	ntrees = 500, minimum leaf size = 2	0.7422279194040328	00:00:00.287	false
123	Mean Imputation, Mode Imputation, Constant Removal, Standardization	LASSO	penalty = 0.5	Classification Random Forest with Deviance splitting criterion	ntrees = 500, minimum leaf size = 4	0.7460788929918486	00:00:00.282	false
124	Mean Imputation, Mode Imputation, Constant Removal, Standardization	LASSO	penalty = 1.5	Classification Random Forest with Deviance splitting criterion	ntrees = 100, minimum leaf size = 4	0.5	00:00:00.000	false
125	Mean Imputation, Mode Imputation, Constant Removal, Standardization	Test-Budgeted Statistically Equivalent Signature (SES)	maxK = 2, alpha = 0.01, budget = 3 * nvars	Classification Random Forest with Deviance splitting criterion	ntrees = 100, minimum leaf size = 4	0.7731908049215741	00:00:00.827	false
126	Mean Imputation, Mode Imputation, Constant Removal, Standardization	Test-Budgeted Statistically Equivalent Signature (SES)	maxK = 3, alpha = 0.01, budget = 3 * nvars	Support Vector Machines (SVM) of type C-SVC	kernel = 'Polynomial Kernel', cost = 0.1, gamma = 10.0, degree = 2	0.5	00:00:03.3035	false
127	Mean Imputation, Mode Imputation, Constant Removal, Standardization	Test-Budgeted Statistically Equivalent Signature (SES)	maxK = 3, alpha = 0.01, budget = 3 * nvars	Support Vector Machines (SVM) of type C-SVC	kernel = 'Gaussian Kernel', cost = 0.01, gamma = 0.01	0.5347029432555749	00:00:03.3048	false
128	Mean Imputation, Mode Imputation, Constant Removal, Standardization	LASSO	penalty = 1.5	Support Vector Machines (SVM) of type C-SVC	kernel = 'Gaussian Kernel', cost = 1.0, gamma = 0.01	0.5	00:00:00.000	false
129	Mean Imputation, Mode Imputation, Constant Removal, Standardization	LASSO	penalty = 0.5	Support Vector Machines (SVM) of type C-SVC	kernel = 'Gaussian Kernel', cost = 0.1, gamma = 0.01	0.6945716732660053	00:00:00.012	false
130	Mean Imputation, Mode Imputation,	Test-Budgeted Statistically Equivalent	maxK = 2, alpha = 0.01, budget = 3 * nvars	Classification Decision Tree with Deviance	minimum leaf size = 3, alpha = 0.01	0.6843190631905207	00:00:00.790	false

Configuration	Preprocessing	Name	Hyperparams	Name	Hyperparams	Performance (unadjusted)	Time (miliseconds)	Dropped
	Constant Removal, Standardization	Signature (SES)		splitting criterion				
131	Mean Imputation, Mode Imputation, Constant Removal, Standardization	LASSO	penalty = 1.5	Classification Random Forest with Deviance splitting criterion	ntrees = 100, minimum leaf size = 3	0.5	00:00:00.000	false
132	Mean Imputation, Mode Imputation, Constant Removal, Standardization	Test-Budgeted Statistically Equivalent Signature (SES)	maxK = 3, alpha = 0.05, budget = 3 * nvars	Support Vector Machines (SVM) of type C-SVC	kernel = 'Polynomial Kernel', cost = 10.0, gamma = 1.0, degree = 3	0.5	00:00:38.38726	false
133	Mean Imputation, Mode Imputation, Constant Removal, Standardization	LASSO	penalty = 0.5	Classification Random Forest with Deviance splitting criterion	ntrees = 100, minimum leaf size = 3	0.740938823884168	00:00:00.057	false
134	Mean Imputation, Mode Imputation, Constant Removal, Standardization	Test-Budgeted Statistically Equivalent Signature (SES)	maxK = 2, alpha = 0.01, budget = 3 * nvars	Support Vector Machines (SVM) of type C-SVC	kernel = 'Polynomial Kernel', cost = 10.0, gamma = 1.0, degree = 3	0.5773926309857484	00:00:00.780	false
135	Mean Imputation, Mode Imputation, Constant Removal, Standardization	LASSO	penalty = 1.5	Support Vector Machines (SVM) of type C-SVC	kernel = 'Polynomial Kernel', cost = 0.1, gamma = 0.01, degree = 2	0.5	00:00:00.000	false
136	Mean Imputation, Mode Imputation, Constant Removal, Standardization	Test-Budgeted Statistically Equivalent Signature (SES)	maxK = 2, alpha = 0.05, budget = 3 * nvars	Classification Random Forest with Deviance splitting criterion	ntrees = 500, minimum leaf size = 4	0.7744401066769487	00:00:18.18421	false
137	Mean Imputation, Mode Imputation, Constant Removal, Standardization	LASSO	penalty = 0.5	Support Vector Machines (SVM) of type C-SVC	kernel = 'Polynomial Kernel', cost = 10.0, gamma = 0.1, degree = 3	0.6669577358089503	00:00:00.010	false
138	Mean Imputation, Mode Imputation, Constant Removal, Standardization	LASSO	penalty = 0.5	Classification Random Forest with Deviance splitting criterion	ntrees = 100, minimum leaf size = 2	0.7393622740282658	00:00:00.059	false
139	Mean Imputation, Mode Imputation, Constant	LASSO	penalty = 1.0	Support Vector Machines (SVM) of type C-SVC	kernel = 'Gaussian Kernel', cost = 10.0, gamma = 10.0	0.5780305670487856	00:00:04.4621	false

Configuration	Preprocessing	Name	Hyperparams	Name	Hyperparams	Performance (unadjusted)	Time (miliseconds)	Dropped
	Removal, Standardization							
140	Mean Imputation, Mode Imputation, Constant Removal, Standardization	LASSO	penalty = 1.5	Ridge Logistic Regression	lambda = 1.0	0.5	00:00:00.000	false
141	Mean Imputation, Mode Imputation, Constant Removal, Standardization	Test-Budgeted Statistically Equivalent Signature (SES)	maxK = 2, alpha = 0.01, budget = 3 * nvars	Support Vector Machines (SVM) of type C-SVC	kernel = 'Polynomial Kernel', cost = 0.1, gamma = 0.01, degree = 2	0.5412517981343892	00:00:00.784	false
142	Mean Imputation, Mode Imputation, Constant Removal, Standardization	LASSO	penalty = 0.5	Support Vector Machines (SVM) of type C-SVC	kernel = 'Polynomial Kernel', cost = 1.0, gamma = 10.0, degree = 3	0.6669608562827187	00:00:00.007	false
143	Mean Imputation, Mode Imputation, Constant Removal, Standardization	LASSO	penalty = 1.5	Support Vector Machines (SVM) of type C-SVC	kernel = 'Polynomial Kernel', cost = 10.0, gamma = 1.0, degree = 2	0.5	00:00:00.000	false
144	Mean Imputation, Mode Imputation, Constant Removal, Standardization	LASSO	penalty = 0.5	Support Vector Machines (SVM) of type C-SVC	kernel = 'Polynomial Kernel', cost = 0.01, gamma = 10.0, degree = 3	0.6669608562827187	00:00:00.023	false
145	Mean Imputation, Mode Imputation, Constant Removal, Standardization	Test-Budgeted Statistically Equivalent Signature (SES)	maxK = 3, alpha = 0.05, budget = 3 * nvars	Classification Random Forest with Deviance splitting criterion	ntrees = 100, minimum leaf size = 2	0.5386738233651189	00:00:38.38736	false
146	Mean Imputation, Mode Imputation, Constant Removal, Standardization	Test-Budgeted Statistically Equivalent Signature (SES)	maxK = 3, alpha = 0.05, budget = 3 * nvars	Support Vector Machines (SVM) of type C-SVC	kernel = 'Linear Kernel', cost = 10.0	0.5498299187317406	00:00:38.38755	false
147	Mean Imputation, Mode Imputation, Constant Removal, Standardization	Test-Budgeted Statistically Equivalent Signature (SES)	maxK = 3, alpha = 0.05, budget = 3 * nvars	Support Vector Machines (SVM) of type C-SVC	kernel = 'Polynomial Kernel', cost = 1.0, gamma = 10.0, degree = 3	0.5	00:00:38.38726	false
148	Mean Imputation, Mode Imputation, Constant Removal, Standardization	LASSO	penalty = 1.5	Classification Decision Tree with Deviance splitting criterion	minimum leaf size = 3, alpha = 0.05	0.5	00:00:00.000	false

Configuration	Preprocessing	Name	Hyperparams	Name	Hyperparams	Performance (unadjusted)	Time (miliseconds)	Dropped
149	Mean Imputation, Mode Imputation, Constant Removal, Standardization	LASSO	penalty = 1.5	Support Vector Machines (SVM) of type C-SVC	kernel = 'Gaussian Kernel', cost = 10.0, gamma = 0.1	0.5	00:00:00.000	false
150	Mean Imputation, Mode Imputation, Constant Removal, Standardization	Test-Budgeted Statistically Equivalent Signature (SES)	maxK = 3, alpha = 0.01, budget = 3 * nvars	Support Vector Machines (SVM) of type C-SVC	kernel = 'Polynomial Kernel', cost = 0.01, gamma = 0.01, degree = 2	0.5409131494910846	00:00:03.3029	false
151	Mean Imputation, Mode Imputation, Constant Removal, Standardization	Test-Budgeted Statistically Equivalent Signature (SES)	maxK = 2, alpha = 0.05, budget = 3 * nvars	Support Vector Machines (SVM) of type C-SVC	kernel = 'Linear Kernel', cost = 0.01	0.7009463068420558	00:00:18.18112	false
152	Mean Imputation, Mode Imputation, Constant Removal, Standardization	Test-Budgeted Statistically Equivalent Signature (SES)	maxK = 3, alpha = 0.05, budget = 3 * nvars	Support Vector Machines (SVM) of type C-SVC	kernel = 'Polynomial Kernel', cost = 10.0, gamma = 0.01, degree = 2	0.4980550797605453	00:00:38.38726	false
153	Mean Imputation, Mode Imputation, Constant Removal, Standardization	LASSO	penalty = 1.5	Classification Random Forest with Deviance splitting criterion	ntrees = 500, minimum leaf size = 4	0.5	00:00:00.000	false
154	Mean Imputation, Mode Imputation, Constant Removal, Standardization	Test-Budgeted Statistically Equivalent Signature (SES)	maxK = 3, alpha = 0.01, budget = 3 * nvars	Support Vector Machines (SVM) of type C-SVC	kernel = 'Gaussian Kernel', cost = 0.1, gamma = 10.0	0.5236425012234729	00:00:03.3040	false
155	Mean Imputation, Mode Imputation, Constant Removal, Standardization	LASSO	penalty = 1.0	Support Vector Machines (SVM) of type C-SVC	kernel = 'Gaussian Kernel', cost = 10.0, gamma = 0.01	0.6015887227931763	00:00:04.4618	false
156	Mean Imputation, Mode Imputation, Constant Removal, Standardization	LASSO	penalty = 1.0	Classification Random Forest with Deviance splitting criterion	ntrees = 100, minimum leaf size = 4	0.635687653552026	00:00:04.4643	false
157	Mean Imputation, Mode Imputation, Constant Removal, Standardization	Test-Budgeted Statistically Equivalent Signature (SES)	maxK = 2, alpha = 0.01, budget = 3 * nvars	Support Vector Machines (SVM) of type C-SVC	kernel = 'Polynomial Kernel', cost = 1.0, gamma = 0.01, degree = 2	0.512263152951412	00:00:00.784	false
158	Mean Imputation,	LASSO	penalty = 1.5	Support Vector	kernel = 'Gaussian	0.5	00:00:00.000	false

Configuration	Preprocessing	Name	Hyperparams	Name	Hyperparams	Performance (unadjusted)	Time (miliseconds)	Dropped
	Mode Imputation, Constant Removal, Standardization			Machines (SVM) of type C-SVC	Kernel', cost = 0.1, gamma = 1.0			
159	Mean Imputation, Mode Imputation, Constant Removal, Standardization	LASSO	penalty = 0.5	Support Vector Machines (SVM) of type C-SVC	kernel = 'Polynomial Kernel', cost = 0.01, gamma = 0.1, degree = 2	0.6533240151661204	00:00:00.010	false
160	Mean Imputation, Mode Imputation, Constant Removal, Standardization	Test-Budgeted Statistically Equivalent Signature (SES)	maxK = 2, alpha = 0.01, budget = 3 * nvars	Ridge Logistic Regression	lambda = 10.0	0.7285717375292275	00:00:00.780	false
161	Mean Imputation, Mode Imputation, Constant Removal, Standardization	Test-Budgeted Statistically Equivalent Signature (SES)	maxK = 3, alpha = 0.05, budget = 3 * nvars	Support Vector Machines (SVM) of type C-SVC	kernel = 'Polynomial Kernel', cost = 0.01, gamma = 1.0, degree = 3	0.5062905970547671	00:00:38.38729	false
162	Mean Imputation, Mode Imputation, Constant Removal, Standardization	LASSO	penalty = 1.0	Support Vector Machines (SVM) of type C-SVC	kernel = 'Polynomial Kernel', cost = 1.0, gamma = 0.01, degree = 3	0.5	00:00:04.4608	false
163	Mean Imputation, Mode Imputation, Constant Removal, Standardization	Test-Budgeted Statistically Equivalent Signature (SES)	maxK = 2, alpha = 0.05, budget = 3 * nvars	Support Vector Machines (SVM) of type C-SVC	kernel = 'Polynomial Kernel', cost = 0.1, gamma = 0.01, degree = 2	0.6483112675673405	00:00:18.18096	false
164	Mean Imputation, Mode Imputation, Constant Removal, Standardization	Test-Budgeted Statistically Equivalent Signature (SES)	maxK = 3, alpha = 0.05, budget = 3 * nvars	Support Vector Machines (SVM) of type C-SVC	kernel = 'Polynomial Kernel', cost = 0.1, gamma = 1.0, degree = 2	0.4862360227491807	00:00:38.38728	false
165	Mean Imputation, Mode Imputation, Constant Removal, Standardization	LASSO	penalty = 1.5	Classification Random Forest with Deviance splitting criterion	ntrees = 100, minimum leaf size = 3	0.5	00:00:00.000	false
166	Mean Imputation, Mode Imputation, Constant Removal, Standardization	Test-Budgeted Statistically Equivalent Signature (SES)	maxK = 3, alpha = 0.01, budget = 3 * nvars	Classification Random Forest with Deviance splitting criterion	ntrees = 500, minimum leaf size = 4	0.5510756983682086	00:00:03.3068	false
167	Mean Imputation, Mode Imputation,	LASSO	penalty = 0.5	Support Vector Machines	kernel = 'Polynomial Kernel', cost = 1.0, gamma =	0.5530797531303604	00:00:00.009	false

Configuration	Preprocessing	Name	Hyperparams	Name	Hyperparams	Performance (unadjusted)	Time (miliseconds)	Dropped
	Constant Removal, Standardization			(SVM) of type C-SVC	10.0, degree = 2			
168	Mean Imputation, Mode Imputation, Constant Removal, Standardization	LASSO	penalty = 0.5	Support Vector Machines (SVM) of type C-SVC	kernel = 'Polynomial Kernel', cost = 10.0, gamma = 0.01, degree = 3	0.507125122965204	00:00:00.006	false
169	Mean Imputation, Mode Imputation, Constant Removal, Standardization	LASSO	penalty = 1.0	Support Vector Machines (SVM) of type C-SVC	kernel = 'Linear Kernel', cost = 0.1	0.6151610535213774	00:00:04.4612	false
170	Mean Imputation, Mode Imputation, Constant Removal, Standardization	LASSO	penalty = 1.0	Classification Random Forest with Deviance splitting criterion	ntrees = 500, minimum leaf size = 4	0.6376822851013135	00:00:04.4720	false
171	Mean Imputation, Mode Imputation, Constant Removal, Standardization	LASSO	penalty = 1.0	Support Vector Machines (SVM) of type C-SVC	kernel = 'Polynomial Kernel', cost = 0.1, gamma = 1.0, degree = 3	0.5923030270449298	00:00:04.4607	false
172	Mean Imputation, Mode Imputation, Constant Removal, Standardization	LASSO	penalty = 1.5	Support Vector Machines (SVM) of type C-SVC	kernel = 'Polynomial Kernel', cost = 0.01, gamma = 0.01, degree = 2	0.5	00:00:00.000	false
173	Mean Imputation, Mode Imputation, Constant Removal, Standardization	LASSO	penalty = 0.5	Classification Random Forest with Deviance splitting criterion	ntrees = 500, minimum leaf size = 2	0.7442222419955213	00:00:00.363	false
174	Mean Imputation, Mode Imputation, Constant Removal, Standardization	Test-Budgeted Statistically Equivalent Signature (SES)	maxK = 3, alpha = 0.05, budget = 3 * nvars	Support Vector Machines (SVM) of type C-SVC	kernel = 'Polynomial Kernel', cost = 1.0, gamma = 1.0, degree = 3	0.5	00:00:38.38726	false
175	Mean Imputation, Mode Imputation, Constant Removal, Standardization	Test-Budgeted Statistically Equivalent Signature (SES)	maxK = 2, alpha = 0.01, budget = 3 * nvars	Support Vector Machines (SVM) of type C-SVC	kernel = 'Gaussian Kernel', cost = 0.1, gamma = 0.1	0.7178367516424196	00:00:00.796	false
176	Mean Imputation, Mode Imputation, Constant	LASSO	penalty = 1.5	Support Vector Machines (SVM) of type C-SVC	kernel = 'Gaussian Kernel', cost = 0.1, gamma = 10.0	0.5	00:00:00.000	false

Configuration	Preprocessing	Name	Hyperparams	Name	Hyperparams	Performance (unadjusted)	Time (miliseconds)	Dropped
	Removal, Standardization							
177	Mean Imputation, Mode Imputation, Constant Removal, Standardization	Test-Budgeted Statistically Equivalent Signature (SES)	maxK = 3, alpha = 0.01, budget = 3 * nvars	Support Vector Machines (SVM) of type C-SVC	kernel = 'Gaussian Kernel', cost = 0.1, gamma = 1.0	0.5283822228154211	00:00:03.3045	false
178	Mean Imputation, Mode Imputation, Constant Removal, Standardization	LASSO	penalty = 1.5	Classification Decision Tree with Deviance splitting criterion	minimum leaf size = 2, alpha = 0.05	0.5	00:00:00.000	false
179	Mean Imputation, Mode Imputation, Constant Removal, Standardization	Test-Budgeted Statistically Equivalent Signature (SES)	maxK = 2, alpha = 0.05, budget = 3 * nvars	Support Vector Machines (SVM) of type C-SVC	kernel = 'Polynomial Kernel', cost = 1.0, gamma = 0.1, degree = 3	0.6211903649656687	00:00:18.18096	false
180	Mean Imputation, Mode Imputation, Constant Removal, Standardization	Test-Budgeted Statistically Equivalent Signature (SES)	maxK = 3, alpha = 0.05, budget = 3 * nvars	Classification Decision Tree with Deviance splitting criterion	minimum leaf size = 4, alpha = 0.01	0.532028728131967	00:00:38.38734	false
181	Mean Imputation, Mode Imputation, Constant Removal, Standardization	Test-Budgeted Statistically Equivalent Signature (SES)	maxK = 2, alpha = 0.05, budget = 3 * nvars	Support Vector Machines (SVM) of type C-SVC	kernel = 'Polynomial Kernel', cost = 0.01, gamma = 0.01, degree = 2	0.6662650833197393	00:00:18.18100	false
182	Mean Imputation, Mode Imputation, Constant Removal, Standardization	Test-Budgeted Statistically Equivalent Signature (SES)	maxK = 2, alpha = 0.05, budget = 3 * nvars	Classification Decision Tree with Deviance splitting criterion	minimum leaf size = 2, alpha = 0.05	0.6297717296199079	00:00:18.18099	false
183	Mean Imputation, Mode Imputation, Constant Removal, Standardization	LASSO	penalty = 1.0	Classification Random Forest with Deviance splitting criterion	ntrees = 100, minimum leaf size = 2	0.6403015057367285	00:00:04.4629	false
184	Mean Imputation, Mode Imputation, Constant Removal, Standardization	Test-Budgeted Statistically Equivalent Signature (SES)	maxK = 2, alpha = 0.05, budget = 3 * nvars	Support Vector Machines (SVM) of type C-SVC	kernel = 'Polynomial Kernel', cost = 0.1, gamma = 0.1, degree = 2	0.6330891948806929	00:00:18.18103	false
185	Mean Imputation, Mode Imputation, Constant Removal, Standardization	Test-Budgeted Statistically Equivalent Signature (SES)	maxK = 2, alpha = 0.01, budget = 3 * nvars	Support Vector Machines (SVM) of type C-SVC	kernel = 'Polynomial Kernel', cost = 0.1, gamma = 10.0, degree = 2	0.573161639305364	00:00:00.780	false

Configuration	Preprocessing	Name	Hyperparams	Name	Hyperparams	Performance (unadjusted)	Time (miliseconds)	Dropped
186	Mean Imputation, Mode Imputation, Constant Removal, Standardization	LASSO	penalty = 1.5	Support Vector Machines (SVM) of type C-SVC	kernel = 'Polynomial Kernel', cost = 10.0, gamma = 0.01, degree = 2	0.5	00:00:00.000	false
187	Mean Imputation, Mode Imputation, Constant Removal, Standardization	LASSO	penalty = 0.5	Support Vector Machines (SVM) of type C-SVC	kernel = 'Linear Kernel', cost = 1.0	0.6967129979781802	00:00:00.033	false
188	Mean Imputation, Mode Imputation, Constant Removal, Standardization	Test-Budgeted Statistically Equivalent Signature (SES)	maxK = 3, alpha = 0.05, budget = 3 * nvars	Support Vector Machines (SVM) of type C-SVC	kernel = 'Gaussian Kernel', cost = 10.0, gamma = 0.1	0.4948618155348925	00:00:38.38731	false
189	Mean Imputation, Mode Imputation, Constant Removal, Standardization	LASSO	penalty = 1.5	Support Vector Machines (SVM) of type C-SVC	kernel = 'Linear Kernel', cost = 10.0	0.5	00:00:00.000	false
190	Mean Imputation, Mode Imputation, Constant Removal, Standardization	LASSO	penalty = 0.5	Support Vector Machines (SVM) of type C-SVC	kernel = 'Polynomial Kernel', cost = 0.01, gamma = 0.01, degree = 3	0.5800337567291008	00:00:00.006	false
191	Mean Imputation, Mode Imputation, Constant Removal, Standardization	LASSO	penalty = 0.5	Support Vector Machines (SVM) of type C-SVC	kernel = 'Gaussian Kernel', cost = 0.01, gamma = 10.0	0.5	00:00:00.015	false
192	Mean Imputation, Mode Imputation, Constant Removal, Standardization	LASSO	penalty = 0.5	Support Vector Machines (SVM) of type C-SVC	kernel = 'Polynomial Kernel', cost = 10.0, gamma = 10.0, degree = 3	0.6669608562827187	00:00:00.008	false
193	Mean Imputation, Mode Imputation, Constant Removal, Standardization	Test-Budgeted Statistically Equivalent Signature (SES)	maxK = 3, alpha = 0.05, budget = 3 * nvars	Support Vector Machines (SVM) of type C-SVC	kernel = 'Gaussian Kernel', cost = 0.1, gamma = 1.0	0.5591228749882596	00:00:38.38735	false
194	Mean Imputation, Mode Imputation, Constant Removal, Standardization	Test-Budgeted Statistically Equivalent Signature (SES)	maxK = 2, alpha = 0.05, budget = 3 * nvars	Classification Random Forest with Deviance splitting criterion	ntrees = 500, minimum leaf size = 2	0.7773567918810833	00:00:18.18436	false
195	Mean Imputation,	Test-Budgeted	maxK = 2, alpha = 0.01,	Support Vector	kernel = 'Polynomial	0.6468389909685457	00:00:00.786	false

Configuration	Preprocessing	Name	Hyperparams	Name	Hyperparams	Performance (unadjusted)	Time (miliseconds)	Dropped
	Mode Imputation, Constant Removal, Standardization	Statistically Equivalent Signature (SES)	budget = 3 * nvars	Machines (SVM) of type C-SVC	Kernel', cost = 10.0, gamma = 10.0, degree = 3			
196	Mean Imputation, Mode Imputation, Constant Removal, Standardization	Test-Budgeted Statistically Equivalent Signature (SES)	maxK = 3, alpha = 0.01, budget = 3 * nvars	Classification Random Forest with Deviance splitting criterion	ntrees = 500, minimum leaf size = 2	0.5533581550028919	00:00:03.3078	false
197	Mean Imputation, Mode Imputation, Constant Removal, Standardization	LASSO	penalty = 1.5	Classification Decision Tree with Deviance splitting criterion	minimum leaf size = 4, alpha = 0.01	0.5	00:00:00.000	false
198	Mean Imputation, Mode Imputation, Constant Removal, Standardization	LASSO	penalty = 0.5	Ridge Logistic Regression	lambda = 0.1	0.7054090477673474	00:00:00.002	false
199	Mean Imputation, Mode Imputation, Constant Removal, Standardization	Test-Budgeted Statistically Equivalent Signature (SES)	maxK = 3, alpha = 0.01, budget = 3 * nvars	Support Vector Machines (SVM) of type C-SVC	kernel = 'Polynomial Kernel', cost = 0.1, gamma = 0.1, degree = 2	0.5257554018181548	00:00:03.3029	false
200	Mean Imputation, Mode Imputation, Constant Removal, Standardization	Test-Budgeted Statistically Equivalent Signature (SES)	maxK = 3, alpha = 0.05, budget = 3 * nvars	Classification Random Forest with Deviance splitting criterion	ntrees = 500, minimum leaf size = 4	0.5445959573489938	00:00:38.38790	false
201	Mean Imputation, Mode Imputation, Constant Removal, Standardization	Test-Budgeted Statistically Equivalent Signature (SES)	maxK = 2, alpha = 0.01, budget = 3 * nvars	Classification Random Forest with Deviance splitting criterion	ntrees = 500, minimum leaf size = 4	0.7769315423667649	00:00:01.1057	false
202	Mean Imputation, Mode Imputation, Constant Removal, Standardization	LASSO	penalty = 1.0	Classification Random Forest with Deviance splitting criterion	ntrees = 500, minimum leaf size = 3	0.6394450747183542	00:00:04.4738	false
203	Mean Imputation, Mode Imputation, Constant Removal, Standardization	LASSO	penalty = 0.5	Classification Random Forest with Deviance splitting criterion	ntrees = 500, minimum leaf size = 2	0.7422279194040328	00:00:00.298	false
204	Mean Imputation, Mode Imputation,	LASSO	penalty = 1.0	Support Vector Machines	kernel = 'Polynomial Kernel', cost = 0.1, gamma =	0.5426613871958001	00:00:04.4614	false

Configuration	Preprocessing	Name	Hyperparams	Name	Hyperparams	Performance (unadjusted)	Time (miliseconds)	Dropped
	Constant Removal, Standardization			(SVM) of type C-SVC	10.0, degree = 2			
205	Mean Imputation, Mode Imputation, Constant Removal, Standardization	Test-Budgeted Statistically Equivalent Signature (SES)	maxK = 3, alpha = 0.01, budget = 3 * nvars	Support Vector Machines (SVM) of type C-SVC	kernel = 'Gaussian Kernel', cost = 10.0, gamma = 0.1	0.5252462702614524	00:00:03.3038	false
206	Mean Imputation, Mode Imputation, Constant Removal, Standardization	Test-Budgeted Statistically Equivalent Signature (SES)	maxK = 2, alpha = 0.01, budget = 3 * nvars	Support Vector Machines (SVM) of type C-SVC	kernel = 'Gaussian Kernel', cost = 0.01, gamma = 0.1	0.6860801844354476	00:00:00.795	false
207	Mean Imputation, Mode Imputation, Constant Removal, Standardization	Test-Budgeted Statistically Equivalent Signature (SES)	maxK = 2, alpha = 0.05, budget = 3 * nvars	Support Vector Machines (SVM) of type C-SVC	kernel = 'Polynomial Kernel', cost = 1.0, gamma = 10.0, degree = 3	0.6138419581992456	00:00:18.18108	false
208	Mean Imputation, Mode Imputation, Constant Removal, Standardization	Test-Budgeted Statistically Equivalent Signature (SES)	maxK = 3, alpha = 0.01, budget = 3 * nvars	Classification Decision Tree with Deviance splitting criterion	minimum leaf size = 4, alpha = 0.01	0.546452608345321	00:00:03.3037	false
209	Mean Imputation, Mode Imputation, Constant Removal, Standardization	Test-Budgeted Statistically Equivalent Signature (SES)	maxK = 2, alpha = 0.01, budget = 3 * nvars	Support Vector Machines (SVM) of type C-SVC	kernel = 'Polynomial Kernel', cost = 0.01, gamma = 0.01, degree = 2	0.5966983224827355	00:00:00.780	false
210	Mean Imputation, Mode Imputation, Constant Removal, Standardization	Test-Budgeted Statistically Equivalent Signature (SES)	maxK = 2, alpha = 0.01, budget = 3 * nvars	Classification Random Forest with Deviance splitting criterion	ntrees = 500, minimum leaf size = 3	0.7765097531797936	00:00:00.983	false
211	Mean Imputation, Mode Imputation, Constant Removal, Standardization	Test-Budgeted Statistically Equivalent Signature (SES)	maxK = 2, alpha = 0.05, budget = 3 * nvars	Classification Random Forest with Deviance splitting criterion	ntrees = 100, minimum leaf size = 2	0.7807008584083484	00:00:18.18160	false
212	Mean Imputation, Mode Imputation, Constant Removal, Standardization	LASSO	penalty = 0.5	Support Vector Machines (SVM) of type C-SVC	kernel = 'Polynomial Kernel', cost = 0.01, gamma = 1.0, degree = 2	0.5841150274601692	00:00:00.005	false
213	Mean Imputation, Mode Imputation, Constant	Test-Budgeted Statistically Equivalent Signature (SES)	maxK = 2, alpha = 0.05, budget = 3 * nvars	Classification Random Forest with Deviance splitting criterion	ntrees = 100, minimum leaf size = 3	0.7813685471074134	00:00:18.18149	false

Configuration	Preprocessing	Name	Hyperparams	Name	Hyperparams	Performance (unadjusted)	Time (miliseconds)	Dropped
	Removal, Standardization							
214	Mean Imputation, Mode Imputation, Constant Removal, Standardization	Test-Budgeted Statistically Equivalent Signature (SES)	maxK = 3, alpha = 0.01, budget = 3 * nvars	Classification Decision Tree with Deviance splitting criterion	minimum leaf size = 3, alpha = 0.05	0.5387201361391645	00:00:03.3044	false
215	Mean Imputation, Mode Imputation, Constant Removal, Standardization	Test-Budgeted Statistically Equivalent Signature (SES)	maxK = 3, alpha = 0.01, budget = 3 * nvars	Support Vector Machines (SVM) of type C-SVC	kernel = 'Polynomial Kernel', cost = 1.0, gamma = 0.01, degree = 2	0.5370217951189611	00:00:03.3042	false
216	Mean Imputation, Mode Imputation, Constant Removal, Standardization	Test-Budgeted Statistically Equivalent Signature (SES)	maxK = 3, alpha = 0.01, budget = 3 * nvars	Ridge Logistic Regression	lambda = 10.0	0.5458884823004256	00:00:03.3026	false
217	Mean Imputation, Mode Imputation, Constant Removal, Standardization	LASSO	penalty = 0.5	Support Vector Machines (SVM) of type C-SVC	kernel = 'Gaussian Kernel', cost = 1.0, gamma = 0.1	0.652699920412471	00:00:00.013	false
218	Mean Imputation, Mode Imputation, Constant Removal, Standardization	Test-Budgeted Statistically Equivalent Signature (SES)	maxK = 3, alpha = 0.01, budget = 3 * nvars	Classification Decision Tree with Deviance splitting criterion	minimum leaf size = 2, alpha = 0.05	0.5427198111155601	00:00:03.3040	false
219	Mean Imputation, Mode Imputation, Constant Removal, Standardization	Test-Budgeted Statistically Equivalent Signature (SES)	maxK = 3, alpha = 0.01, budget = 3 * nvars	Support Vector Machines (SVM) of type C-SVC	kernel = 'Polynomial Kernel', cost = 0.1, gamma = 1.0, degree = 2	0.5359630894296885	00:00:03.3036	false
220	Mean Imputation, Mode Imputation, Constant Removal, Standardization	LASSO	penalty = 1.5	Support Vector Machines (SVM) of type C-SVC	kernel = 'Polynomial Kernel', cost = 0.01, gamma = 1.0, degree = 3	0.5	00:00:00.000	false
221	Mean Imputation, Mode Imputation, Constant Removal, Standardization	Test-Budgeted Statistically Equivalent Signature (SES)	maxK = 2, alpha = 0.01, budget = 3 * nvars	Support Vector Machines (SVM) of type C-SVC	kernel = 'Gaussian Kernel', cost = 10.0, gamma = 0.01	0.7127269603990252	00:00:00.794	false
222	Mean Imputation, Mode Imputation, Constant Removal, Standardization	LASSO	penalty = 1.5	Support Vector Machines (SVM) of type C-SVC	kernel = 'Polynomial Kernel', cost = 1.0, gamma = 0.01, degree = 2	0.5	00:00:00.000	false

Configuration	Preprocessing	Name	Hyperparams	Name	Hyperparams	Performance (unadjusted)	Time (miliseconds)	Dropped
223	Mean Imputation, Mode Imputation, Constant Removal, Standardization	Test-Budgeted Statistically Equivalent Signature (SES)	maxK = 2, alpha = 0.05, budget = 3 * nvars	Support Vector Machines (SVM) of type C-SVC	kernel = 'Gaussian Kernel', cost = 0.1, gamma = 0.1	0.6408206166303332	00:00:18.18104	false
224	Mean Imputation, Mode Imputation, Constant Removal, Standardization	Test-Budgeted Statistically Equivalent Signature (SES)	maxK = 2, alpha = 0.01, budget = 3 * nvars	Classification Random Forest with Deviance splitting criterion	ntrees = 500, minimum leaf size = 4	0.7731622572209618	00:00:01.1090	false
225	Mean Imputation, Mode Imputation, Constant Removal, Standardization	LASSO	penalty = 0.5	Classification Random Forest with Deviance splitting criterion	ntrees = 500, minimum leaf size = 2	0.7473008828778058	00:00:00.218	false
226	Mean Imputation, Mode Imputation, Constant Removal, Standardization	Test-Budgeted Statistically Equivalent Signature (SES)	maxK = 3, alpha = 0.05, budget = 3 * nvars	Support Vector Machines (SVM) of type C-SVC	kernel = 'Polynomial Kernel', cost = 0.01, gamma = 10.0, degree = 3	0.5	00:00:38.38726	false
227	Mean Imputation, Mode Imputation, Constant Removal, Standardization	Test-Budgeted Statistically Equivalent Signature (SES)	maxK = 2, alpha = 0.05, budget = 3 * nvars	Support Vector Machines (SVM) of type C-SVC	kernel = 'Polynomial Kernel', cost = 1.0, gamma = 1.0, degree = 3	0.588100305003139	00:00:18.18102	false
228	Mean Imputation, Mode Imputation, Constant Removal, Standardization	Test-Budgeted Statistically Equivalent Signature (SES)	maxK = 3, alpha = 0.05, budget = 3 * nvars	Support Vector Machines (SVM) of type C-SVC	kernel = 'Polynomial Kernel', cost = 1.0, gamma = 10.0, degree = 2	0.5	00:00:38.38735	false
229	Mean Imputation, Mode Imputation, Constant Removal, Standardization	Test-Budgeted Statistically Equivalent Signature (SES)	maxK = 2, alpha = 0.01, budget = 3 * nvars	Classification Random Forest with Deviance splitting criterion	ntrees = 500, minimum leaf size = 2	0.7729689732220096	00:00:01.1121	false
230	Mean Imputation, Mode Imputation, Constant Removal, Standardization	Test-Budgeted Statistically Equivalent Signature (SES)	maxK = 3, alpha = 0.01, budget = 3 * nvars	Support Vector Machines (SVM) of type C-SVC	kernel = 'Polynomial Kernel', cost = 1.0, gamma = 10.0, degree = 3	0.5	00:00:03.3033	false
231	Mean Imputation, Mode Imputation, Constant Removal, Standardization	Test-Budgeted Statistically Equivalent Signature (SES)	maxK = 3, alpha = 0.01, budget = 3 * nvars	Classification Random Forest with Deviance splitting criterion	ntrees = 100, minimum leaf size = 2	0.5520526847196888	00:00:03.3032	false
232	Mean Imputation,	LASSO	penalty = 1.5	Support Vector	kernel = 'Polynomial	0.5	00:00:00.000	false

Configuration	Preprocessing	Name	Hyperparams	Name	Hyperparams	Performance (unadjusted)	Time (miliseconds)	Dropped
	Mode Imputation, Constant Removal, Standardization			Machines (SVM) of type C-SVC	Kernel', cost = 1.0, gamma = 1.0, degree = 3			
233	Mean Imputation, Mode Imputation, Constant Removal, Standardization	Test-Budgeted Statistically Equivalent Signature (SES)	maxK = 2, alpha = 0.05, budget = 3 * nvars	Classification Random Forest with Deviance splitting criterion	ntrees = 500, minimum leaf size = 3	0.7771985436965194	00:00:18.18354	false
234	Mean Imputation, Mode Imputation, Constant Removal, Standardization	LASSO	penalty = 1.0	Support Vector Machines (SVM) of type C-SVC	kernel = 'Gaussian Kernel', cost = 0.01, gamma = 0.01	0.6177016752927684	00:00:04.4616	false
235	Mean Imputation, Mode Imputation, Constant Removal, Standardization	Test-Budgeted Statistically Equivalent Signature (SES)	maxK = 3, alpha = 0.01, budget = 3 * nvars	Support Vector Machines (SVM) of type C-SVC	kernel = 'Polynomial Kernel', cost = 0.01, gamma = 1.0, degree = 3	0.5	00:00:03.3028	false
236	Mean Imputation, Mode Imputation, Constant Removal, Standardization	LASSO	penalty = 1.0	Support Vector Machines (SVM) of type C-SVC	kernel = 'Polynomial Kernel', cost = 0.1, gamma = 0.01, degree = 3	0.5207206255283178	00:00:04.4622	false
237	Mean Imputation, Mode Imputation, Constant Removal, Standardization	Test-Budgeted Statistically Equivalent Signature (SES)	maxK = 2, alpha = 0.05, budget = 3 * nvars	Support Vector Machines (SVM) of type C-SVC	kernel = 'Polynomial Kernel', cost = 1.0, gamma = 0.01, degree = 2	0.6382996445749483	00:00:18.18097	false
238	Mean Imputation, Mode Imputation, Constant Removal, Standardization	LASSO	penalty = 1.5	Support Vector Machines (SVM) of type C-SVC	kernel = 'Gaussian Kernel', cost = 0.1, gamma = 0.1	0.5	00:00:00.000	false
239	Mean Imputation, Mode Imputation, Constant Removal, Standardization	LASSO	penalty = 1.0	Support Vector Machines (SVM) of type C-SVC	kernel = 'Gaussian Kernel', cost = 0.01, gamma = 0.1	0.6007136925153119	00:00:04.4613	false
240	Mean Imputation, Mode Imputation, Constant Removal, Standardization	Test-Budgeted Statistically Equivalent Signature (SES)	maxK = 2, alpha = 0.05, budget = 3 * nvars	Ridge Logistic Regression	lambda = 10.0	0.7126541699416193	00:00:18.18099	false
241	Mean Imputation, Mode Imputation,	Test-Budgeted Statistically Equivalent	maxK = 2, alpha = 0.05, budget = 3 * nvars	Classification Decision Tree with Deviance	minimum leaf size = 4, alpha = 0.01	0.6805929085534349	00:00:18.18097	false

Configuration	Preprocessing	Name	Hyperparams	Name	Hyperparams	Performance (unadjusted)	Time (miliseconds)	Dropped
	Constant Removal, Standardization	Signature (SES)		splitting criterion				
242	Mean Imputation, Mode Imputation, Constant Removal, Standardization	Test-Budgeted Statistically Equivalent Signature (SES)	maxK = 3, alpha = 0.01, budget = 3 * nvars	Support Vector Machines (SVM) of type C-SVC	kernel = 'Polynomial Kernel', cost = 10.0, gamma = 10.0, degree = 3	0.5	00:00:03.3032	false
243	Mean Imputation, Mode Imputation, Constant Removal, Standardization	Test-Budgeted Statistically Equivalent Signature (SES)	maxK = 3, alpha = 0.05, budget = 3 * nvars	Support Vector Machines (SVM) of type C-SVC	kernel = 'Gaussian Kernel', cost = 0.1, gamma = 0.01	0.5401501164152988	00:00:38.38732	false
244	Mean Imputation, Mode Imputation, Constant Removal, Standardization	LASSO	penalty = 1.0	Support Vector Machines (SVM) of type C-SVC	kernel = 'Polynomial Kernel', cost = 0.1, gamma = 10.0, degree = 3	0.5656502573000548	00:00:04.4620	false
245	Mean Imputation, Mode Imputation, Constant Removal, Standardization	Test-Budgeted Statistically Equivalent Signature (SES)	maxK = 2, alpha = 0.01, budget = 3 * nvars	Support Vector Machines (SVM) of type C-SVC	kernel = 'Linear Kernel', cost = 0.1	0.716949579323061	00:00:00.802	false
246	Mean Imputation, Mode Imputation, Constant Removal, Standardization	LASSO	penalty = 0.5	Support Vector Machines (SVM) of type C-SVC	kernel = 'Polynomial Kernel', cost = 0.1, gamma = 1.0, degree = 2	0.6382993356171494	00:00:00.010	false
247	Mean Imputation, Mode Imputation, Constant Removal, Standardization	Test-Budgeted Statistically Equivalent Signature (SES)	maxK = 2, alpha = 0.01, budget = 3 * nvars	Support Vector Machines (SVM) of type C-SVC	kernel = 'Polynomial Kernel', cost = 0.01, gamma = 10.0, degree = 3	0.6468389909685457	00:00:00.785	false
248	Mean Imputation, Mode Imputation, Constant Removal, Standardization	Test-Budgeted Statistically Equivalent Signature (SES)	maxK = 2, alpha = 0.01, budget = 3 * nvars	Classification Random Forest with Deviance splitting criterion	ntrees = 100, minimum leaf size = 3	0.7779802069275753	00:00:00.830	false
249	Mean Imputation, Mode Imputation, Constant Removal, Standardization	LASSO	penalty = 0.5	Support Vector Machines (SVM) of type C-SVC	kernel = 'Polynomial Kernel', cost = 1.0, gamma = 0.1, degree = 2	0.6634368836291914	00:00:00.006	false
250	Mean Imputation, Mode Imputation, Constant	Test-Budgeted Statistically Equivalent Signature (SES)	maxK = 3, alpha = 0.05, budget = 3 * nvars	Support Vector Machines (SVM) of type C-SVC	kernel = 'Polynomial Kernel', cost = 1.0, gamma = 0.01, degree = 3	0.5	00:00:38.38726	false

Configuration	Preprocessing	Name	Hyperparams	Name	Hyperparams	Performance (unadjusted)	Time (miliseconds)	Dropped
	Removal, Standardization							
251	Mean Imputation, Mode Imputation, Constant Removal, Standardization	LASSO	penalty = 0.5	Support Vector Machines (SVM) of type C-SVC	kernel = 'Polynomial Kernel', cost = 10.0, gamma = 1.0, degree = 3	0.6477983667254922	00:00:00.005	false
252	Mean Imputation, Mode Imputation, Constant Removal, Standardization	LASSO	penalty = 1.5	Support Vector Machines (SVM) of type C-SVC	kernel = 'Gaussian Kernel', cost = 0.1, gamma = 0.01	0.5	00:00:00.000	false
253	Mean Imputation, Mode Imputation, Constant Removal, Standardization	Test-Budgeted Statistically Equivalent Signature (SES)	maxK = 2, alpha = 0.05, budget = 3 * nvars	Support Vector Machines (SVM) of type C-SVC	kernel = 'Gaussian Kernel', cost = 0.01, gamma = 0.1	0.6455737778865309	00:00:18.18105	false
254	Mean Imputation, Mode Imputation, Constant Removal, Standardization	Test-Budgeted Statistically Equivalent Signature (SES)	maxK = 2, alpha = 0.01, budget = 3 * nvars	Support Vector Machines (SVM) of type C-SVC	kernel = 'Gaussian Kernel', cost = 10.0, gamma = 0.1	0.7069937541091387	00:00:00.788	false
255	Mean Imputation, Mode Imputation, Constant Removal, Standardization	LASSO	penalty = 1.0	Classification Decision Tree with Deviance splitting criterion	minimum leaf size = 3, alpha = 0.01	0.6050531283830879	00:00:04.4615	false
256	Mean Imputation, Mode Imputation, Constant Removal, Standardization	Test-Budgeted Statistically Equivalent Signature (SES)	maxK = 2, alpha = 0.01, budget = 3 * nvars	Support Vector Machines (SVM) of type C-SVC	kernel = 'Polynomial Kernel', cost = 1.0, gamma = 0.01, degree = 3	0.5490278951817413	00:00:00.780	false
257	Mean Imputation, Mode Imputation, Constant Removal, Standardization	LASSO	penalty = 1.5	Ridge Logistic Regression	lambda = 10.0	0.5	00:00:00.000	false
258	Mean Imputation, Mode Imputation, Constant Removal, Standardization	LASSO	penalty = 0.5	Support Vector Machines (SVM) of type C-SVC	kernel = 'Polynomial Kernel', cost = 10.0, gamma = 1.0, degree = 2	0.6356511965317633	00:00:00.005	false
259	Mean Imputation, Mode Imputation, Constant Removal, Standardization	Test-Budgeted Statistically Equivalent Signature (SES)	maxK = 3, alpha = 0.05, budget = 3 * nvars	Support Vector Machines (SVM) of type C-SVC	kernel = 'Polynomial Kernel', cost = 1.0, gamma = 0.01, degree = 2	0.4980550797605453	00:00:38.38728	false

Configuration	Preprocessing	Name	Hyperparams	Name	Hyperparams	Performance (unadjusted)	Time (miliseconds)	Dropped
260	Mean Imputation, Mode Imputation, Constant Removal, Standardization	Test-Budgeted Statistically Equivalent Signature (SES)	maxK = 2, alpha = 0.01, budget = 3 * nvars	Classification Decision Tree with Deviance splitting criterion	minimum leaf size = 4, alpha = 0.01	0.6904223700276333	00:00:00.792	false
261	Mean Imputation, Mode Imputation, Constant Removal, Standardization	LASSO	penalty = 1.5	Support Vector Machines (SVM) of type C-SVC	kernel = 'Polynomial Kernel', cost = 0.01, gamma = 10.0, degree = 3	0.5	00:00:00.000	false
262	Mean Imputation, Mode Imputation, Constant Removal, Standardization	Test-Budgeted Statistically Equivalent Signature (SES)	maxK = 3, alpha = 0.05, budget = 3 * nvars	Ridge Logistic Regression	lambda = 10.0	0.5530726162052074	00:00:38.38723	false
263	Mean Imputation, Mode Imputation, Constant Removal, Standardization	LASSO	penalty = 0.5	Support Vector Machines (SVM) of type C-SVC	kernel = 'Gaussian Kernel', cost = 1.0, gamma = 0.01	0.700064016055919	00:00:00.012	false
264	Mean Imputation, Mode Imputation, Constant Removal, Standardization	LASSO	penalty = 0.5	Classification Random Forest with Deviance splitting criterion	ntrees = 100, minimum leaf size = 4	0.7460087595715126	00:00:00.055	false
265	Mean Imputation, Mode Imputation, Constant Removal, Standardization	Test-Budgeted Statistically Equivalent Signature (SES)	maxK = 3, alpha = 0.05, budget = 3 * nvars	Classification Random Forest with Deviance splitting criterion	ntrees = 100, minimum leaf size = 3	0.5419039153603931	00:00:38.38734	false
266	Mean Imputation, Mode Imputation, Constant Removal, Standardization	Test-Budgeted Statistically Equivalent Signature (SES)	maxK = 2, alpha = 0.05, budget = 3 * nvars	Classification Random Forest with Deviance splitting criterion	ntrees = 500, minimum leaf size = 4	0.7702520601306027	00:00:18.18341	false
267	Mean Imputation, Mode Imputation, Constant Removal, Standardization	Test-Budgeted Statistically Equivalent Signature (SES)	maxK = 3, alpha = 0.01, budget = 3 * nvars	Support Vector Machines (SVM) of type C-SVC	kernel = 'Gaussian Kernel', cost = 0.1, gamma = 0.01	0.5456536125817502	00:00:03.3048	false
268	Mean Imputation, Mode Imputation, Constant Removal, Standardization	LASSO	penalty = 0.5	Classification Random Forest with Deviance splitting criterion	ntrees = 500, minimum leaf size = 3	0.7479011260893852	00:00:00.289	false
269	Mean Imputation,	LASSO	penalty = 1.0	Classification Random	ntrees = 100, minimum leaf	0.6382696138769014	00:00:04.4627	false

Configuration	Preprocessing	Name	Hyperparams	Name	Hyperparams	Performance (unadjusted)	Time (miliseconds)	Dropped
	Mode Imputation, Constant Removal, Standardization			Forest with Deviance splitting criterion	size = 4			
270	Mean Imputation, Mode Imputation, Constant Removal, Standardization	Test-Budgeted Statistically Equivalent Signature (SES)	maxK = 2, alpha = 0.05, budget = 3 * nvars	Support Vector Machines (SVM) of type C-SVC	kernel = 'Linear Kernel', cost = 0.1	0.6974084001918011	00:00:18.18113	false
271	Mean Imputation, Mode Imputation, Constant Removal, Standardization	Test-Budgeted Statistically Equivalent Signature (SES)	maxK = 2, alpha = 0.01, budget = 3 * nvars	Classification Random Forest with Deviance splitting criterion	ntrees = 500, minimum leaf size = 4	0.7807594059112278	00:00:00.971	false
272	Mean Imputation, Mode Imputation, Constant Removal, Standardization	Test-Budgeted Statistically Equivalent Signature (SES)	maxK = 3, alpha = 0.01, budget = 3 * nvars	Classification Random Forest with Deviance splitting criterion	ntrees = 100, minimum leaf size = 3	0.5488198738957848	00:00:03.3030	false
273	Mean Imputation, Mode Imputation, Constant Removal, Standardization	LASSO	penalty = 0.5	Classification Random Forest with Deviance splitting criterion	ntrees = 100, minimum leaf size = 3	0.7395426435912267	00:00:00.072	false
274	Mean Imputation, Mode Imputation, Constant Removal, Standardization	Test-Budgeted Statistically Equivalent Signature (SES)	maxK = 2, alpha = 0.05, budget = 3 * nvars	Support Vector Machines (SVM) of type C-SVC	kernel = 'Polynomial Kernel', cost = 0.1, gamma = 10.0, degree = 2	0.5743604573564088	00:00:18.18095	false
275	Mean Imputation, Mode Imputation, Constant Removal, Standardization	LASSO	penalty = 1.5	Support Vector Machines (SVM) of type C-SVC	kernel = 'Polynomial Kernel', cost = 1.0, gamma = 10.0, degree = 3	0.5	00:00:00.000	false
276	Mean Imputation, Mode Imputation, Constant Removal, Standardization	Test-Budgeted Statistically Equivalent Signature (SES)	maxK = 3, alpha = 0.05, budget = 3 * nvars	Classification Decision Tree with Deviance splitting criterion	minimum leaf size = 2, alpha = 0.05	0.5117297991032809	00:00:38.38741	false
277	Mean Imputation, Mode Imputation, Constant Removal, Standardization	LASSO	penalty = 1.5	Classification Random Forest with Deviance splitting criterion	ntrees = 100, minimum leaf size = 2	0.5	00:00:00.000	false
278	Mean Imputation, Mode Imputation,	Test-Budgeted Statistically Equivalent	maxK = 3, alpha = 0.05, budget = 3 * nvars	Support Vector Machines	kernel = 'Polynomial Kernel', cost =	0.4738975458864123	00:00:38.38726	false

Configuration	Preprocessing	Name	Hyperparams	Name	Hyperparams	Performance (unadjusted)	Time (miliseconds)	Dropped
	Constant Removal, Standardization	Signature (SES)		(SVM) of type C-SVC	0.1, gamma = 0.1, degree = 2			
279	Mean Imputation, Mode Imputation, Constant Removal, Standardization	Test-Budgeted Statistically Equivalent Signature (SES)	maxK = 2, alpha = 0.05, budget = 3 * nvars	Support Vector Machines (SVM) of type C-SVC	kernel = 'Gaussian Kernel', cost = 10.0, gamma = 0.1	0.6539085633215187	00:00:18.18104	false
280	Mean Imputation, Mode Imputation, Constant Removal, Standardization	Test-Budgeted Statistically Equivalent Signature (SES)	maxK = 2, alpha = 0.05, budget = 3 * nvars	Support Vector Machines (SVM) of type C-SVC	kernel = 'Linear Kernel', cost = 10.0	0.6987630874523588	00:00:25.25344	false
281	Mean Imputation, Mode Imputation, Constant Removal, Standardization	LASSO	penalty = 1.5	Support Vector Machines (SVM) of type C-SVC	kernel = 'Polynomial Kernel', cost = 0.1, gamma = 0.1, degree = 2	0.5	00:00:00.000	false
282	Mean Imputation, Mode Imputation, Constant Removal, Standardization	Test-Budgeted Statistically Equivalent Signature (SES)	maxK = 2, alpha = 0.01, budget = 3 * nvars	Classification Decision Tree with Deviance splitting criterion	minimum leaf size = 2, alpha = 0.05	0.6399348346210695	00:00:00.790	false
283	Mean Imputation, Mode Imputation, Constant Removal, Standardization	Test-Budgeted Statistically Equivalent Signature (SES)	maxK = 3, alpha = 0.05, budget = 3 * nvars	Classification Random Forest with Deviance splitting criterion	ntrees = 500, minimum leaf size = 3	0.5402149357614946	00:00:38.38797	false
284	Mean Imputation, Mode Imputation, Constant Removal, Standardization	Test-Budgeted Statistically Equivalent Signature (SES)	maxK = 2, alpha = 0.05, budget = 3 * nvars	Support Vector Machines (SVM) of type C-SVC	kernel = 'Polynomial Kernel', cost = 10.0, gamma = 0.01, degree = 2	0.6382996445749483	00:00:18.18097	false
285	Mean Imputation, Mode Imputation, Constant Removal, Standardization	Test-Budgeted Statistically Equivalent Signature (SES)	maxK = 3, alpha = 0.05, budget = 3 * nvars	Classification Random Forest with Deviance splitting criterion	ntrees = 500, minimum leaf size = 2	0.5375148299743442	00:00:38.38807	false
286	Mean Imputation, Mode Imputation, Constant Removal, Standardization	LASSO	penalty = 1.0	Support Vector Machines (SVM) of type C-SVC	kernel = 'Linear Kernel', cost = 0.01	0.6149725892640872	00:00:04.4613	false
287	Mean Imputation, Mode Imputation, Constant	Test-Budgeted Statistically Equivalent Signature (SES)	maxK = 2, alpha = 0.01, budget = 3 * nvars	Classification Random Forest with Deviance splitting criterion	ntrees = 500, minimum leaf size = 2	0.7779667981591059	00:00:01.1050	false

Configuration	Preprocessing	Name	Hyperparams	Name	Hyperparams	Performance (unadjusted)	Time (miliseconds)	Dropped
	Removal, Standardization							
288	Mean Imputation, Mode Imputation, Constant Removal, Standardization	Test-Budgeted Statistically Equivalent Signature (SES)	maxK = 2, alpha = 0.01, budget = 3 * nvars	Classification Random Forest with Deviance splitting criterion	ntrees = 500, minimum leaf size = 3	0.7711981507021993	00:00:01.1111	false
289	Mean Imputation, Mode Imputation, Constant Removal, Standardization	LASSO	penalty = 1.5	Classification Random Forest with Deviance splitting criterion	ntrees = 500, minimum leaf size = 3	0.5	00:00:00.000	false
290	Mean Imputation, Mode Imputation, Constant Removal, Standardization	LASSO	penalty = 1.0	Ridge Logistic Regression	lambda = 1.0	0.6192087096439323	00:00:04.4605	false
291	Mean Imputation, Mode Imputation, Constant Removal, Standardization	LASSO	penalty = 1.5	Classification Random Forest with Deviance splitting criterion	ntrees = 500, minimum leaf size = 2	0.5	00:00:00.000	false
292	Mean Imputation, Mode Imputation, Constant Removal, Standardization	Test-Budgeted Statistically Equivalent Signature (SES)	maxK = 2, alpha = 0.05, budget = 3 * nvars	Support Vector Machines (SVM) of type C-SVC	kernel = 'Gaussian Kernel', cost = 10.0, gamma = 0.01	0.6704051796157058	00:00:18.18108	false
293	Mean Imputation, Mode Imputation, Constant Removal, Standardization	LASSO	penalty = 0.5	Classification Random Forest with Deviance splitting criterion	ntrees = 500, minimum leaf size = 3	0.7468543770669277	00:00:00.279	false
294	Mean Imputation, Mode Imputation, Constant Removal, Standardization	Test-Budgeted Statistically Equivalent Signature (SES)	maxK = 3, alpha = 0.01, budget = 3 * nvars	Classification Random Forest with Deviance splitting criterion	ntrees = 500, minimum leaf size = 2	0.5507265760555233	00:00:03.3090	false
295	Mean Imputation, Mode Imputation, Constant Removal, Standardization	Test-Budgeted Statistically Equivalent Signature (SES)	maxK = 2, alpha = 0.01, budget = 3 * nvars	Support Vector Machines (SVM) of type C-SVC	kernel = 'Polynomial Kernel', cost = 0.1, gamma = 0.1, degree = 2	0.6289063079295872	00:00:00.780	false
296	Mean Imputation, Mode Imputation, Constant Removal, Standardization	Test-Budgeted Statistically Equivalent Signature (SES)	maxK = 3, alpha = 0.01, budget = 3 * nvars	Support Vector Machines (SVM) of type C-SVC	kernel = 'Polynomial Kernel', cost = 1.0, gamma = 10.0, degree = 2	0.5	00:00:03.3035	false

Configuration	Preprocessing	Name	Hyperparams	Name	Hyperparams	Performance (unadjusted)	Time (miliseconds)	Dropped
297	Mean Imputation, Mode Imputation, Constant Removal, Standardization	Test-Budgeted Statistically Equivalent Signature (SES)	maxK = 3, alpha = 0.01, budget = 3 * nvars	Classification Random Forest with Deviance splitting criterion	ntrees = 500, minimum leaf size = 4	0.5497599088945243	00:00:03.3063	false
298	Mean Imputation, Mode Imputation, Constant Removal, Standardization	Test-Budgeted Statistically Equivalent Signature (SES)	maxK = 2, alpha = 0.05, budget = 3 * nvars	Classification Random Forest with Deviance splitting criterion	ntrees = 500, minimum leaf size = 4	0.7717471687107315	00:00:18.18490	false
299	Mean Imputation, Mode Imputation, Constant Removal, Standardization	LASSO	penalty = 1.0	Classification Random Forest with Deviance splitting criterion	ntrees = 500, minimum leaf size = 4	0.6280951392287424	00:00:04.4804	false
300	Mean Imputation, Mode Imputation, Constant Removal, Standardization	LASSO	penalty = 1.5	Classification Random Forest with Deviance splitting criterion	ntrees = 500, minimum leaf size = 2	0.5	00:00:00.000	false
301	Mean Imputation, Mode Imputation, Constant Removal, Standardization	LASSO	penalty = 0.5	Support Vector Machines (SVM) of type C-SVC	kernel = 'Polynomial Kernel', cost = 0.1, gamma = 0.01, degree = 2	0.5245785815623871	00:00:00.005	false
302	Mean Imputation, Mode Imputation, Constant Removal, Standardization	LASSO	penalty = 1.0	Support Vector Machines (SVM) of type C-SVC	kernel = 'Polynomial Kernel', cost = 0.01, gamma = 10.0, degree = 2	0.5319596760639271	00:00:04.4607	false
303	Mean Imputation, Mode Imputation, Constant Removal, Standardization	Test-Budgeted Statistically Equivalent Signature (SES)	maxK = 2, alpha = 0.01, budget = 3 * nvars	Classification Random Forest with Deviance splitting criterion	ntrees = 500, minimum leaf size = 3	0.7751905033787625	00:00:01.1038	false
304	Mean Imputation, Mode Imputation, Constant Removal, Standardization	LASSO	penalty = 1.0	Classification Random Forest with Deviance splitting criterion	ntrees = 100, minimum leaf size = 3	0.6338918672420697	00:00:04.4639	false
305	Mean Imputation, Mode Imputation, Constant Removal, Standardization	LASSO	penalty = 1.0	Classification Decision Tree with Deviance splitting criterion	minimum leaf size = 4, alpha = 0.05	0.5930365237551473	00:00:04.4613	false
306	Mean Imputation,	Test-Budgeted	maxK = 2, alpha = 0.05,	Classification Random	ntrees = 500, minimum leaf	0.7733229770679163	00:00:18.18512	false

Configuration	Preprocessing	Name	Hyperparams	Name	Hyperparams	Performance (unadjusted)	Time (miliseconds)	Dropped
	Mode Imputation, Constant Removal, Standardization	Statistically Equivalent Signature (SES)	budget = 3 * nvars	Forest with Deviance splitting criterion	size = 3			
307	Mean Imputation, Mode Imputation, Constant Removal, Standardization	Test-Budgeted Statistically Equivalent Signature (SES)	maxK = 3, alpha = 0.01, budget = 3 * nvars	Classification Random Forest with Deviance splitting criterion	ntrees = 500, minimum leaf size = 3	0.5517382274720332	00:00:03.3080	false
308	Mean Imputation, Mode Imputation, Constant Removal, Standardization	LASSO	penalty = 1.5	Support Vector Machines (SVM) of type C-SVC	kernel = 'Polynomial Kernel', cost = 10.0, gamma = 0.01, degree = 3	0.5	00:00:00.000	false
309	Mean Imputation, Mode Imputation, Constant Removal, Standardization	Test-Budgeted Statistically Equivalent Signature (SES)	maxK = 3, alpha = 0.01, budget = 3 * nvars	Support Vector Machines (SVM) of type C-SVC	kernel = 'Polynomial Kernel', cost = 0.01, gamma = 10.0, degree = 3	0.5	00:00:03.3032	false
310	Mean Imputation, Mode Imputation, Constant Removal, Standardization	Test-Budgeted Statistically Equivalent Signature (SES)	maxK = 3, alpha = 0.05, budget = 3 * nvars	Classification Random Forest with Deviance splitting criterion	ntrees = 500, minimum leaf size = 2	0.5375148299743442	00:00:38.38803	false
311	Mean Imputation, Mode Imputation, Constant Removal, Standardization	LASSO	penalty = 1.5	Support Vector Machines (SVM) of type C-SVC	kernel = 'Polynomial Kernel', cost = 1.0, gamma = 0.01, degree = 3	0.5	00:00:00.000	false
312	Mean Imputation, Mode Imputation, Constant Removal, Standardization	Test-Budgeted Statistically Equivalent Signature (SES)	maxK = 3, alpha = 0.05, budget = 3 * nvars	Support Vector Machines (SVM) of type C-SVC	kernel = 'Polynomial Kernel', cost = 10.0, gamma = 1.0, degree = 2	0.5	00:00:38.38725	false
313	Mean Imputation, Mode Imputation, Constant Removal, Standardization	LASSO	penalty = 1.0	Classification Random Forest with Deviance splitting criterion	ntrees = 100, minimum leaf size = 3	0.6266103498390948	00:00:04.4641	false
314	Mean Imputation, Mode Imputation, Constant Removal, Standardization	Test-Budgeted Statistically Equivalent Signature (SES)	maxK = 3, alpha = 0.01, budget = 3 * nvars	Support Vector Machines (SVM) of type C-SVC	kernel = 'Polynomial Kernel', cost = 10.0, gamma = 0.1, degree = 3	0.5357264895473398	00:00:03.3028	false
315	Mean Imputation, Mode Imputation,	LASSO	penalty = 0.5	Support Vector Machines	kernel = 'Polynomial Kernel', cost =	0.6356511965317633	00:00:00.009	false

Configuration	Preprocessing	Name	Hyperparams	Name	Hyperparams	Performance (unadjusted)	Time (miliseconds)	Dropped
	Constant Removal, Standardization			(SVM) of type C-SVC	1.0, gamma = 1.0, degree = 2			
316	Mean Imputation, Mode Imputation, Constant Removal, Standardization	LASSO	penalty = 0.5	Support Vector Machines (SVM) of type C-SVC	kernel = 'Gaussian Kernel', cost = 0.1, gamma = 10.0	0.5633229399929803	00:00:00.014	false
317	Mean Imputation, Mode Imputation, Constant Removal, Standardization	Test-Budgeted Statistically Equivalent Signature (SES)	maxK = 2, alpha = 0.05, budget = 3 * nvars	Support Vector Machines (SVM) of type C-SVC	kernel = 'Polynomial Kernel', cost = 10.0, gamma = 0.1, degree = 3	0.6211903649656687	00:00:18.18101	false
318	Mean Imputation, Mode Imputation, Constant Removal, Standardization	LASSO	penalty = 0.5	Classification Decision Tree with Deviance splitting criterion	minimum leaf size = 3, alpha = 0.05	0.6386310327099801	00:00:00.017	false
319	Mean Imputation, Mode Imputation, Constant Removal, Standardization	Test-Budgeted Statistically Equivalent Signature (SES)	maxK = 2, alpha = 0.01, budget = 3 * nvars	Classification Random Forest with Deviance splitting criterion	ntrees = 100, minimum leaf size = 2	0.7792769028092915	00:00:00.832	false
320	Mean Imputation, Mode Imputation, Constant Removal, Standardization	LASSO	penalty = 1.5	Classification Random Forest with Deviance splitting criterion	ntrees = 500, minimum leaf size = 4	0.5	00:00:00.000	false
321	Mean Imputation, Mode Imputation, Constant Removal, Standardization	LASSO	penalty = 1.0	Support Vector Machines (SVM) of type C-SVC	kernel = 'Polynomial Kernel', cost = 0.01, gamma = 0.01, degree = 2	0.5048747979415996	00:00:04.4608	false
322	Mean Imputation, Mode Imputation, Constant Removal, Standardization	Test-Budgeted Statistically Equivalent Signature (SES)	maxK = 3, alpha = 0.05, budget = 3 * nvars	Support Vector Machines (SVM) of type C-SVC	kernel = 'Gaussian Kernel', cost = 0.1, gamma = 0.1	0.5477565956310896	00:00:38.38731	false
323	Mean Imputation, Mode Imputation, Constant Removal, Standardization	Test-Budgeted Statistically Equivalent Signature (SES)	maxK = 2, alpha = 0.01, budget = 3 * nvars	Support Vector Machines (SVM) of type C-SVC	kernel = 'Gaussian Kernel', cost = 0.1, gamma = 0.01	0.7171239242089446	00:00:00.788	false
324	Mean Imputation, Mode Imputation, Constant	Test-Budgeted Statistically Equivalent Signature (SES)	maxK = 3, alpha = 0.01, budget = 3 * nvars	Support Vector Machines (SVM) of type C-SVC	kernel = 'Gaussian Kernel', cost = 10.0, gamma = 0.01	0.53564412139817	00:00:03.3039	false

Configuration	Preprocessing	Name	Hyperparams	Name	Hyperparams	Performance (unadjusted)	Time (miliseconds)	Dropped
	Removal, Standardization							
325	Mean Imputation, Mode Imputation, Constant Removal, Standardization	LASSO	penalty = 1.0	Support Vector Machines (SVM) of type C-SVC	kernel = 'Gaussian Kernel', cost = 10.0, gamma = 1.0	0.605516904934921	00:00:04.4617	false
326	Mean Imputation, Mode Imputation, Constant Removal, Standardization	Test-Budgeted Statistically Equivalent Signature (SES)	maxK = 3, alpha = 0.01, budget = 3 * nvars	Support Vector Machines (SVM) of type C-SVC	kernel = 'Gaussian Kernel', cost = 1.0, gamma = 0.1	0.525341429263494	00:00:03.3038	false
327	Mean Imputation, Mode Imputation, Constant Removal, Standardization	Test-Budgeted Statistically Equivalent Signature (SES)	maxK = 3, alpha = 0.05, budget = 3 * nvars	Support Vector Machines (SVM) of type C-SVC	kernel = 'Gaussian Kernel', cost = 10.0, gamma = 10.0	0.4883740725086879	00:00:38.38736	false
328	Mean Imputation, Mode Imputation, Constant Removal, Standardization	LASSO	penalty = 1.5	Support Vector Machines (SVM) of type C-SVC	kernel = 'Linear Kernel', cost = 0.1	0.5	00:00:00.000	false
329	Mean Imputation, Mode Imputation, Constant Removal, Standardization	LASSO	penalty = 1.5	Support Vector Machines (SVM) of type C-SVC	kernel = 'Polynomial Kernel', cost = 0.1, gamma = 1.0, degree = 3	0.5	00:00:00.000	false
330	Mean Imputation, Mode Imputation, Constant Removal, Standardization	LASSO	penalty = 1.0	Support Vector Machines (SVM) of type C-SVC	kernel = 'Polynomial Kernel', cost = 1.0, gamma = 0.1, degree = 3	0.5601878834166284	00:00:04.4607	false
331	Mean Imputation, Mode Imputation, Constant Removal, Standardization	Test-Budgeted Statistically Equivalent Signature (SES)	maxK = 3, alpha = 0.01, budget = 3 * nvars	Support Vector Machines (SVM) of type C-SVC	kernel = 'Polynomial Kernel', cost = 0.01, gamma = 0.01, degree = 3	0.5400431861211215	00:00:03.3028	false
332	Mean Imputation, Mode Imputation, Constant Removal, Standardization	LASSO	penalty = 1.0	Classification Decision Tree with Deviance splitting criterion	minimum leaf size = 2, alpha = 0.05	0.5546217306085728	00:00:04.4617	false
333	Mean Imputation, Mode Imputation, Constant Removal, Standardization	Test-Budgeted Statistically Equivalent Signature (SES)	maxK = 2, alpha = 0.01, budget = 3 * nvars	Support Vector Machines (SVM) of type C-SVC	kernel = 'Polynomial Kernel', cost = 1.0, gamma = 10.0, degree = 3	0.6468389909685457	00:00:00.789	false

Configuration	Preprocessing	Name	Hyperparams	Name	Hyperparams	Performance (unadjusted)	Time (miliseconds)	Dropped
334	Mean Imputation, Mode Imputation, Constant Removal, Standardization	Test-Budgeted Statistically Equivalent Signature (SES)	maxK = 3, alpha = 0.01, budget = 3 * nvars	Support Vector Machines (SVM) of type C-SVC	kernel = 'Polynomial Kernel', cost = 0.01, gamma = 0.1, degree = 2	0.5446457922419461	00:00:03.3035	false
335	Mean Imputation, Mode Imputation, Constant Removal, Standardization	Test-Budgeted Statistically Equivalent Signature (SES)	maxK = 2, alpha = 0.01, budget = 3 * nvars	Classification Random Forest with Deviance splitting criterion	ntrees = 100, minimum leaf size = 4	0.777771104289323	00:00:00.811	false
336	Mean Imputation, Mode Imputation, Constant Removal, Standardization	Test-Budgeted Statistically Equivalent Signature (SES)	maxK = 2, alpha = 0.05, budget = 3 * nvars	Classification Random Forest with Deviance splitting criterion	ntrees = 100, minimum leaf size = 2	0.7733832856302493	00:00:18.18130	false
337	Mean Imputation, Mode Imputation, Constant Removal, Standardization	LASSO	penalty = 1.5	Classification Random Forest with Deviance splitting criterion	ntrees = 500, minimum leaf size = 4	0.5	00:00:00.000	false
338	Mean Imputation, Mode Imputation, Constant Removal, Standardization	Test-Budgeted Statistically Equivalent Signature (SES)	maxK = 3, alpha = 0.05, budget = 3 * nvars	Classification Random Forest with Deviance splitting criterion	ntrees = 100, minimum leaf size = 4	0.5364381429411793	00:00:38.38733	false
339	Mean Imputation, Mode Imputation, Constant Removal, Standardization	Test-Budgeted Statistically Equivalent Signature (SES)	maxK = 2, alpha = 0.01, budget = 3 * nvars	Support Vector Machines (SVM) of type C-SVC	kernel = 'Polynomial Kernel', cost = 0.01, gamma = 1.0, degree = 3	0.6347428606031845	00:00:00.784	false
340	Mean Imputation, Mode Imputation, Constant Removal, Standardization	Test-Budgeted Statistically Equivalent Signature (SES)	maxK = 2, alpha = 0.05, budget = 3 * nvars	Classification Random Forest with Deviance splitting criterion	ntrees = 100, minimum leaf size = 3	0.7787267725526834	00:00:18.18182	false
341	Mean Imputation, Mode Imputation, Constant Removal, Standardization	Test-Budgeted Statistically Equivalent Signature (SES)	maxK = 2, alpha = 0.01, budget = 3 * nvars	Classification Random Forest with Deviance splitting criterion	ntrees = 100, minimum leaf size = 3	0.7803025191183085	00:00:00.814	false
342	Mean Imputation, Mode Imputation, Constant Removal, Standardization	LASSO	penalty = 1.0	Support Vector Machines (SVM) of type C-SVC	kernel = 'Polynomial Kernel', cost = 0.01, gamma = 1.0, degree = 3	0.5793682307346275	00:00:04.4613	false
343	Mean Imputation,	LASSO	penalty = 1.0	Classification Decision Tree	minimum leaf size = 4, alpha	0.6078797214930818	00:00:04.4616	false

Configuration	Preprocessing	Name	Hyperparams	Name	Hyperparams	Performance (unadjusted)	Time (miliseconds)	Dropped
	Mode Imputation, Constant Removal, Standardization			with Deviance splitting criterion	= 0.01			
344	Mean Imputation, Mode Imputation, Constant Removal, Standardization	Test-Budgeted Statistically Equivalent Signature (SES)	maxK = 2, alpha = 0.05, budget = 3 * nvars	Support Vector Machines (SVM) of type C-SVC	kernel = 'Polynomial Kernel', cost = 0.1, gamma = 10.0, degree = 3	0.6138419581992456	00:00:18.18101	false
345	Mean Imputation, Mode Imputation, Constant Removal, Standardization	Test-Budgeted Statistically Equivalent Signature (SES)	maxK = 3, alpha = 0.05, budget = 3 * nvars	Support Vector Machines (SVM) of type C-SVC	kernel = 'Gaussian Kernel', cost = 1.0, gamma = 10.0	0.4885121766447677	00:00:38.38732	false
346	Mean Imputation, Mode Imputation, Constant Removal, Standardization	Test-Budgeted Statistically Equivalent Signature (SES)	maxK = 3, alpha = 0.01, budget = 3 * nvars	Classification Random Forest with Deviance splitting criterion	ntrees = 100, minimum leaf size = 3	0.550417618256687	00:00:03.3031	false
347	Mean Imputation, Mode Imputation, Constant Removal, Standardization	Test-Budgeted Statistically Equivalent Signature (SES)	maxK = 3, alpha = 0.05, budget = 3 * nvars	Support Vector Machines (SVM) of type C-SVC	kernel = 'Gaussian Kernel', cost = 10.0, gamma = 0.01	0.5195571831452399	00:00:38.38731	false
348	Mean Imputation, Mode Imputation, Constant Removal, Standardization	Test-Budgeted Statistically Equivalent Signature (SES)	maxK = 2, alpha = 0.05, budget = 3 * nvars	Support Vector Machines (SVM) of type C-SVC	kernel = 'Gaussian Kernel', cost = 0.1, gamma = 0.01	0.6604975827141819	00:00:18.18106	false
349	Mean Imputation, Mode Imputation, Constant Removal, Standardization	Test-Budgeted Statistically Equivalent Signature (SES)	maxK = 3, alpha = 0.05, budget = 3 * nvars	Support Vector Machines (SVM) of type C-SVC	kernel = 'Gaussian Kernel', cost = 1.0, gamma = 0.1	0.49495697453693405	00:00:38.38731	false
350	Mean Imputation, Mode Imputation, Constant Removal, Standardization	LASSO	penalty = 1.5	Support Vector Machines (SVM) of type C-SVC	kernel = 'Polynomial Kernel', cost = 1.0, gamma = 10.0, degree = 2	0.5	00:00:00.000	false
351	Mean Imputation, Mode Imputation, Constant Removal, Standardization	Test-Budgeted Statistically Equivalent Signature (SES)	maxK = 2, alpha = 0.05, budget = 3 * nvars	Support Vector Machines (SVM) of type C-SVC	kernel = 'Gaussian Kernel', cost = 10.0, gamma = 10.0	0.5295463140098767	00:00:18.18116	false
352	Mean Imputation, Mode Imputation,	LASSO	penalty = 1.5	Support Vector Machines	kernel = 'Polynomial Kernel', cost = 0.01, gamma	0.5	00:00:00.000	false

Configuration	Preprocessing	Name	Hyperparams	Name	Hyperparams	Performance (unadjusted)	Time (miliseconds)	Dropped
	Constant Removal, Standardization			(SVM) of type C-SVC	= 1.0, degree = 2			
353	Mean Imputation, Mode Imputation, Constant Removal, Standardization	Test-Budgeted Statistically Equivalent Signature (SES)	maxK = 2, alpha = 0.01, budget = 3 * nvars	Support Vector Machines (SVM) of type C-SVC	kernel = 'Polynomial Kernel', cost = 0.1, gamma = 10.0, degree = 3	0.6468389909685457	00:00:00.790	false
354	Mean Imputation, Mode Imputation, Constant Removal, Standardization	LASSO	penalty = 1.5	Support Vector Machines (SVM) of type C-SVC	kernel = 'Polynomial Kernel', cost = 10.0, gamma = 0.1, degree = 2	0.5	00:00:00.000	false
355	Mean Imputation, Mode Imputation, Constant Removal, Standardization	Test-Budgeted Statistically Equivalent Signature (SES)	maxK = 2, alpha = 0.01, budget = 3 * nvars	Support Vector Machines (SVM) of type C-SVC	kernel = 'Polynomial Kernel', cost = 0.01, gamma = 0.01, degree = 3	0.6402588386647091	00:00:00.787	false
356	Mean Imputation, Mode Imputation, Constant Removal, Standardization	Test-Budgeted Statistically Equivalent Signature (SES)	maxK = 3, alpha = 0.01, budget = 3 * nvars	Classification Random Forest with Deviance splitting criterion	ntrees = 100, minimum leaf size = 4	0.553306064718008	00:00:03.3039	false
357	Mean Imputation, Mode Imputation, Constant Removal, Standardization	LASSO	penalty = 1.5	Support Vector Machines (SVM) of type C-SVC	kernel = 'Polynomial Kernel', cost = 0.1, gamma = 10.0, degree = 2	0.5	00:00:00.000	false
358	Mean Imputation, Mode Imputation, Constant Removal, Standardization	Test-Budgeted Statistically Equivalent Signature (SES)	maxK = 3, alpha = 0.05, budget = 3 * nvars	Classification Random Forest with Deviance splitting criterion	ntrees = 100, minimum leaf size = 2	0.5565623872304034	00:00:38.38727	false
359	Mean Imputation, Mode Imputation, Constant Removal, Standardization	Test-Budgeted Statistically Equivalent Signature (SES)	maxK = 2, alpha = 0.05, budget = 3 * nvars	Classification Random Forest with Deviance splitting criterion	ntrees = 100, minimum leaf size = 4	0.7762674684739462	00:00:18.18124	false
360	Mean Imputation, Mode Imputation, Constant Removal, Standardization	LASSO	penalty = 1.5	Support Vector Machines (SVM) of type C-SVC	kernel = 'Linear Kernel', cost = 1.0	0.5	00:00:00.000	false
361	Mean Imputation, Mode Imputation, Constant	LASSO	penalty = 0.5	Support Vector Machines (SVM) of type C-SVC	kernel = 'Linear Kernel', cost = 10.0	0.6967129979781802	00:00:00.147	false

Configuration	Preprocessing	Name	Hyperparams	Name	Hyperparams	Performance (unadjusted)	Time (miliseconds)	Dropped
	Removal, Standardization							
362	Mean Imputation, Mode Imputation, Constant Removal, Standardization	Test-Budgeted Statistically Equivalent Signature (SES)	maxK = 2, alpha = 0.05, budget = 3 * nvars	Classification Random Forest with Deviance splitting criterion	ntrees = 100, minimum leaf size = 3	0.7659326447281913	00:00:18.18127	false
363	Mean Imputation, Mode Imputation, Constant Removal, Standardization	LASSO	penalty = 1.5	Classification Random Forest with Deviance splitting criterion	ntrees = 500, minimum leaf size = 2	0.5	00:00:00.000	false
364	Mean Imputation, Mode Imputation, Constant Removal, Standardization	Test-Budgeted Statistically Equivalent Signature (SES)	maxK = 2, alpha = 0.05, budget = 3 * nvars	Support Vector Machines (SVM) of type C-SVC	kernel = 'Polynomial Kernel', cost = 0.01, gamma = 10.0, degree = 3	0.6138419581992456	00:00:18.18101	false
365	Mean Imputation, Mode Imputation, Constant Removal, Standardization	Test-Budgeted Statistically Equivalent Signature (SES)	maxK = 3, alpha = 0.01, budget = 3 * nvars	Classification Random Forest with Deviance splitting criterion	ntrees = 500, minimum leaf size = 2	0.5533581550028919	00:00:03.3071	false
366	Mean Imputation, Mode Imputation, Constant Removal, Standardization	LASSO	penalty = 1.0	Classification Random Forest with Deviance splitting criterion	ntrees = 100, minimum leaf size = 2	0.6358373127097823	00:00:04.4637	false
367	Mean Imputation, Mode Imputation, Constant Removal, Standardization	LASSO	penalty = 0.5	Support Vector Machines (SVM) of type C-SVC	kernel = 'Polynomial Kernel', cost = 10.0, gamma = 0.01, degree = 2	0.5	00:00:00.005	false
368	Mean Imputation, Mode Imputation, Constant Removal, Standardization	LASSO	penalty = 1.5	Support Vector Machines (SVM) of type C-SVC	kernel = 'Polynomial Kernel', cost = 10.0, gamma = 10.0, degree = 3	0.5	00:00:00.000	false
369	Mean Imputation, Mode Imputation, Constant Removal, Standardization	LASSO	penalty = 0.5	Support Vector Machines (SVM) of type C-SVC	kernel = 'Gaussian Kernel', cost = 10.0, gamma = 0.1	0.6518422535629015	00:00:00.013	false
370	Mean Imputation, Mode Imputation, Constant Removal, Standardization	LASSO	penalty = 1.0	Support Vector Machines (SVM) of type C-SVC	kernel = 'Gaussian Kernel', cost = 1.0, gamma = 1.0	0.6065622636472839	00:00:04.4617	false

Configuration	Preprocessing	Name	Hyperparams	Name	Hyperparams	Performance (unadjusted)	Time (miliseconds)	Dropped
371	Mean Imputation, Mode Imputation, Constant Removal, Standardization	LASSO	penalty = 0.5	Classification Random Forest with Deviance splitting criterion	ntrees = 500, minimum leaf size = 4	0.7472208010163476	00:00:00.273	false
372	Mean Imputation, Mode Imputation, Constant Removal, Standardization	Test-Budgeted Statistically Equivalent Signature (SES)	maxK = 2, alpha = 0.05, budget = 3 * nvars	Support Vector Machines (SVM) of type C-SVC	kernel = 'Polynomial Kernel', cost = 10.0, gamma = 1.0, degree = 2	0.6069873277869229	00:00:18.18098	false
373	Mean Imputation, Mode Imputation, Constant Removal, Standardization	LASSO	penalty = 1.0	Support Vector Machines (SVM) of type C-SVC	kernel = 'Gaussian Kernel', cost = 0.1, gamma = 1.0	0.5945968842223902	00:00:04.4612	false
374	Mean Imputation, Mode Imputation, Constant Removal, Standardization	Test-Budgeted Statistically Equivalent Signature (SES)	maxK = 2, alpha = 0.05, budget = 3 * nvars	Classification Random Forest with Deviance splitting criterion	ntrees = 500, minimum leaf size = 3	0.7774456481440286	00:00:18.18424	false
375	Mean Imputation, Mode Imputation, Constant Removal, Standardization	Test-Budgeted Statistically Equivalent Signature (SES)	maxK = 2, alpha = 0.05, budget = 3 * nvars	Support Vector Machines (SVM) of type C-SVC	kernel = 'Polynomial Kernel', cost = 0.01, gamma = 0.1, degree = 2	0.6709509844631302	00:00:18.18096	false
376	Mean Imputation, Mode Imputation, Constant Removal, Standardization	LASSO	penalty = 1.5	Ridge Logistic Regression	lambda = 0.1	0.5	00:00:00.000	false
377	Mean Imputation, Mode Imputation, Constant Removal, Standardization	Test-Budgeted Statistically Equivalent Signature (SES)	maxK = 3, alpha = 0.05, budget = 3 * nvars	Classification Random Forest with Deviance splitting criterion	ntrees = 500, minimum leaf size = 3	0.5482218242845774	00:00:38.38750	false
378	Mean Imputation, Mode Imputation, Constant Removal, Standardization	Test-Budgeted Statistically Equivalent Signature (SES)	maxK = 2, alpha = 0.05, budget = 3 * nvars	Classification Random Forest with Deviance splitting criterion	ntrees = 100, minimum leaf size = 2	0.7665957299560539	00:00:18.18153	false
379	Mean Imputation, Mode Imputation, Constant Removal, Standardization	LASSO	penalty = 0.5	Support Vector Machines (SVM) of type C-SVC	kernel = 'Gaussian Kernel', cost = 1.0, gamma = 10.0	0.5595771356398886	00:00:00.017	false
380	Mean Imputation,	LASSO	penalty = 0.5	Support Vector	kernel = 'Polynomial	0.6477983667254922	00:00:00.005	false

Configuration	Preprocessing	Name	Hyperparams	Name	Hyperparams	Performance (unadjusted)	Time (miliseconds)	Dropped
	Mode Imputation, Constant Removal, Standardization			Machines (SVM) of type C-SVC	Kernel', cost = 1.0, gamma = 1.0, degree = 3			
381	Mean Imputation, Mode Imputation, Constant Removal, Standardization	Test-Budgeted Statistically Equivalent Signature (SES)	maxK = 3, alpha = 0.01, budget = 3 * nvars	Classification Random Forest with Deviance splitting criterion	ntrees = 500, minimum leaf size = 3	0.5522627142313377	00:00:03.3049	false
382	Mean Imputation, Mode Imputation, Constant Removal, Standardization	Test-Budgeted Statistically Equivalent Signature (SES)	maxK = 2, alpha = 0.01, budget = 3 * nvars	Support Vector Machines (SVM) of type C-SVC	kernel = 'Gaussian Kernel', cost = 1.0, gamma = 0.1	0.7048872489408927	00:00:00.788	false
383	Mean Imputation, Mode Imputation, Constant Removal, Standardization	Test-Budgeted Statistically Equivalent Signature (SES)	maxK = 2, alpha = 0.01, budget = 3 * nvars	Classification Random Forest with Deviance splitting criterion	ntrees = 100, minimum leaf size = 3	0.778637669123499	00:00:00.825	false
384	Mean Imputation, Mode Imputation, Constant Removal, Standardization	Test-Budgeted Statistically Equivalent Signature (SES)	maxK = 3, alpha = 0.01, budget = 3 * nvars	Support Vector Machines (SVM) of type C-SVC	kernel = 'Gaussian Kernel', cost = 0.1, gamma = 0.1	0.5229892408536133	00:00:03.3049	false
385	Mean Imputation, Mode Imputation, Constant Removal, Standardization	Test-Budgeted Statistically Equivalent Signature (SES)	maxK = 3, alpha = 0.01, budget = 3 * nvars	Ridge Logistic Regression	lambda = 1.0	0.5501189796483319	00:00:03.3026	false
386	Mean Imputation, Mode Imputation, Constant Removal, Standardization	Test-Budgeted Statistically Equivalent Signature (SES)	maxK = 2, alpha = 0.05, budget = 3 * nvars	Ridge Logistic Regression	lambda = 1.0	0.7286944555669252	00:00:18.18165	false
387	Mean Imputation, Mode Imputation, Constant Removal, Standardization	Test-Budgeted Statistically Equivalent Signature (SES)	maxK = 2, alpha = 0.01, budget = 3 * nvars	Support Vector Machines (SVM) of type C-SVC	kernel = 'Gaussian Kernel', cost = 0.1, gamma = 1.0	0.6781356127003899	00:00:00.788	false
388	Mean Imputation, Mode Imputation, Constant Removal, Standardization	LASSO	penalty = 1.5	Support Vector Machines (SVM) of type C-SVC	kernel = 'Gaussian Kernel', cost = 0.01, gamma = 0.01	0.5	00:00:00.000	false
389	Mean Imputation, Mode Imputation,	LASSO	penalty = 0.5	Support Vector Machines	kernel = 'Polynomial Kernel', cost = 10.0, gamma	0.5530797531303604	00:00:00.010	false

Configuration	Preprocessing	Name	Hyperparams	Name	Hyperparams	Performance (unadjusted)	Time (miliseconds)	Dropped
	Constant Removal, Standardization			(SVM) of type C-SVC	= 10.0, degree = 2			
390	Mean Imputation, Mode Imputation, Constant Removal, Standardization	Test-Budgeted Statistically Equivalent Signature (SES)	maxK = 3, alpha = 0.05, budget = 3 * nvars	Classification Random Forest with Deviance splitting criterion	ntrees = 500, minimum leaf size = 3	0.5402149357614946	00:00:38.38807	false
391	Mean Imputation, Mode Imputation, Constant Removal, Standardization	LASSO	penalty = 0.5	Support Vector Machines (SVM) of type C-SVC	kernel = 'Gaussian Kernel', cost = 0.1, gamma = 0.1	0.6780754277211767	00:00:00.018	false
392	Mean Imputation, Mode Imputation, Constant Removal, Standardization	LASSO	penalty = 0.5	Support Vector Machines (SVM) of type C-SVC	kernel = 'Gaussian Kernel', cost = 0.01, gamma = 1.0	0.5948703427701403	00:00:00.017	false
393	Mean Imputation, Mode Imputation, Constant Removal, Standardization	Test-Budgeted Statistically Equivalent Signature (SES)	maxK = 3, alpha = 0.05, budget = 3 * nvars	Support Vector Machines (SVM) of type C-SVC	kernel = 'Gaussian Kernel', cost = 0.1, gamma = 10.0	0.5	00:00:38.38731	false
394	Mean Imputation, Mode Imputation, Constant Removal, Standardization	Test-Budgeted Statistically Equivalent Signature (SES)	maxK = 3, alpha = 0.01, budget = 3 * nvars	Support Vector Machines (SVM) of type C-SVC	kernel = 'Gaussian Kernel', cost = 1.0, gamma = 10.0	0.5355795183224333	00:00:03.3039	false
395	Mean Imputation, Mode Imputation, Constant Removal, Standardization	Test-Budgeted Statistically Equivalent Signature (SES)	maxK = 2, alpha = 0.01, budget = 3 * nvars	Support Vector Machines (SVM) of type C-SVC	kernel = 'Linear Kernel', cost = 0.01	0.7179368539692426	00:00:00.781	false
396	Mean Imputation, Mode Imputation, Constant Removal, Standardization	Test-Budgeted Statistically Equivalent Signature (SES)	maxK = 3, alpha = 0.01, budget = 3 * nvars	Classification Random Forest with Deviance splitting criterion	ntrees = 500, minimum leaf size = 3	0.550140483111131	00:00:03.3075	false
397	Mean Imputation, Mode Imputation, Constant Removal, Standardization	Test-Budgeted Statistically Equivalent Signature (SES)	maxK = 3, alpha = 0.05, budget = 3 * nvars	Support Vector Machines (SVM) of type C-SVC	kernel = 'Polynomial Kernel', cost = 0.1, gamma = 10.0, degree = 3	0.5	00:00:38.38726	false
398	Mean Imputation, Mode Imputation, Constant	LASSO	penalty = 1.0	Support Vector Machines (SVM) of type C-SVC	kernel = 'Polynomial Kernel', cost = 10.0, gamma = 0.1, degree = 2	0.5552013972307495	00:00:04.4608	false

Configuration	Preprocessing	Name	Hyperparams	Name	Hyperparams	Performance (unadjusted)	Time (miliseconds)	Dropped
	Removal, Standardization							
399	Mean Imputation, Mode Imputation, Constant Removal, Standardization	LASSO	penalty = 1.5	Support Vector Machines (SVM) of type C-SVC	kernel = 'Polynomial Kernel', cost = 10.0, gamma = 1.0, degree = 3	0.5	00:00:00.000	false
400	Mean Imputation, Mode Imputation, Constant Removal, Standardization	LASSO	penalty = 1.5	Support Vector Machines (SVM) of type C-SVC	kernel = 'Gaussian Kernel', cost = 0.01, gamma = 0.1	0.5	00:00:00.000	false
401	Mean Imputation, Mode Imputation, Constant Removal, Standardization	LASSO	penalty = 1.0	Support Vector Machines (SVM) of type C-SVC	kernel = 'Gaussian Kernel', cost = 0.1, gamma = 0.01	0.6022736822331963	00:00:04.4612	false
402	Mean Imputation, Mode Imputation, Constant Removal, Standardization	LASSO	penalty = 1.0	Classification Decision Tree with Deviance splitting criterion	minimum leaf size = 2, alpha = 0.01	0.5990578949840083	00:00:04.4616	false
403	Mean Imputation, Mode Imputation, Constant Removal, Standardization	Test-Budgeted Statistically Equivalent Signature (SES)	maxK = 2, alpha = 0.05, budget = 3 * nvars	Classification Random Forest with Deviance splitting criterion	ntrees = 500, minimum leaf size = 2	0.7761695906432748	00:00:18.18480	false
404	Mean Imputation, Mode Imputation, Constant Removal, Standardization	LASSO	penalty = 1.0	Classification Random Forest with Deviance splitting criterion	ntrees = 100, minimum leaf size = 4	0.6292008991907777	00:00:04.4635	false
405	Mean Imputation, Mode Imputation, Constant Removal, Standardization	Test-Budgeted Statistically Equivalent Signature (SES)	maxK = 2, alpha = 0.01, budget = 3 * nvars	Classification Random Forest with Deviance splitting criterion	ntrees = 100, minimum leaf size = 4	0.7722271655470037	00:00:00.834	false
406	Mean Imputation, Mode Imputation, Constant Removal, Standardization	LASSO	penalty = 1.0	Ridge Logistic Regression	lambda = 10.0	0.6180837942983691	00:00:04.4608	false
407	Mean Imputation, Mode Imputation, Constant Removal, Standardization	Test-Budgeted Statistically Equivalent Signature (SES)	maxK = 3, alpha = 0.05, budget = 3 * nvars	Classification Random Forest with Deviance splitting criterion	ntrees = 100, minimum leaf size = 4	0.5367354221352197	00:00:38.38726	false

Configuration	Preprocessing	Name	Hyperparams	Name	Hyperparams	Performance (unadjusted)	Time (miliseconds)	Dropped
408	Mean Imputation, Mode Imputation, Constant Removal, Standardization	Test-Budgeted Statistically Equivalent Signature (SES)	maxK = 2, alpha = 0.01, budget = 3 * nvars	Support Vector Machines (SVM) of type C-SVC	kernel = 'Gaussian Kernel', cost = 1.0, gamma = 0.01	0.7129524069048361	00:00:00.790	false
409	Mean Imputation, Mode Imputation, Constant Removal, Standardization	LASSO	penalty = 1.5	Support Vector Machines (SVM) of type C-SVC	kernel = 'Polynomial Kernel', cost = 0.1, gamma = 0.01, degree = 3	0.5	00:00:00.000	false
410	Mean Imputation, Mode Imputation, Constant Removal, Standardization	LASSO	penalty = 0.5	Support Vector Machines (SVM) of type C-SVC	kernel = 'Polynomial Kernel', cost = 1.0, gamma = 0.01, degree = 2	0.5	00:00:00.006	false
411	Mean Imputation, Mode Imputation, Constant Removal, Standardization	Test-Budgeted Statistically Equivalent Signature (SES)	maxK = 3, alpha = 0.01, budget = 3 * nvars	Support Vector Machines (SVM) of type C-SVC	kernel = 'Polynomial Kernel', cost = 10.0, gamma = 1.0, degree = 2	0.5	00:00:03.3036	false
412	Mean Imputation, Mode Imputation, Constant Removal, Standardization	LASSO	penalty = 0.5	Support Vector Machines (SVM) of type C-SVC	kernel = 'Polynomial Kernel', cost = 0.01, gamma = 0.1, degree = 3	0.6524289026313318	00:00:00.006	false
413	Mean Imputation, Mode Imputation, Constant Removal, Standardization	LASSO	penalty = 1.0	Classification Random Forest with Deviance splitting criterion	ntrees = 100, minimum leaf size = 3	0.6413708086785009	00:00:04.4632	false
414	Mean Imputation, Mode Imputation, Constant Removal, Standardization	Test-Budgeted Statistically Equivalent Signature (SES)	maxK = 3, alpha = 0.05, budget = 3 * nvars	Classification Decision Tree with Deviance splitting criterion	minimum leaf size = 3, alpha = 0.05	0.514234025645969	00:00:38.38737	false
415	Mean Imputation, Mode Imputation, Constant Removal, Standardization	LASSO	penalty = 1.5	Classification Decision Tree with Deviance splitting criterion	minimum leaf size = 3, alpha = 0.01	0.5	00:00:00.000	false
416	Mean Imputation, Mode Imputation, Constant Removal, Standardization	LASSO	penalty = 1.0	Support Vector Machines (SVM) of type C-SVC	kernel = 'Polynomial Kernel', cost = 1.0, gamma = 10.0, degree = 3	0.5656502573000548	00:00:04.4609	false
417	Mean Imputation,	Test-Budgeted	maxK = 2, alpha = 0.01,	Classification Random	ntrees = 500, minimum leaf	0.777343877445092	00:00:01.1082	false

Configuration	Preprocessing	Name	Hyperparams	Name	Hyperparams	Performance (unadjusted)	Time (miliseconds)	Dropped
	Mode Imputation, Constant Removal, Standardization	Statistically Equivalent Signature (SES)	budget = 3 * nvars	Forest with Deviance splitting criterion	size = 2			
418	Mean Imputation, Mode Imputation, Constant Removal, Standardization	LASSO	penalty = 1.0	Classification Random Forest with Deviance splitting criterion	ntrees = 500, minimum leaf size = 4	0.6280976108911331	00:00:04.4783	false
419	Mean Imputation, Mode Imputation, Constant Removal, Standardization	LASSO	penalty = 1.5	Support Vector Machines (SVM) of type C-SVC	kernel = 'Polynomial Kernel', cost = 0.1, gamma = 1.0, degree = 2	0.5	00:00:00.000	false
420	Mean Imputation, Mode Imputation, Constant Removal, Standardization	LASSO	penalty = 1.5	Support Vector Machines (SVM) of type C-SVC	kernel = 'Polynomial Kernel', cost = 1.0, gamma = 0.1, degree = 2	0.5	00:00:00.000	false
421	Mean Imputation, Mode Imputation, Constant Removal, Standardization	LASSO	penalty = 1.0	Support Vector Machines (SVM) of type C-SVC	kernel = 'Polynomial Kernel', cost = 0.01, gamma = 10.0, degree = 3	0.5704058593228635	00:00:04.4608	false
422	Mean Imputation, Mode Imputation, Constant Removal, Standardization	LASSO	penalty = 1.0	Support Vector Machines (SVM) of type C-SVC	kernel = 'Polynomial Kernel', cost = 0.1, gamma = 0.1, degree = 3	0.5556581295447691	00:00:04.4607	false
423	Mean Imputation, Mode Imputation, Constant Removal, Standardization	Test-Budgeted Statistically Equivalent Signature (SES)	maxK = 2, alpha = 0.01, budget = 3 * nvars	Support Vector Machines (SVM) of type C-SVC	kernel = 'Polynomial Kernel', cost = 1.0, gamma = 0.1, degree = 3	0.6183106929058346	00:00:00.788	false
424	Mean Imputation, Mode Imputation, Constant Removal, Standardization	Test-Budgeted Statistically Equivalent Signature (SES)	maxK = 3, alpha = 0.01, budget = 3 * nvars	Classification Random Forest with Deviance splitting criterion	ntrees = 100, minimum leaf size = 4	0.5501908741281212	00:00:03.3027	false
425	Mean Imputation, Mode Imputation, Constant Removal, Standardization	Test-Budgeted Statistically Equivalent Signature (SES)	maxK = 3, alpha = 0.01, budget = 3 * nvars	Support Vector Machines (SVM) of type C-SVC	kernel = 'Gaussian Kernel', cost = 1.0, gamma = 0.01	0.5355513722669593	00:00:03.3039	false
426	Mean Imputation, Mode Imputation,	LASSO	penalty = 1.0	Support Vector Machines	kernel = 'Polynomial Kernel', cost = 10.0, gamma	0.5601878834166284	00:00:04.4607	false

Configuration	Preprocessing	Name	Hyperparams	Name	Hyperparams	Performance (unadjusted)	Time (miliseconds)	Dropped
	Constant Removal, Standardization			(SVM) of type C-SVC	= 0.1, degree = 3			
427	Mean Imputation, Mode Imputation, Constant Removal, Standardization	LASSO	penalty = 1.0	Support Vector Machines (SVM) of type C-SVC	kernel = 'Polynomial Kernel', cost = 0.1, gamma = 0.1, degree = 2	0.5586398503655589	00:00:04.4608	false
428	Mean Imputation, Mode Imputation, Constant Removal, Standardization	Test-Budgeted Statistically Equivalent Signature (SES)	maxK = 2, alpha = 0.01, budget = 3 * nvars	Support Vector Machines (SVM) of type C-SVC	kernel = 'Polynomial Kernel', cost = 10.0, gamma = 1.0, degree = 2	0.6038463392208332	00:00:00.780	false
429	Mean Imputation, Mode Imputation, Constant Removal, Standardization	Test-Budgeted Statistically Equivalent Signature (SES)	maxK = 3, alpha = 0.05, budget = 3 * nvars	Classification Random Forest with Deviance splitting criterion	ntrees = 500, minimum leaf size = 3	0.5402149357614946	00:00:38.38799	false
430	Mean Imputation, Mode Imputation, Constant Removal, Standardization	LASSO	penalty = 1.5	Support Vector Machines (SVM) of type C-SVC	kernel = 'Linear Kernel', cost = 0.01	0.5	00:00:00.000	false
431	Mean Imputation, Mode Imputation, Constant Removal, Standardization	Test-Budgeted Statistically Equivalent Signature (SES)	maxK = 3, alpha = 0.05, budget = 3 * nvars	Classification Random Forest with Deviance splitting criterion	ntrees = 100, minimum leaf size = 3	0.5419039153603931	00:00:38.38738	false
432	Mean Imputation, Mode Imputation, Constant Removal, Standardization	LASSO	penalty = 1.0	Classification Random Forest with Deviance splitting criterion	ntrees = 500, minimum leaf size = 3	0.630133272036106	00:00:04.4834	false
433	Mean Imputation, Mode Imputation, Constant Removal, Standardization	Test-Budgeted Statistically Equivalent Signature (SES)	maxK = 2, alpha = 0.05, budget = 3 * nvars	Classification Random Forest with Deviance splitting criterion	ntrees = 100, minimum leaf size = 4	0.7759752561878068	00:00:18.18156	false
434	Mean Imputation, Mode Imputation, Constant Removal, Standardization	LASSO	penalty = 1.0	Classification Random Forest with Deviance splitting criterion	ntrees = 500, minimum leaf size = 2	0.6290369661827151	00:00:04.4772	false
435	Mean Imputation, Mode Imputation, Constant	Test-Budgeted Statistically Equivalent Signature (SES)	maxK = 2, alpha = 0.05, budget = 3 * nvars	Support Vector Machines (SVM) of type C-SVC	kernel = 'Gaussian Kernel', cost = 0.1, gamma = 1.0	0.6059909697814557	00:00:18.18110	false

Configuration	Preprocessing	Name	Hyperparams	Name	Hyperparams	Performance (unadjusted)	Time (miliseconds)	Dropped
	Removal, Standardization							
436	Mean Imputation, Mode Imputation, Constant Removal, Standardization	Test-Budgeted Statistically Equivalent Signature (SES)	maxK = 3, alpha = 0.01, budget = 3 * nvars	Classification Random Forest with Deviance splitting criterion	ntrees = 100, minimum leaf size = 3	0.5488198738957848	00:00:03.3036	false
437	Mean Imputation, Mode Imputation, Constant Removal, Standardization	Test-Budgeted Statistically Equivalent Signature (SES)	maxK = 2, alpha = 0.05, budget = 3 * nvars	Support Vector Machines (SVM) of type C-SVC	kernel = 'Gaussian Kernel', cost = 1.0, gamma = 0.1	0.650965616704483	00:00:18.18108	false
438	Mean Imputation, Mode Imputation, Constant Removal, Standardization	Test-Budgeted Statistically Equivalent Signature (SES)	maxK = 2, alpha = 0.01, budget = 3 * nvars	Ridge Logistic Regression	lambda = 1.0	0.745330041079029	00:00:00.778	false
439	Mean Imputation, Mode Imputation, Constant Removal, Standardization	Test-Budgeted Statistically Equivalent Signature (SES)	maxK = 2, alpha = 0.01, budget = 3 * nvars	Classification Random Forest with Deviance splitting criterion	ntrees = 100, minimum leaf size = 3	0.7687364985441908	00:00:00.843	false
440	Mean Imputation, Mode Imputation, Constant Removal, Standardization	Test-Budgeted Statistically Equivalent Signature (SES)	maxK = 3, alpha = 0.01, budget = 3 * nvars	Support Vector Machines (SVM) of type C-SVC	kernel = 'Polynomial Kernel', cost = 0.1, gamma = 10.0, degree = 3	0.5	00:00:03.3031	false
441	Mean Imputation, Mode Imputation, Constant Removal, Standardization	LASSO	penalty = 1.5	Classification Random Forest with Deviance splitting criterion	ntrees = 100, minimum leaf size = 4	0.5	00:00:00.000	false
442	Mean Imputation, Mode Imputation, Constant Removal, Standardization	Test-Budgeted Statistically Equivalent Signature (SES)	maxK = 3, alpha = 0.05, budget = 3 * nvars	Ridge Logistic Regression	lambda = 1.0	0.5504876898854631	00:00:38.38723	false
443	Mean Imputation, Mode Imputation, Constant Removal, Standardization	Test-Budgeted Statistically Equivalent Signature (SES)	maxK = 2, alpha = 0.05, budget = 3 * nvars	Support Vector Machines (SVM) of type C-SVC	kernel = 'Polynomial Kernel', cost = 0.01, gamma = 1.0, degree = 3	0.6083117062874148	00:00:18.18102	false
444	Mean Imputation, Mode Imputation, Constant Removal, Standardization	LASSO	penalty = 0.5	Classification Random Forest with Deviance splitting criterion	ntrees = 100, minimum leaf size = 2	0.7430298502666924	00:00:00.071	false

Configuration	Preprocessing	Name	Hyperparams	Name	Hyperparams	Performance (unadjusted)	Time (miliseconds)	Dropped
445	Mean Imputation, Mode Imputation, Constant Removal, Standardization	Test-Budgeted Statistically Equivalent Signature (SES)	maxK = 3, alpha = 0.05, budget = 3 * nvars	Support Vector Machines (SVM) of type C-SVC	kernel = 'Polynomial Kernel', cost = 0.1, gamma = 0.01, degree = 2	0.5355125362716456	00:00:38.38726	false
446	Mean Imputation, Mode Imputation, Constant Removal, Standardization	Test-Budgeted Statistically Equivalent Signature (SES)	maxK = 3, alpha = 0.01, budget = 3 * nvars	Classification Random Forest with Deviance splitting criterion	ntrees = 500, minimum leaf size = 3	0.550140483111131	00:00:03.3075	false
447	Mean Imputation, Mode Imputation, Constant Removal, Standardization	LASSO	penalty = 1.0	Classification Random Forest with Deviance splitting criterion	ntrees = 100, minimum leaf size = 2	0.6306196334030342	00:00:04.4642	false
448	Mean Imputation, Mode Imputation, Constant Removal, Standardization	LASSO	penalty = 1.0	Support Vector Machines (SVM) of type C-SVC	kernel = 'Polynomial Kernel', cost = 0.01, gamma = 0.1, degree = 2	0.5799560538426936	00:00:04.4608	false
449	Mean Imputation, Mode Imputation, Constant Removal, Standardization	LASSO	penalty = 0.5	Support Vector Machines (SVM) of type C-SVC	kernel = 'Gaussian Kernel', cost = 10.0, gamma = 10.0	0.5614806246385192	00:00:00.017	false
450	Mean Imputation, Mode Imputation, Constant Removal, Standardization	Test-Budgeted Statistically Equivalent Signature (SES)	maxK = 2, alpha = 0.05, budget = 3 * nvars	Support Vector Machines (SVM) of type C-SVC	kernel = 'Gaussian Kernel', cost = 0.1, gamma = 10.0	0.5	00:00:18.18112	false
451	Mean Imputation, Mode Imputation, Constant Removal, Standardization	Test-Budgeted Statistically Equivalent Signature (SES)	maxK = 3, alpha = 0.01, budget = 3 * nvars	Support Vector Machines (SVM) of type C-SVC	kernel = 'Gaussian Kernel', cost = 10.0, gamma = 1.0	0.5188714822065025	00:00:03.3040	false
452	Mean Imputation, Mode Imputation, Constant Removal, Standardization	Test-Budgeted Statistically Equivalent Signature (SES)	maxK = 2, alpha = 0.01, budget = 3 * nvars	Support Vector Machines (SVM) of type C-SVC	kernel = 'Gaussian Kernel', cost = 1.0, gamma = 10.0	0.579761008784288	00:00:00.801	false
453	Mean Imputation, Mode Imputation, Constant Removal, Standardization	Test-Budgeted Statistically Equivalent Signature (SES)	maxK = 3, alpha = 0.01, budget = 3 * nvars	Support Vector Machines (SVM) of type C-SVC	kernel = 'Linear Kernel', cost = 0.01	0.5442895638998877	00:00:03.3034	false
454	Mean Imputation,	LASSO	penalty = 1.0	Support Vector	kernel = 'Gaussian	0.5816001109776414	00:00:04.4616	false

Configuration	Preprocessing	Name	Hyperparams	Name	Hyperparams	Performance (unadjusted)	Time (miliseconds)	Dropped
	Mode Imputation, Constant Removal, Standardization			Machines (SVM) of type C-SVC	Kernel', cost = 1.0, gamma = 0.1			
455	Mean Imputation, Mode Imputation, Constant Removal, Standardization	Test-Budgeted Statistically Equivalent Signature (SES)	maxK = 2, alpha = 0.05, budget = 3 * nvars	Classification Random Forest with Deviance splitting criterion	ntrees = 100, minimum leaf size = 2	0.7808003119237937	00:00:18.18144	false
456	Mean Imputation, Mode Imputation, Constant Removal, Standardization	Test-Budgeted Statistically Equivalent Signature (SES)	maxK = 2, alpha = 0.05, budget = 3 * nvars	Support Vector Machines (SVM) of type C-SVC	kernel = 'Polynomial Kernel', cost = 1.0, gamma = 1.0, degree = 2	0.6069873277869229	00:00:18.18095	false
457	Mean Imputation, Mode Imputation, Constant Removal, Standardization	Test-Budgeted Statistically Equivalent Signature (SES)	maxK = 3, alpha = 0.01, budget = 3 * nvars	Support Vector Machines (SVM) of type C-SVC	kernel = 'Polynomial Kernel', cost = 1.0, gamma = 1.0, degree = 2	0.5	00:00:03.3036	false
458	Mean Imputation, Mode Imputation, Constant Removal, Standardization	LASSO	penalty = 0.5	Support Vector Machines (SVM) of type C-SVC	kernel = 'Polynomial Kernel', cost = 1.0, gamma = 0.01, degree = 3	0.507125122965204	00:00:00.011	false
459	Mean Imputation, Mode Imputation, Constant Removal, Standardization	LASSO	penalty = 1.5	Classification Random Forest with Deviance splitting criterion	ntrees = 500, minimum leaf size = 4	0.5	00:00:00.000	false
460	Mean Imputation, Mode Imputation, Constant Removal, Standardization	Test-Budgeted Statistically Equivalent Signature (SES)	maxK = 2, alpha = 0.01, budget = 3 * nvars	Support Vector Machines (SVM) of type C-SVC	kernel = 'Gaussian Kernel', cost = 0.01, gamma = 1.0	0.6602310757169055	00:00:00.793	false
461	Mean Imputation, Mode Imputation, Constant Removal, Standardization	LASSO	penalty = 1.5	Support Vector Machines (SVM) of type C-SVC	kernel = 'Polynomial Kernel', cost = 0.01, gamma = 10.0, degree = 2	0.5	00:00:00.000	false
462	Mean Imputation, Mode Imputation, Constant Removal, Standardization	LASSO	penalty = 1.5	Classification Random Forest with Deviance splitting criterion	ntrees = 100, minimum leaf size = 3	0.5	00:00:00.000	false
463	Mean Imputation, Mode Imputation,	LASSO	penalty = 0.5	Classification Random Forest with Deviance	ntrees = 100, minimum leaf size = 2	0.749236936028434	00:00:00.043	false

Configuration	Preprocessing	Name	Hyperparams	Name	Hyperparams	Performance (unadjusted)	Time (miliseconds)	Dropped
	Constant Removal, Standardization			splitting criterion				
464	Mean Imputation, Mode Imputation, Constant Removal, Standardization	LASSO	penalty = 0.5	Classification Random Forest with Deviance splitting criterion	ntrees = 500, minimum leaf size = 3	0.7480654916383662	00:00:00.351	false
465	Mean Imputation, Mode Imputation, Constant Removal, Standardization	Test-Budgeted Statistically Equivalent Signature (SES)	maxK = 3, alpha = 0.01, budget = 3 * nvars	Support Vector Machines (SVM) of type C-SVC	kernel = 'Polynomial Kernel', cost = 0.1, gamma = 0.01, degree = 2	0.5405364681427435	00:00:03.3041	false
466	Mean Imputation, Mode Imputation, Constant Removal, Standardization	Test-Budgeted Statistically Equivalent Signature (SES)	maxK = 2, alpha = 0.05, budget = 3 * nvars	Classification Decision Tree with Deviance splitting criterion	minimum leaf size = 4, alpha = 0.05	0.6659895547547369	00:00:18.18094	false
467	Mean Imputation, Mode Imputation, Constant Removal, Standardization	Test-Budgeted Statistically Equivalent Signature (SES)	maxK = 2, alpha = 0.05, budget = 3 * nvars	Support Vector Machines (SVM) of type C-SVC	kernel = 'Polynomial Kernel', cost = 0.01, gamma = 10.0, degree = 2	0.5743604573564088	00:00:18.18095	false
468	Mean Imputation, Mode Imputation, Constant Removal, Standardization	Test-Budgeted Statistically Equivalent Signature (SES)	maxK = 2, alpha = 0.01, budget = 3 * nvars	Classification Random Forest with Deviance splitting criterion	ntrees = 100, minimum leaf size = 4	0.7753087106325972	00:00:00.821	false
469	Mean Imputation, Mode Imputation, Constant Removal, Standardization	LASSO	penalty = 0.5	Support Vector Machines (SVM) of type C-SVC	kernel = 'Polynomial Kernel', cost = 0.1, gamma = 1.0, degree = 3	0.6463786747440593	00:00:00.005	false
470	Mean Imputation, Mode Imputation, Constant Removal, Standardization	Test-Budgeted Statistically Equivalent Signature (SES)	maxK = 3, alpha = 0.05, budget = 3 * nvars	Classification Decision Tree with Deviance splitting criterion	minimum leaf size = 3, alpha = 0.01	0.532028728131967	00:00:38.38742	false
471	Mean Imputation, Mode Imputation, Constant Removal, Standardization	LASSO	penalty = 0.5	Support Vector Machines (SVM) of type C-SVC	kernel = 'Gaussian Kernel', cost = 10.0, gamma = 0.01	0.6999700928850727	00:00:00.012	false
472	Mean Imputation, Mode Imputation, Constant	LASSO	penalty = 1.5	Classification Decision Tree with Deviance splitting criterion	minimum leaf size = 4, alpha = 0.05	0.5	00:00:00.000	false

Configuration	Preprocessing	Name	Hyperparams	Name	Hyperparams	Performance (unadjusted)	Time (miliseconds)	Dropped
	Removal, Standardization							
473	Mean Imputation, Mode Imputation, Constant Removal, Standardization	Test-Budgeted Statistically Equivalent Signature (SES)	maxK = 3, alpha = 0.01, budget = 3 * nvars	Classification Decision Tree with Deviance splitting criterion	minimum leaf size = 4, alpha = 0.05	0.5448103122698265	00:00:03.3033	false
474	Mean Imputation, Mode Imputation, Constant Removal, Standardization	Test-Budgeted Statistically Equivalent Signature (SES)	maxK = 3, alpha = 0.05, budget = 3 * nvars	Support Vector Machines (SVM) of type C-SVC	kernel = 'Polynomial Kernel', cost = 10.0, gamma = 10.0, degree = 2	0.5	00:00:38.38731	false
475	Mean Imputation, Mode Imputation, Constant Removal, Standardization	Test-Budgeted Statistically Equivalent Signature (SES)	maxK = 3, alpha = 0.01, budget = 3 * nvars	Support Vector Machines (SVM) of type C-SVC	kernel = 'Polynomial Kernel', cost = 0.01, gamma = 10.0, degree = 2	0.5409445396034465	00:00:03.3036	false
476	Mean Imputation, Mode Imputation, Constant Removal, Standardization	Test-Budgeted Statistically Equivalent Signature (SES)	maxK = 2, alpha = 0.01, budget = 3 * nvars	Support Vector Machines (SVM) of type C-SVC	kernel = 'Polynomial Kernel', cost = 0.1, gamma = 0.01, degree = 3	0.5791837520329424	00:00:00.780	false
477	Mean Imputation, Mode Imputation, Constant Removal, Standardization	Test-Budgeted Statistically Equivalent Signature (SES)	maxK = 2, alpha = 0.05, budget = 3 * nvars	Classification Random Forest with Deviance splitting criterion	ntrees = 100, minimum leaf size = 3	0.773764601345573	00:00:18.18143	false
478	Mean Imputation, Mode Imputation, Constant Removal, Standardization	Test-Budgeted Statistically Equivalent Signature (SES)	maxK = 2, alpha = 0.05, budget = 3 * nvars	Classification Decision Tree with Deviance splitting criterion	minimum leaf size = 3, alpha = 0.05	0.6480715163154435	00:00:18.18097	false
479	Mean Imputation, Mode Imputation, Constant Removal, Standardization	LASSO	penalty = 1.5	Support Vector Machines (SVM) of type C-SVC	kernel = 'Gaussian Kernel', cost = 10.0, gamma = 1.0	0.5	00:00:00.000	false
480	Mean Imputation, Mode Imputation, Constant Removal, Standardization	Test-Budgeted Statistically Equivalent Signature (SES)	maxK = 3, alpha = 0.01, budget = 3 * nvars	Classification Random Forest with Deviance splitting criterion	ntrees = 500, minimum leaf size = 4	0.5497599088945243	00:00:03.3076	false
481	Mean Imputation, Mode Imputation, Constant Removal, Standardization	LASSO	penalty = 1.5	Support Vector Machines (SVM) of type C-SVC	kernel = 'Polynomial Kernel', cost = 1.0, gamma = 1.0, degree = 2	0.5	00:00:00.000	false

Configuration	Preprocessing	Name	Hyperparams	Name	Hyperparams	Performance (unadjusted)	Time (miliseconds)	Dropped
482	Mean Imputation, Mode Imputation, Constant Removal, Standardization	Test-Budgeted Statistically Equivalent Signature (SES)	maxK = 3, alpha = 0.05, budget = 3 * nvars	Support Vector Machines (SVM) of type C-SVC	kernel = 'Gaussian Kernel', cost = 1.0, gamma = 0.01	0.5194620241431982	00:00:38.38731	false
483	Mean Imputation, Mode Imputation, Constant Removal, Standardization	LASSO	penalty = 1.0	Support Vector Machines (SVM) of type C-SVC	kernel = 'Polynomial Kernel', cost = 1.0, gamma = 10.0, degree = 2	0.5432774490466799	00:00:04.4608	false
484	Mean Imputation, Mode Imputation, Constant Removal, Standardization	LASSO	penalty = 1.0	Support Vector Machines (SVM) of type C-SVC	kernel = 'Polynomial Kernel', cost = 10.0, gamma = 0.01, degree = 3	0.5	00:00:04.4607	false
485	Mean Imputation, Mode Imputation, Constant Removal, Standardization	Test-Budgeted Statistically Equivalent Signature (SES)	maxK = 3, alpha = 0.01, budget = 3 * nvars	Classification Random Forest with Deviance splitting criterion	ntrees = 100, minimum leaf size = 2	0.553603436599388	00:00:03.3031	false
486	Mean Imputation, Mode Imputation, Constant Removal, Standardization	LASSO	penalty = 1.0	Classification Random Forest with Deviance splitting criterion	ntrees = 500, minimum leaf size = 2	0.6267825629161662	00:00:04.4816	false
487	Mean Imputation, Mode Imputation, Constant Removal, Standardization	Test-Budgeted Statistically Equivalent Signature (SES)	maxK = 2, alpha = 0.05, budget = 3 * nvars	Support Vector Machines (SVM) of type C-SVC	kernel = 'Gaussian Kernel', cost = 10.0, gamma = 1.0	0.605348337559876	00:00:18.18108	false
488	Mean Imputation, Mode Imputation, Constant Removal, Standardization	Test-Budgeted Statistically Equivalent Signature (SES)	maxK = 2, alpha = 0.05, budget = 3 * nvars	Classification Random Forest with Deviance splitting criterion	ntrees = 500, minimum leaf size = 3	0.7751097418101468	00:00:18.18467	false
489	Mean Imputation, Mode Imputation, Constant Removal, Standardization	LASSO	penalty = 1.5	Support Vector Machines (SVM) of type C-SVC	kernel = 'Polynomial Kernel', cost = 1.0, gamma = 0.1, degree = 3	0.5	00:00:00.000	false
490	Mean Imputation, Mode Imputation, Constant Removal, Standardization	LASSO	penalty = 1.0	Ridge Logistic Regression	lambda = 0.1	0.6195785939206992	00:00:04.4608	false
491	Mean Imputation,	Test-Budgeted	maxK = 3, alpha = 0.05,	Classification Random	ntrees = 100, minimum leaf	0.5419039153603931	00:00:38.38743	false

Configuration	Preprocessing	Name	Hyperparams	Name	Hyperparams	Performance (unadjusted)	Time (miliseconds)	Dropped
	Mode Imputation, Constant Removal, Standardization	Statistically Equivalent Signature (SES)	budget = 3 * nvars	Forest with Deviance splitting criterion	size = 3			
492	Mean Imputation, Mode Imputation, Constant Removal, Standardization	Test-Budgeted Statistically Equivalent Signature (SES)	maxK = 3, alpha = 0.05, budget = 3 * nvars	Classification Random Forest with Deviance splitting criterion	ntrees = 500, minimum leaf size = 4	0.5445959573489938	00:00:38.38784	false
493	Mean Imputation, Mode Imputation, Constant Removal, Standardization	LASSO	penalty = 1.0	Classification Random Forest with Deviance splitting criterion	ntrees = 500, minimum leaf size = 2	0.6271585027657902	00:00:04.4793	false
494	Mean Imputation, Mode Imputation, Constant Removal, Standardization	LASSO	penalty = 0.5	Support Vector Machines (SVM) of type C-SVC	kernel = 'Polynomial Kernel', cost = 0.1, gamma = 10.0, degree = 2	0.5530797531303604	00:00:00.005	false
495	Mean Imputation, Mode Imputation, Constant Removal, Standardization	Test-Budgeted Statistically Equivalent Signature (SES)	maxK = 2, alpha = 0.05, budget = 3 * nvars	Support Vector Machines (SVM) of type C-SVC	kernel = 'Polynomial Kernel', cost = 0.1, gamma = 0.1, degree = 3	0.6212855239677103	00:00:18.18099	false
496	Mean Imputation, Mode Imputation, Constant Removal, Standardization	LASSO	penalty = 1.0	Support Vector Machines (SVM) of type C-SVC	kernel = 'Linear Kernel', cost = 1.0	0.6149250097630665	00:00:04.4618	false
497	Mean Imputation, Mode Imputation, Constant Removal, Standardization	LASSO	penalty = 1.0	Support Vector Machines (SVM) of type C-SVC	kernel = 'Polynomial Kernel', cost = 10.0, gamma = 10.0, degree = 3	0.5656502573000548	00:00:04.4616	false
498	Mean Imputation, Mode Imputation, Constant Removal, Standardization	Test-Budgeted Statistically Equivalent Signature (SES)	maxK = 2, alpha = 0.05, budget = 3 * nvars	Support Vector Machines (SVM) of type C-SVC	kernel = 'Gaussian Kernel', cost = 1.0, gamma = 0.01	0.6706441275773258	00:00:18.18103	false
499	Mean Imputation, Mode Imputation, Constant Removal, Standardization	LASSO	penalty = 1.0	Support Vector Machines (SVM) of type C-SVC	kernel = 'Polynomial Kernel', cost = 0.01, gamma = 1.0, degree = 2	0.5737929018799464	00:00:04.4613	false
500	Mean Imputation, Mode Imputation,	LASSO	penalty = 0.5	Support Vector Machines	kernel = 'Linear Kernel', cost = 0.1	0.6967129979781802	00:00:00.012	false

Configuration	Preprocessing	Name	Hyperparams	Name	Hyperparams	Performance (unadjusted)	Time (miliseconds)	Dropped
	Constant Removal, Standardization			(SVM) of type C-SVC				
501	Mean Imputation, Mode Imputation, Constant Removal, Standardization	LASSO	penalty = 0.5	Classification Random Forest with Deviance splitting criterion	ntrees = 500, minimum leaf size = 4	0.7500596288551754	00:00:00.204	false
502	Mean Imputation, Mode Imputation, Constant Removal, Standardization	Test-Budgeted Statistically Equivalent Signature (SES)	maxK = 2, alpha = 0.01, budget = 3 * nvars	Ridge Logistic Regression	lambda = 0.1	0.761523446189438	00:00:00.780	false
503	Mean Imputation, Mode Imputation, Constant Removal, Standardization	Test-Budgeted Statistically Equivalent Signature (SES)	maxK = 2, alpha = 0.01, budget = 3 * nvars	Classification Random Forest with Deviance splitting criterion	ntrees = 500, minimum leaf size = 3	0.7720184336581097	00:00:01.1067	false
504	Mean Imputation, Mode Imputation, Constant Removal, Standardization	LASSO	penalty = 1.5	Classification Random Forest with Deviance splitting criterion	ntrees = 100, minimum leaf size = 2	0.5	00:00:00.000	false
505	Mean Imputation, Mode Imputation, Constant Removal, Standardization	Test-Budgeted Statistically Equivalent Signature (SES)	maxK = 3, alpha = 0.05, budget = 3 * nvars	Classification Random Forest with Deviance splitting criterion	ntrees = 100, minimum leaf size = 2	0.5386738233651189	00:00:38.38736	false
506	Mean Imputation, Mode Imputation, Constant Removal, Standardization	Test-Budgeted Statistically Equivalent Signature (SES)	maxK = 2, alpha = 0.01, budget = 3 * nvars	Support Vector Machines (SVM) of type C-SVC	kernel = 'Polynomial Kernel', cost = 10.0, gamma = 0.1, degree = 2	0.6223885959968954	00:00:00.781	false
507	Mean Imputation, Mode Imputation, Constant Removal, Standardization	Test-Budgeted Statistically Equivalent Signature (SES)	maxK = 2, alpha = 0.01, budget = 3 * nvars	Support Vector Machines (SVM) of type C-SVC	kernel = 'Gaussian Kernel', cost = 10.0, gamma = 1.0	0.7016597521911286	00:00:00.791	false
508	Mean Imputation, Mode Imputation, Constant Removal, Standardization	LASSO	penalty = 1.5	Support Vector Machines (SVM) of type C-SVC	kernel = 'Gaussian Kernel', cost = 1.0, gamma = 1.0	0.5	00:00:00.000	false
509	Mean Imputation, Mode Imputation, Constant	Test-Budgeted Statistically Equivalent Signature (SES)	maxK = 3, alpha = 0.05, budget = 3 * nvars	Support Vector Machines (SVM) of type C-SVC	kernel = 'Polynomial Kernel', cost = 0.01, gamma = 0.01, degree = 3	0.5215928133944329	00:00:38.38726	false

Configuration	Preprocessing	Name	Hyperparams	Name	Hyperparams	Performance (unadjusted)	Time (miliseconds)	Dropped
	Removal, Standardization							
510	Mean Imputation, Mode Imputation, Constant Removal, Standardization	Test-Budgeted Statistically Equivalent Signature (SES)	maxK = 2, alpha = 0.01, budget = 3 * nvars	Support Vector Machines (SVM) of type C-SVC	kernel = 'Gaussian Kernel', cost = 1.0, gamma = 1.0	0.7019535401620423	00:00:00.790	false
511	Mean Imputation, Mode Imputation, Constant Removal, Standardization	Test-Budgeted Statistically Equivalent Signature (SES)	maxK = 3, alpha = 0.01, budget = 3 * nvars	Support Vector Machines (SVM) of type C-SVC	kernel = 'Polynomial Kernel', cost = 0.1, gamma = 0.1, degree = 3	0.548474150118887	00:00:03.3028	false
512	Mean Imputation, Mode Imputation, Constant Removal, Standardization	Test-Budgeted Statistically Equivalent Signature (SES)	maxK = 3, alpha = 0.01, budget = 3 * nvars	Classification Decision Tree with Deviance splitting criterion	minimum leaf size = 3, alpha = 0.01	0.546452608345321	00:00:03.3044	false
513	Mean Imputation, Mode Imputation, Constant Removal, Standardization	LASSO	penalty = 1.0	Support Vector Machines (SVM) of type C-SVC	kernel = 'Polynomial Kernel', cost = 0.01, gamma = 0.01, degree = 3	0.5315154565407603	00:00:04.4613	false
514	Mean Imputation, Mode Imputation, Constant Removal, Standardization	Test-Budgeted Statistically Equivalent Signature (SES)	maxK = 3, alpha = 0.01, budget = 3 * nvars	Support Vector Machines (SVM) of type C-SVC	kernel = 'Polynomial Kernel', cost = 1.0, gamma = 0.1, degree = 3	0.5357264895473398	00:00:03.3028	false
515	Mean Imputation, Mode Imputation, Constant Removal, Standardization	Test-Budgeted Statistically Equivalent Signature (SES)	maxK = 3, alpha = 0.01, budget = 3 * nvars	Classification Random Forest with Deviance splitting criterion	ntrees = 100, minimum leaf size = 4	0.5515673429134967	00:00:03.3030	false
516	Mean Imputation, Mode Imputation, Constant Removal, Standardization	Test-Budgeted Statistically Equivalent Signature (SES)	maxK = 2, alpha = 0.01, budget = 3 * nvars	Classification Random Forest with Deviance splitting criterion	ntrees = 500, minimum leaf size = 2	0.7808480767994939	00:00:00.995	false
517	Mean Imputation, Mode Imputation, Constant Removal, Standardization	LASSO	penalty = 0.5	Classification Random Forest with Deviance splitting criterion	ntrees = 100, minimum leaf size = 4	0.746165030426164	00:00:00.068	false
518	Mean Imputation, Mode Imputation, Constant Removal, Standardization	LASSO	penalty = 1.0	Classification Random Forest with Deviance splitting criterion	ntrees = 500, minimum leaf size = 3	0.6338542361821713	00:00:04.4769	false

Configuration	Preprocessing	Name	Hyperparams	Name	Hyperparams	Performance (unadjusted)	Time (miliseconds)	Dropped
519	Mean Imputation, Mode Imputation, Constant Removal, Standardization	Test-Budgeted Statistically Equivalent Signature (SES)	maxK = 2, alpha = 0.05, budget = 3 * nvars	Classification Random Forest with Deviance splitting criterion	ntrees = 500, minimum leaf size = 2	0.7773178631984301	00:00:18.18367	false
520	Mean Imputation, Mode Imputation, Constant Removal, Standardization	Test-Budgeted Statistically Equivalent Signature (SES)	maxK = 2, alpha = 0.01, budget = 3 * nvars	Classification Decision Tree with Deviance splitting criterion	minimum leaf size = 4, alpha = 0.05	0.6654658712857093	00:00:00.791	false
521	Mean Imputation, Mode Imputation, Constant Removal, Standardization	Test-Budgeted Statistically Equivalent Signature (SES)	maxK = 3, alpha = 0.01, budget = 3 * nvars	Classification Decision Tree with Deviance splitting criterion	minimum leaf size = 2, alpha = 0.01	0.5396963192003679	00:00:03.3049	false
522	Mean Imputation, Mode Imputation, Constant Removal, Standardization	Test-Budgeted Statistically Equivalent Signature (SES)	maxK = 3, alpha = 0.01, budget = 3 * nvars	Support Vector Machines (SVM) of type C-SVC	kernel = 'Polynomial Kernel', cost = 0.1, gamma = 0.01, degree = 3	0.515477117349587	00:00:03.3028	false
523	Mean Imputation, Mode Imputation, Constant Removal, Standardization	Test-Budgeted Statistically Equivalent Signature (SES)	maxK = 3, alpha = 0.01, budget = 3 * nvars	Support Vector Machines (SVM) of type C-SVC	kernel = 'Polynomial Kernel', cost = 10.0, gamma = 0.1, degree = 2	0.527929352473887	00:00:03.3029	false
524	Mean Imputation, Mode Imputation, Constant Removal, Standardization	Test-Budgeted Statistically Equivalent Signature (SES)	maxK = 2, alpha = 0.01, budget = 3 * nvars	Support Vector Machines (SVM) of type C-SVC	kernel = 'Polynomial Kernel', cost = 0.01, gamma = 10.0, degree = 2	0.5753027786428596	00:00:00.781	false
525	Mean Imputation, Mode Imputation, Constant Removal, Standardization	Test-Budgeted Statistically Equivalent Signature (SES)	maxK = 3, alpha = 0.01, budget = 3 * nvars	Support Vector Machines (SVM) of type C-SVC	kernel = 'Polynomial Kernel', cost = 0.01, gamma = 0.1, degree = 3	0.5	00:00:03.3028	false
526	Mean Imputation, Mode Imputation, Constant Removal, Standardization	Test-Budgeted Statistically Equivalent Signature (SES)	maxK = 2, alpha = 0.05, budget = 3 * nvars	Support Vector Machines (SVM) of type C-SVC	kernel = 'Polynomial Kernel', cost = 0.01, gamma = 0.1, degree = 3	0.639992856895691	00:00:18.18096	false
527	Mean Imputation, Mode Imputation, Constant Removal, Standardization	Test-Budgeted Statistically Equivalent Signature (SES)	maxK = 2, alpha = 0.05, budget = 3 * nvars	Support Vector Machines (SVM) of type C-SVC	kernel = 'Gaussian Kernel', cost = 0.01, gamma = 1.0	0.5826709278126283	00:00:18.18105	false
528	Mean Imputation,	Test-Budgeted	maxK = 2, alpha = 0.05,	Classification Decision Tree	minimum leaf size = 2, alpha	0.6581493798599062	00:00:18.18095	false

Configuration	Preprocessing	Name	Hyperparams	Name	Hyperparams	Performance (unadjusted)	Time (miliseconds)	Dropped
	Mode Imputation, Constant Removal, Standardization	Statistically Equivalent Signature (SES)	budget = 3 * nvars	with Deviance splitting criterion	= 0.01			
529	Mean Imputation, Mode Imputation, Constant Removal, Standardization	LASSO	penalty = 0.5	Support Vector Machines (SVM) of type C-SVC	kernel = 'Polynomial Kernel', cost = 0.1, gamma = 0.01, degree = 3	0.5754538899022705	00:00:00.006	false
530	Mean Imputation, Mode Imputation, Constant Removal, Standardization	Test-Budgeted Statistically Equivalent Signature (SES)	maxK = 2, alpha = 0.01, budget = 3 * nvars	Classification Random Forest with Deviance splitting criterion	ntrees = 100, minimum leaf size = 2	0.7762115471123568	00:00:00.815	false
531	Mean Imputation, Mode Imputation, Constant Removal, Standardization	Test-Budgeted Statistically Equivalent Signature (SES)	maxK = 2, alpha = 0.01, budget = 3 * nvars	Classification Random Forest with Deviance splitting criterion	ntrees = 100, minimum leaf size = 2	0.7775420429772657	00:00:00.826	false
532	Mean Imputation, Mode Imputation, Constant Removal, Standardization	LASSO	penalty = 1.5	Support Vector Machines (SVM) of type C-SVC	kernel = 'Gaussian Kernel', cost = 1.0, gamma = 10.0	0.5	00:00:00.000	false
533	IdentityFactory	FullSelector	-	Trivial model	-	0.5	00:00:00.000	false
534	Mean Imputation, Mode Imputation, Constant Removal, Standardization	Test-Budgeted Statistically Equivalent Signature (SES)	maxK = 3, alpha = 0.05, budget = 3 * nvars	Support Vector Machines (SVM) of type C-SVC	kernel = 'Gaussian Kernel', cost = 0.01, gamma = 10.0	0.5291099111190204	00:00:38.38737	false
535	Mean Imputation, Mode Imputation, Constant Removal, Standardization	Test-Budgeted Statistically Equivalent Signature (SES)	maxK = 3, alpha = 0.05, budget = 3 * nvars	Support Vector Machines (SVM) of type C-SVC	kernel = 'Gaussian Kernel', cost = 10.0, gamma = 1.0	0.5208734360556222	00:00:38.38736	false
536	Mean Imputation, Mode Imputation, Constant Removal, Standardization	Test-Budgeted Statistically Equivalent Signature (SES)	maxK = 2, alpha = 0.01, budget = 3 * nvars	Support Vector Machines (SVM) of type C-SVC	kernel = 'Polynomial Kernel', cost = 0.01, gamma = 0.1, degree = 2	0.6911133232489507	00:00:00.781	false
537	Mean Imputation, Mode Imputation, Constant Removal, Standardization	LASSO	penalty = 0.5	Ridge Logistic Regression	lambda = 1.0	0.7041116721784739	00:00:00.005	false
538	Mean Imputation,	LASSO	penalty = 1.0	Classification Random	ntrees = 500, minimum leaf	0.6388845016881455	00:00:04.4730	false

Configuration	Preprocessing	Name	Hyperparams	Name	Hyperparams	Performance (unadjusted)	Time (miliseconds)	Dropped
	Mode Imputation, Constant Removal, Standardization			Forest with Deviance splitting criterion	size = 2			
539	Mean Imputation, Mode Imputation, Constant Removal, Standardization	Test-Budgeted Statistically Equivalent Signature (SES)	maxK = 2, alpha = 0.01, budget = 3 * nvars	Support Vector Machines (SVM) of type C-SVC	kernel = 'Gaussian Kernel', cost = 10.0, gamma = 10.0	0.5706051371031128	00:00:00.792	false
540	Mean Imputation, Mode Imputation, Constant Removal, Standardization	LASSO	penalty = 1.0	Support Vector Machines (SVM) of type C-SVC	kernel = 'Polynomial Kernel', cost = 10.0, gamma = 1.0, degree = 3	0.5751495355746368	00:00:04.4621	false
541	Mean Imputation, Mode Imputation, Constant Removal, Standardization	Test-Budgeted Statistically Equivalent Signature (SES)	maxK = 3, alpha = 0.05, budget = 3 * nvars	Support Vector Machines (SVM) of type C-SVC	kernel = 'Polynomial Kernel', cost = 1.0, gamma = 1.0, degree = 2	0.5	00:00:38.38728	false
542	Mean Imputation, Mode Imputation, Constant Removal, Standardization	LASSO	penalty = 1.5	Support Vector Machines (SVM) of type C-SVC	kernel = 'Polynomial Kernel', cost = 0.01, gamma = 0.1, degree = 3	0.5	00:00:00.000	false
543	Mean Imputation, Mode Imputation, Constant Removal, Standardization	Test-Budgeted Statistically Equivalent Signature (SES)	maxK = 3, alpha = 0.01, budget = 3 * nvars	Support Vector Machines (SVM) of type C-SVC	kernel = 'Gaussian Kernel', cost = 0.01, gamma = 1.0	0.5211174818209231	00:00:03.3045	false
544	Mean Imputation, Mode Imputation, Constant Removal, Standardization	Test-Budgeted Statistically Equivalent Signature (SES)	maxK = 2, alpha = 0.01, budget = 3 * nvars	Support Vector Machines (SVM) of type C-SVC	kernel = 'Polynomial Kernel', cost = 1.0, gamma = 1.0, degree = 2	0.6038463392208332	00:00:00.781	false
545	Mean Imputation, Mode Imputation, Constant Removal, Standardization	LASSO	penalty = 0.5	Classification Random Forest with Deviance splitting criterion	ntrees = 500, minimum leaf size = 3	0.745840871903625	00:00:00.210	false
546	Mean Imputation, Mode Imputation, Constant Removal, Standardization	LASSO	penalty = 0.5	Classification Decision Tree with Deviance splitting criterion	minimum leaf size = 3, alpha = 0.01	0.6782030272920961	00:00:00.017	false
547	Mean Imputation, Mode Imputation,	Test-Budgeted Statistically Equivalent	maxK = 2, alpha = 0.05, budget = 3 * nvars	Classification Random Forest with Deviance	ntrees = 100, minimum leaf size = 4	0.7728044222983494	00:00:18.18141	false

Configuration	Preprocessing	Name	Hyperparams	Name	Hyperparams	Performance (unadjusted)	Time (miliseconds)	Dropped
	Constant Removal, Standardization	Signature (SES)		splitting criterion				
548	Mean Imputation, Mode Imputation, Constant Removal, Standardization	LASSO	penalty = 0.5	Support Vector Machines (SVM) of type C-SVC	kernel = 'Gaussian Kernel', cost = 0.01, gamma = 0.1	0.6527644616966478	00:00:00.017	false
549	Mean Imputation, Mode Imputation, Constant Removal, Standardization	Test-Budgeted Statistically Equivalent Signature (SES)	maxK = 3, alpha = 0.05, budget = 3 * nvars	Classification Decision Tree with Deviance splitting criterion	minimum leaf size = 4, alpha = 0.05	0.5167382521886571	00:00:38.38738	false
550	Mean Imputation, Mode Imputation, Constant Removal, Standardization	Test-Budgeted Statistically Equivalent Signature (SES)	maxK = 2, alpha = 0.01, budget = 3 * nvars	Support Vector Machines (SVM) of type C-SVC	kernel = 'Polynomial Kernel', cost = 10.0, gamma = 0.1, degree = 3	0.6183106929058346	00:00:00.780	false
551	Mean Imputation, Mode Imputation, Constant Removal, Standardization	Test-Budgeted Statistically Equivalent Signature (SES)	maxK = 3, alpha = 0.05, budget = 3 * nvars	Classification Random Forest with Deviance splitting criterion	ntrees = 100, minimum leaf size = 3	0.5513412167005284	00:00:38.38730	false
552	Mean Imputation, Mode Imputation, Constant Removal, Standardization	LASSO	penalty = 1.5	Support Vector Machines (SVM) of type C-SVC	kernel = 'Gaussian Kernel', cost = 0.01, gamma = 10.0	0.5	00:00:00.000	false
553	Mean Imputation, Mode Imputation, Constant Removal, Standardization	Test-Budgeted Statistically Equivalent Signature (SES)	maxK = 3, alpha = 0.05, budget = 3 * nvars	Support Vector Machines (SVM) of type C-SVC	kernel = 'Polynomial Kernel', cost = 10.0, gamma = 0.1, degree = 3	0.5	00:00:38.38725	false
554	Mean Imputation, Mode Imputation, Constant Removal, Standardization	LASSO	penalty = 1.5	Classification Random Forest with Deviance splitting criterion	ntrees = 100, minimum leaf size = 4	0.5	00:00:00.000	false
555	Mean Imputation, Mode Imputation, Constant Removal, Standardization	LASSO	penalty = 1.0	Support Vector Machines (SVM) of type C-SVC	kernel = 'Polynomial Kernel', cost = 0.1, gamma = 1.0, degree = 2	0.5888064898439392	00:00:04.4614	false
556	Mean Imputation, Mode Imputation, Constant	LASSO	penalty = 1.5	Support Vector Machines (SVM) of type C-SVC	kernel = 'Polynomial Kernel', cost = 10.0, gamma = 10.0, degree = 2	0.5	00:00:00.000	false

Configuration	Preprocessing	Name	Hyperparams	Name	Hyperparams	Performance (unadjusted)	Time (miliseconds)	Dropped
	Removal, Standardization							
557	Mean Imputation, Mode Imputation, Constant Removal, Standardization	Test-Budgeted Statistically Equivalent Signature (SES)	maxK = 2, alpha = 0.05, budget = 3 * nvars	Support Vector Machines (SVM) of type C-SVC	kernel = 'Gaussian Kernel', cost = 1.0, gamma = 1.0	0.6087025687987228	00:00:18.18113	false
558	Mean Imputation, Mode Imputation, Constant Removal, Standardization	LASSO	penalty = 1.0	Classification Random Forest with Deviance splitting criterion	ntrees = 500, minimum leaf size = 3	0.6318015205667027	00:00:04.4796	false
559	Mean Imputation, Mode Imputation, Constant Removal, Standardization	Test-Budgeted Statistically Equivalent Signature (SES)	maxK = 3, alpha = 0.01, budget = 3 * nvars	Classification Random Forest with Deviance splitting criterion	ntrees = 100, minimum leaf size = 2	0.553603436599388	00:00:03.3035	false
560	Mean Imputation, Mode Imputation, Constant Removal, Standardization	LASSO	penalty = 1.0	Support Vector Machines (SVM) of type C-SVC	kernel = 'Polynomial Kernel', cost = 1.0, gamma = 0.1, degree = 2	0.5552013972307495	00:00:04.4608	false
561	Mean Imputation, Mode Imputation, Constant Removal, Standardization	LASSO	penalty = 0.5	Classification Random Forest with Deviance splitting criterion	ntrees = 100, minimum leaf size = 4	0.7420731315468159	00:00:00.040	false
562	Mean Imputation, Mode Imputation, Constant Removal, Standardization	LASSO	penalty = 1.5	Support Vector Machines (SVM) of type C-SVC	kernel = 'Polynomial Kernel', cost = 10.0, gamma = 0.1, degree = 3	0.5	00:00:00.000	false
563	Mean Imputation, Mode Imputation, Constant Removal, Standardization	Test-Budgeted Statistically Equivalent Signature (SES)	maxK = 2, alpha = 0.05, budget = 3 * nvars	Support Vector Machines (SVM) of type C-SVC	kernel = 'Polynomial Kernel', cost = 0.01, gamma = 0.01, degree = 3	0.6836497679109014	00:00:18.18096	false
564	Mean Imputation, Mode Imputation, Constant Removal, Standardization	LASSO	penalty = 1.5	Classification Random Forest with Deviance splitting criterion	ntrees = 100, minimum leaf size = 3	0.5	00:00:00.000	false
565	Mean Imputation, Mode Imputation, Constant Removal, Standardization	Test-Budgeted Statistically Equivalent Signature (SES)	maxK = 2, alpha = 0.01, budget = 3 * nvars	Support Vector Machines (SVM) of type C-SVC	kernel = 'Polynomial Kernel', cost = 0.1, gamma = 0.1, degree = 3	0.622318122722981	00:00:00.784	false

Configuration	Preprocessing	Name	Hyperparams	Name	Hyperparams	Performance (unadjusted)	Time (miliseconds)	Dropped
566	Mean Imputation, Mode Imputation, Constant Removal, Standardization	Test-Budgeted Statistically Equivalent Signature (SES)	maxK = 3, alpha = 0.01, budget = 3 * nvars	Support Vector Machines (SVM) of type C-SVC	kernel = 'Gaussian Kernel', cost = 10.0, gamma = 10.0	0.538220551378446	00:00:03.3045	false
567	Mean Imputation, Mode Imputation, Constant Removal, Standardization	Test-Budgeted Statistically Equivalent Signature (SES)	maxK = 3, alpha = 0.05, budget = 3 * nvars	Support Vector Machines (SVM) of type C-SVC	kernel = 'Polynomial Kernel', cost = 10.0, gamma = 0.01, degree = 3	0.5	00:00:38.38725	false
568	Mean Imputation, Mode Imputation, Constant Removal, Standardization	Test-Budgeted Statistically Equivalent Signature (SES)	maxK = 3, alpha = 0.05, budget = 3 * nvars	Classification Random Forest with Deviance splitting criterion	ntrees = 100, minimum leaf size = 4	0.5364381429411793	00:00:38.38733	false
569	Mean Imputation, Mode Imputation, Constant Removal, Standardization	LASSO	penalty = 1.5	Support Vector Machines (SVM) of type C-SVC	kernel = 'Polynomial Kernel', cost = 0.1, gamma = 0.1, degree = 3	0.5	00:00:00.000	false
570	Mean Imputation, Mode Imputation, Constant Removal, Standardization	LASSO	penalty = 1.0	Support Vector Machines (SVM) of type C-SVC	kernel = 'Gaussian Kernel', cost = 1.0, gamma = 0.01	0.6015887227931763	00:00:04.4614	false
571	Mean Imputation, Mode Imputation, Constant Removal, Standardization	LASSO	penalty = 1.5	Classification Decision Tree with Deviance splitting criterion	minimum leaf size = 2, alpha = 0.01	0.5	00:00:00.000	false
572	Mean Imputation, Mode Imputation, Constant Removal, Standardization	LASSO	penalty = 0.5	Support Vector Machines (SVM) of type C-SVC	kernel = 'Polynomial Kernel', cost = 0.1, gamma = 10.0, degree = 3	0.6669608562827187	00:00:00.014	false
573	Mean Imputation, Mode Imputation, Constant Removal, Standardization	Test-Budgeted Statistically Equivalent Signature (SES)	maxK = 3, alpha = 0.05, budget = 3 * nvars	Support Vector Machines (SVM) of type C-SVC	kernel = 'Polynomial Kernel', cost = 0.01, gamma = 0.1, degree = 2	0.5446190982881267	00:00:38.38730	false
574	Mean Imputation, Mode Imputation, Constant Removal, Standardization	Test-Budgeted Statistically Equivalent Signature (SES)	maxK = 3, alpha = 0.05, budget = 3 * nvars	Classification Random Forest with Deviance splitting criterion	ntrees = 100, minimum leaf size = 2	0.5386738233651189	00:00:38.38740	false
575	Mean Imputation,	Test-Budgeted	maxK = 2, alpha = 0.01,	Support Vector	kernel = 'Gaussian	0.5120492923630575	00:00:00.789	false

Configuration	Preprocessing	Name	Hyperparams	Name	Hyperparams	Performance (unadjusted)	Time (miliseconds)	Dropped
	Mode Imputation, Constant Removal, Standardization	Statistically Equivalent Signature (SES)	budget = 3 * nvars	Machines (SVM) of type C-SVC	Kernel', cost = 0.01, gamma = 10.0			
576	Mean Imputation, Mode Imputation, Constant Removal, Standardization	Test-Budgeted Statistically Equivalent Signature (SES)	maxK = 2, alpha = 0.01, budget = 3 * nvars	Classification Decision Tree with Deviance splitting criterion	minimum leaf size = 2, alpha = 0.01	0.6811402272940734	00:00:00.791	false
577	Mean Imputation, Mode Imputation, Constant Removal, Standardization	Test-Budgeted Statistically Equivalent Signature (SES)	maxK = 3, alpha = 0.01, budget = 3 * nvars	Classification Random Forest with Deviance splitting criterion	ntrees = 100, minimum leaf size = 3	0.5514744393033866	00:00:03.3033	false
578	Mean Imputation, Mode Imputation, Constant Removal, Standardization	Test-Budgeted Statistically Equivalent Signature (SES)	maxK = 2, alpha = 0.05, budget = 3 * nvars	Ridge Logistic Regression	lambda = 0.1	0.7461900869036497	00:00:18.18167	false
579	Mean Imputation, Mode Imputation, Constant Removal, Standardization	Test-Budgeted Statistically Equivalent Signature (SES)	maxK = 2, alpha = 0.05, budget = 3 * nvars	Support Vector Machines (SVM) of type C-SVC	kernel = 'Gaussian Kernel', cost = 1.0, gamma = 10.0	0.529687260557706	00:00:18.18115	false
580	Mean Imputation, Mode Imputation, Constant Removal, Standardization	Test-Budgeted Statistically Equivalent Signature (SES)	maxK = 2, alpha = 0.01, budget = 3 * nvars	Support Vector Machines (SVM) of type C-SVC	kernel = 'Polynomial Kernel', cost = 10.0, gamma = 0.01, degree = 3	0.5490278951817413	00:00:00.780	false
581	Mean Imputation, Mode Imputation, Constant Removal, Standardization	LASSO	penalty = 1.0	Classification Random Forest with Deviance splitting criterion	ntrees = 100, minimum leaf size = 3	0.62614166085826	00:00:04.4652	false
582	Mean Imputation, Mode Imputation, Constant Removal, Standardization	Test-Budgeted Statistically Equivalent Signature (SES)	maxK = 2, alpha = 0.01, budget = 3 * nvars	Support Vector Machines (SVM) of type C-SVC	kernel = 'Polynomial Kernel', cost = 0.01, gamma = 0.1, degree = 3	0.6687526879328499	00:00:00.780	false
583	Mean Imputation, Mode Imputation, Constant Removal, Standardization	Test-Budgeted Statistically Equivalent Signature (SES)	maxK = 3, alpha = 0.05, budget = 3 * nvars	Classification Decision Tree with Deviance splitting criterion	minimum leaf size = 2, alpha = 0.01	0.532028728131967	00:00:38.38740	false
584	Mean Imputation, Mode Imputation,	Test-Budgeted Statistically Equivalent	maxK = 3, alpha = 0.05, budget = 3 * nvars	Support Vector Machines	kernel = 'Polynomial Kernel', cost = 0.01, gamma	0.5052549705130678	00:00:38.38726	false

Configuration	Preprocessing	Name	Hyperparams	Name	Hyperparams	Performance (unadjusted)	Time (miliseconds)	Dropped
	Constant Removal, Standardization	Signature (SES)		(SVM) of type C-SVC	= 0.1, degree = 3			
585	Mean Imputation, Mode Imputation, Constant Removal, Standardization	Test-Budgeted Statistically Equivalent Signature (SES)	maxK = 3, alpha = 0.05, budget = 3 * nvars	Support Vector Machines (SVM) of type C-SVC	kernel = 'Gaussian Kernel', cost = 0.01, gamma = 1.0	0.5310374370344007	00:00:38.38732	false
586	Mean Imputation, Mode Imputation, Constant Removal, Standardization	LASSO	penalty = 0.5	Support Vector Machines (SVM) of type C-SVC	kernel = 'Gaussian Kernel', cost = 0.01, gamma = 0.01	0.7016631816226958	00:00:00.012	false
587	Mean Imputation, Mode Imputation, Constant Removal, Standardization	Test-Budgeted Statistically Equivalent Signature (SES)	maxK = 3, alpha = 0.05, budget = 3 * nvars	Support Vector Machines (SVM) of type C-SVC	kernel = 'Polynomial Kernel', cost = 0.1, gamma = 0.1, degree = 3	0.49840132876570126	00:00:38.38726	false
588	Mean Imputation, Mode Imputation, Constant Removal, Standardization	Test-Budgeted Statistically Equivalent Signature (SES)	maxK = 3, alpha = 0.01, budget = 3 * nvars	Classification Random Forest with Deviance splitting criterion	ntrees = 100, minimum leaf size = 2	0.5513534205335825	00:00:03.3033	false
589	Mean Imputation, Mode Imputation, Constant Removal, Standardization	LASSO	penalty = 1.0	Support Vector Machines (SVM) of type C-SVC	kernel = 'Polynomial Kernel', cost = 10.0, gamma = 1.0, degree = 2	0.5722968046348614	00:00:04.4613	false
590	Mean Imputation, Mode Imputation, Constant Removal, Standardization	LASSO	penalty = 1.5	Support Vector Machines (SVM) of type C-SVC	kernel = 'Gaussian Kernel', cost = 0.01, gamma = 1.0	0.5	00:00:00.000	false
591	Mean Imputation, Mode Imputation, Constant Removal, Standardization	LASSO	penalty = 1.5	Classification Random Forest with Deviance splitting criterion	ntrees = 100, minimum leaf size = 2	0.5	00:00:00.000	false
592	Mean Imputation, Mode Imputation, Constant Removal, Standardization	LASSO	penalty = 0.5	Support Vector Machines (SVM) of type C-SVC	kernel = 'Linear Kernel', cost = 0.01	0.6967129979781802	00:00:00.011	false
593	Mean Imputation, Mode Imputation, Constant	LASSO	penalty = 1.5	Support Vector Machines (SVM) of type C-SVC	kernel = 'Polynomial Kernel', cost = 0.01, gamma = 0.1, degree = 2	0.5	00:00:00.000	false

Configuration	Preprocessing	Name	Hyperparams	Name	Hyperparams	Performance (unadjusted)	Time (miliseconds)	Dropped
	Removal, Standardization							
594	Mean Imputation, Mode Imputation, Constant Removal, Standardization	LASSO	penalty = 1.0	Classification Random Forest with Deviance splitting criterion	ntrees = 100, minimum leaf size = 4	0.6302933739674631	00:00:04.4639	false
595	Mean Imputation, Mode Imputation, Constant Removal, Standardization	Test-Budgeted Statistically Equivalent Signature (SES)	maxK = 3, alpha = 0.05, budget = 3 * nvars	Support Vector Machines (SVM) of type C-SVC	kernel = 'Polynomial Kernel', cost = 0.01, gamma = 10.0, degree = 2	0.4934207127780002	00:00:38.38726	false
596	Mean Imputation, Mode Imputation, Constant Removal, Standardization	Test-Budgeted Statistically Equivalent Signature (SES)	maxK = 3, alpha = 0.01, budget = 3 * nvars	Support Vector Machines (SVM) of type C-SVC	kernel = 'Polynomial Kernel', cost = 10.0, gamma = 10.0, degree = 2	0.5	00:00:03.3028	false
