# Supplementary material for: Machine learning approaches in microbiome research: challenges and best practices
Source: Front Microbiol. 2023 Sep 22;14:1261889. doi: 10.3389/fmicb.2023.1261889 (PMC10556866; doi:10.3389/fmicb.2023.1261889)
Supplement: Supplementary file 2 [file Data_Sheet_2.docx]

Supplementary Material

# Comparison of data preprocessing strategies

Prior to performing an exhaustive benchmarking of Machine Learning (ML) classification pipelines on the CRC datasets selected by the consortium, we conducted a preliminary study on preprocessing strategies on six different public shotgun sequencing microbiome datasets obtained from (Pasolli et al., 2016) to check whether the preprocessing choice may depend on the characteristics of the dataset. We focused on comparing the four combinations of two low-abundance filtering approaches and two normalization methods, after removing outliers above the 99% confidence limit for Hotelling’s T2 statistic computed from PCA scores. The first low-abundance filtering consisted of simply removing species with zero counts in all samples, while the second prevalence filter removed species with less than 20% non-zero values in both case and control groups. The two normalization methods aimed to deal with the compositional nature of microbiome data and remove the library size bias: one was the Total Sum Scaling (TSS) followed by a logarithmic transformation and the other was the Aitchison’s Centered Log Ratio (CLR) transformation (Aitchison, 1986). To evaluate the performance of ML methods under these preprocessing options, we selected two rather different classification models: PLS-DA, a linear and interpretable model, and Random Forest, a non-linear but less interpretable option. We optimized the probability threshold for each dataset, normalization strategy and model within the k-fold CV procedure (k=10 and 5 repeats), along with specific hyperparameters of each model. The F1-score was the metric computed to measure the classification error.

We found statistically significant differences when comparing the four preprocessing strategies (Supplementary Figure 1A), being CLR with the 20% prevalence filter the one with the best performance (F1 = 0.710) and zero-filtering with TSS the worst one (F1=0.676), although the magnitude of the difference is not so large. RF was less affected by the preprocessing strategy than PLS-DA, although it performed better with CLR transformation and filtering was not so important (Supplementary Figure 1B). The low-abundance filtering had a higher impact on PLS-DA performance than the normalization method, being the prevalence filter the one with the best performance (Supplementary Figure 1B). Finally, we observed that the impact of preprocessing was quite different depending on the database. Non-significant effects were found for 4 of the databases, but for the two databases with more imbalance between case and control groups the prevalence filtering combined with CLR transformation rendered the best performance (Supplementary Figure 1C). Although sparsity was high (around 78% of zeroes) in all databases, these two databases presented a sparsity level below the median (Supplementary Figure 1D). In fact, we observed the higher the sparsity in the data, the better models discriminate (Supplementary Figure 1E).

**References**

Aitchison, J. (1986). The statistical analysis of compositional data. Chapman and Hall. http://swbplus.bsz-bw.de/bsz01244457Xinh.htm

Pasolli, E., Truong, D. T., Malik, F., Waldron, L., & Segata, N. (2016). Machine Learning Meta-analysis of Large Metagenomic Datasets: Tools and Biological Insights. PLOS Computational Biology, 12(7), e1004977. https://doi.org/10.1371/journal.pcbi.1004977

# Supplementary Figures and Tables

**Supplementary Figure 1.** A) Comparison of F1-score values (X-axis) for the four pre-processing strategies coming from combining low-abundance filters with normalization methods: S1 (filtering out features with zeroes in all samples); S2 (20% prevalence filter); N1 (TSS normalization); N2 (CLR transformation). A pair of non-overlapping red arrows correspond to two strategies rendering a statistically significant difference between F1-scores. (B) Comparison of F1-score values (X-axis) for the combination of pre-processing strategies and ML methods: RF (Random Forest); PLS-DA (Partial Least Squares Discriminant Analysis). A pair of non-overlapping red arrows correspond to two strategies rendering a statistically significant difference between F1-scores. (C) Mean F1-score values (Y-axis) for each pre-processing strategy and database: CIRR (cirrhosis); CRC (colorectal cancer); IBD (inflammatory bowel disease); OBES (obesity); T2D (Type 2 diabetes for Chinese subjects); T2D (Type 2 diabetes for European women). (D) Level of sparsity (proportion of zeroes) for each database. The dotted grey line is the median value. (E) Mean F1-score (Y-axis) for each ML method at different sparsity levels: L (low, below the median); H (high, above the median).


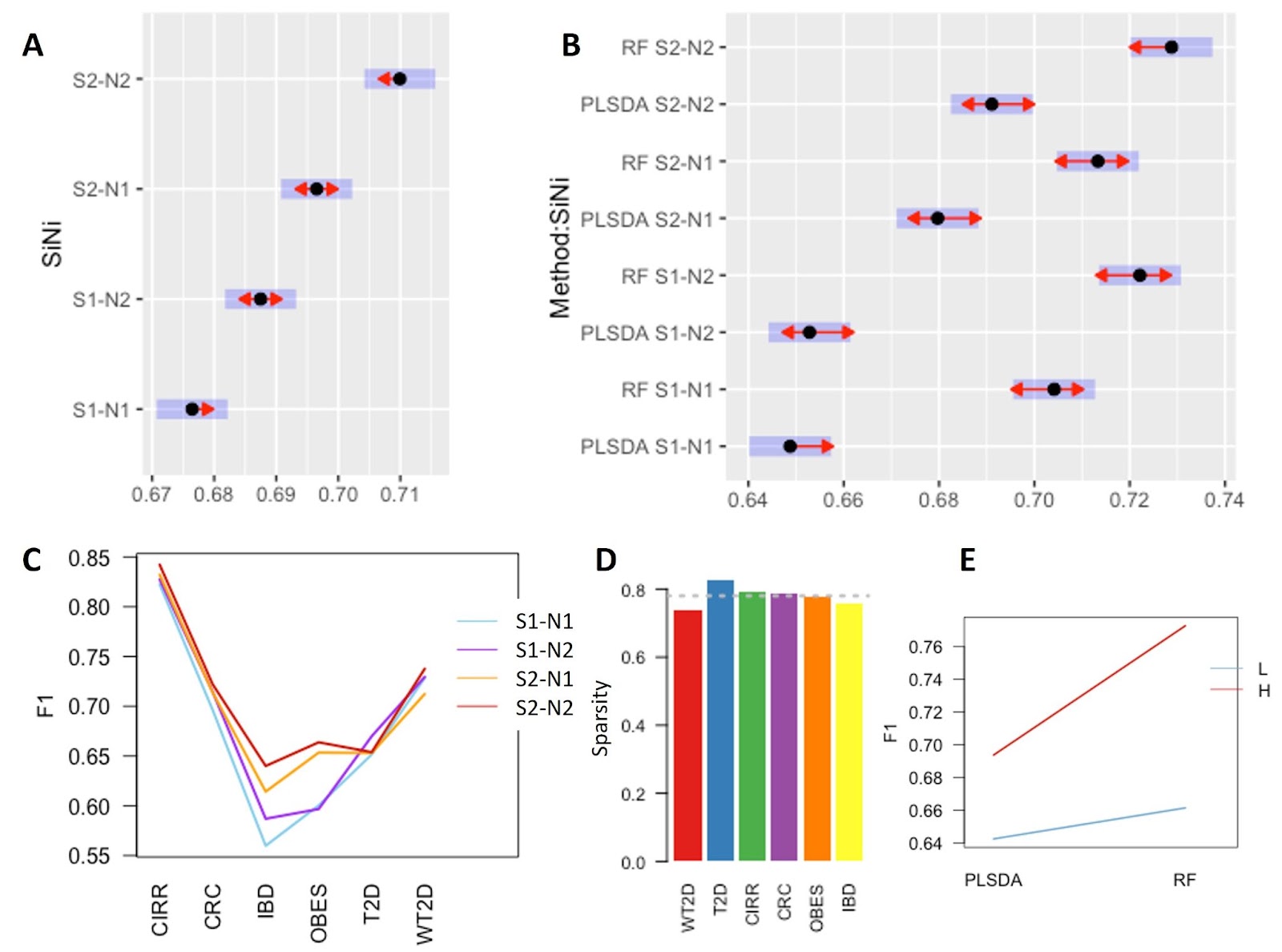


**Supplementary Table 1.** Description of metadata features.

| **Name** | **Description** |
| --- | --- |
| Study_accession | NCBI BioProject number |
| Age | Age in years |
| HQ_clean_read_count | Number of reads after filtering and cleaning steps (low quality and contaminating human reads removed), used to quantify species |
| Mapped_read_count | Number of mapped read |
| Instrument_mode | Sequencing platform used (Illumina HiSeq / NextSeq) |
| Timepoint | Time of sampling for longitudinal data |
| Westernised | W = westernised / NW = not westernised |
| Country | Austria (AUT), China (CHN), France (FRA), Germany (GER), India (IND), Italy (ITA), Japan (JPN), USA |
| Gender | Male/Female |

**Supplementary Table 2.** IDs of metagenomic species (MSPs) and their corresponding taxonomic labels.

| **MSP** | **Taxonomic label** |
| --- | --- |
| msp_1081 | Fusobacterium gonidiaformans |
| msp_0578 | [Clostridium] sp. CAG:269 |
| msp_0622 | unclassified Christensenellales CAG-74 |
| msp_0833 | Streptococcus thermophilus |
| msp_0610 | Fusobacterium nucleatum subsp. animalis |
| msp_0100 | Morganella morganii |
| msp_1579c | unclassified Ruminococcaceae |
| msp_0676 | unclassified Oscillospirales 1 CAG-272 |
| msp_0236 | [Eubacterium] ventriosum |
| msp_1010 | unclassified Anaerotignaceae CAG-274 |
| msp_0317 | unclassified Oscillospirales 4 UBA5446 |
| msp_0757 | [Clostridium] sp. 28_12 & CAG:356 |
| msp_0910 | Bifidobacterium catenulatum |
| msp_0496 | unclassified Lachnospira |
| msp_0574c | Fusobacterium necrophorum |
| msp_1327 | Peptostreptococcus stomatis |
| msp_1028c | Prevotella intermedia |
| msp_0938 | [Firmicutes] bacterium CAG:536 |
| msp_0126 | [Ruminococcus] torques |
| msp_0129 | [Clostridium] sp. CAG:58 |
| msp_1188 | Ruminococcaceae bacterium CIM:MAG 577 |
| msp_0172 | Ruthenibacterium lactatiformans |
| msp_1069 | unclassified Clostridiales 2a |
| msp_0257 | Prevotella sp. UBA634 |
| msp_0835 | Clostridiales bacterium 41_21_two_genomes |
| msp_1324 | unclassified Clostridiales 2a |
| msp_1682c | Clostridioides difficile |
| msp_0864 | Homo sapiens |
| msp_1102 | Firmicutes bacterium CAG:552 |
| msp_1467 | unclassified Methanomethylophilaceae |
| msp_1245 | Parvimonas sp. KA00067 |
| msp_0668 | Coprococcus sp. CAG:782 |
| msp_1158 | msp_1158 - Porphyromonas somerae |
| msp_0305 | [Roseburia] sp. CAG:303 |
| msp_0937 | unclassified Duodenibacillus |
| msp_1671c | Neglecta timonensis |
| msp_1790 | Fusobacterium nucleatum subsp. polymorphum |
| msp_0110 | Bilophila wadsworthia |
| msp_1754 | [Raoultella] ornithinolytica |
| msp_0062 | msp_0062 - Odoribacter splanchnicus |
| msp_0814 | [Lachnoclostridium] sp. An138 |
| msp_0853c | Granulicatella adiacens |
| msp_1322 | [Clostridium] sp. CAG:1193 |
| msp_1217 | Eggerthella sp. CAG:1427 |
| msp_1156 | Solobacterium moorei |
| msp_1036 | unclassified Dialister |
| msp_0805 | unclassified Roseburia |
| msp_1712 | unclassified Monoglobaceae |
| msp_1231 | unclassified Bacilli RF39 CAG-1000 CAG-533 |
| msp_0454 | unclassified Clostridium |
| msp_0935 | Peptostreptococcus anaerobius |
| msp_1657 | unclassified Clostridiales 2a |
| msp_1234 | Parvimonas micra |
| msp_0076 | Blautia wexlerae |
| msp_1487 | unclassified Clostridiales 2d |
| msp_1570 | Porphyromonas asaccharolytica |
| msp_1042 | unclassified Bacilli RF39 CAG-611 UBA7057 |
| msp_0118 | unclassified Bacteroides |
| msp_1112 | [Mycoplasma] sp. CAG:956 |
| msp_0457 | Faecalibacterium prausnitzii 9 |
| msp_1048 | unclassified Enterocloster |
| msp_0232 | Dorea sp. CAG:317 |
| msp_0542 | [Clostridiaceae] bacterium CIM:MAG 755 / [Clostridium] sp. CAG:230 |
| msp_0468 | Firmicutes bacterium CAG:41 / [Clostridium] sp. 2789STDY5834935 & sp. 2789STDY5608853 |
| msp_0258 | Anaerotignum faecicola |
| msp_1789 | Alloprevotella tannerae |
| msp_1173c | unclassified Oscillospiraceae |
| msp_0347 | Clostridium paraputrificum |
| msp_0089 | Alistipes finegoldii |

**Supplementary Table 3**. IDs of metagenomic species (MSPs) and their corresponding taxonomic labels for all the cohorts taken together, each cohort separately, the best performing model and the best interpretation model (Separate Excel spreadsheet).

**
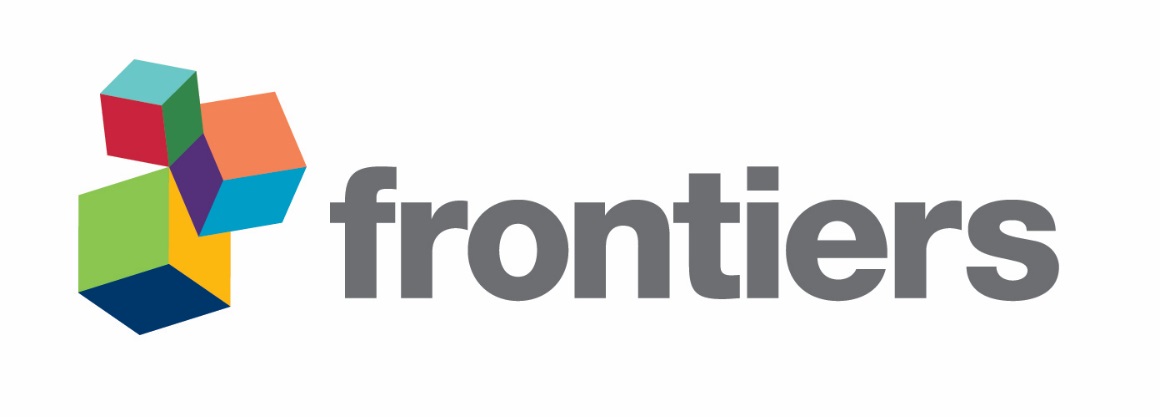
**
